# Supplementary material for: Solvent Moisture-Controlled Self-Assembly of Fused Benzoimidazopyrrolopyrazines with Different Ring’s Interposition
Source: Molecules. 2022 Apr 11;27(8):2460. doi: 10.3390/molecules27082460 (PMC9024467; doi:10.3390/molecules27082460)
Supplement: Supplementary file 1 [file molecules-27-02460-s001.zip › molecules-1654462-supplementary.pdf]

Supplementary Materials

Solvent Moisture-Controlled Self-Assembly of Fused Benzoimidazopyrrolopyrazines with Different Ring's Interposition

Svetlana V. Martynovskaya, Arsalan B. Budaev, Igor A. Ushakov, Tatyana N. Borodina and Andrey V. Ivanov *

A.E. Favorsky Irkutsk Institute of Chemistry, Siberian Branch of the Russian Academy of Sciences, 1 Favorsky St., 664033 Irkutsk, Russia

* E-mail: ivanov@irioch.irk.ru

Table of contents

1. Procedure for the synthesis intermediate H. S3

2. NMR spectra of the obtained compounds 3 ....S6

3. NMR spectra of the obtained compounds 4 .... S16

4. NMR spectra of the obtained compounds 5 .... S26

5. NMR spectra of the obtained compounds 6 .... S35

6. Selected 2D NMR spectra S46

7. X-ray crystallographic data of 3e and 6d.. S54

8. References S60

1. Procedure for the synthesis intermediate H.

Synthesis S1.

A mixture of (5-phenyl-1-(propa-1,2-dien-1-yl)-1*H*-pyrrol-2-yl)methanol (0.0011 mol), *o*-phenylenediamine (0.13 g, 0.00121 mol), EtOH (2.2 mL), H_2O (0.44 mL) and CF_3COOH (1%) was stirred at r.t. for 16 h. As a result, a mixture of the starting alcohol, *o*-phenylenediamine and the product was obtained. When the reaction was carried out in ethanol for more than 16 h, the reaction mixture became resinous.

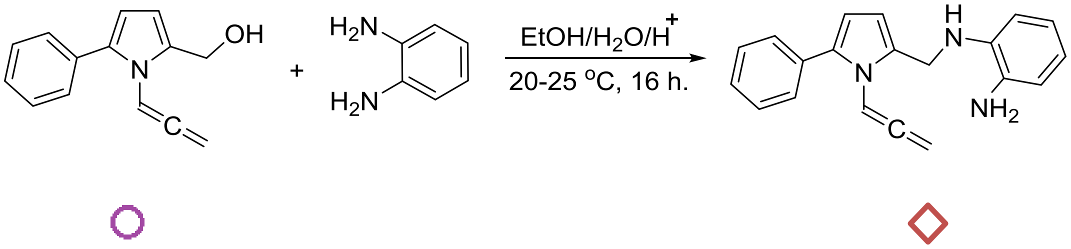

^{1}H-NMR (400 MHz, $CDCl_3$) spectrum of intermediate **H**

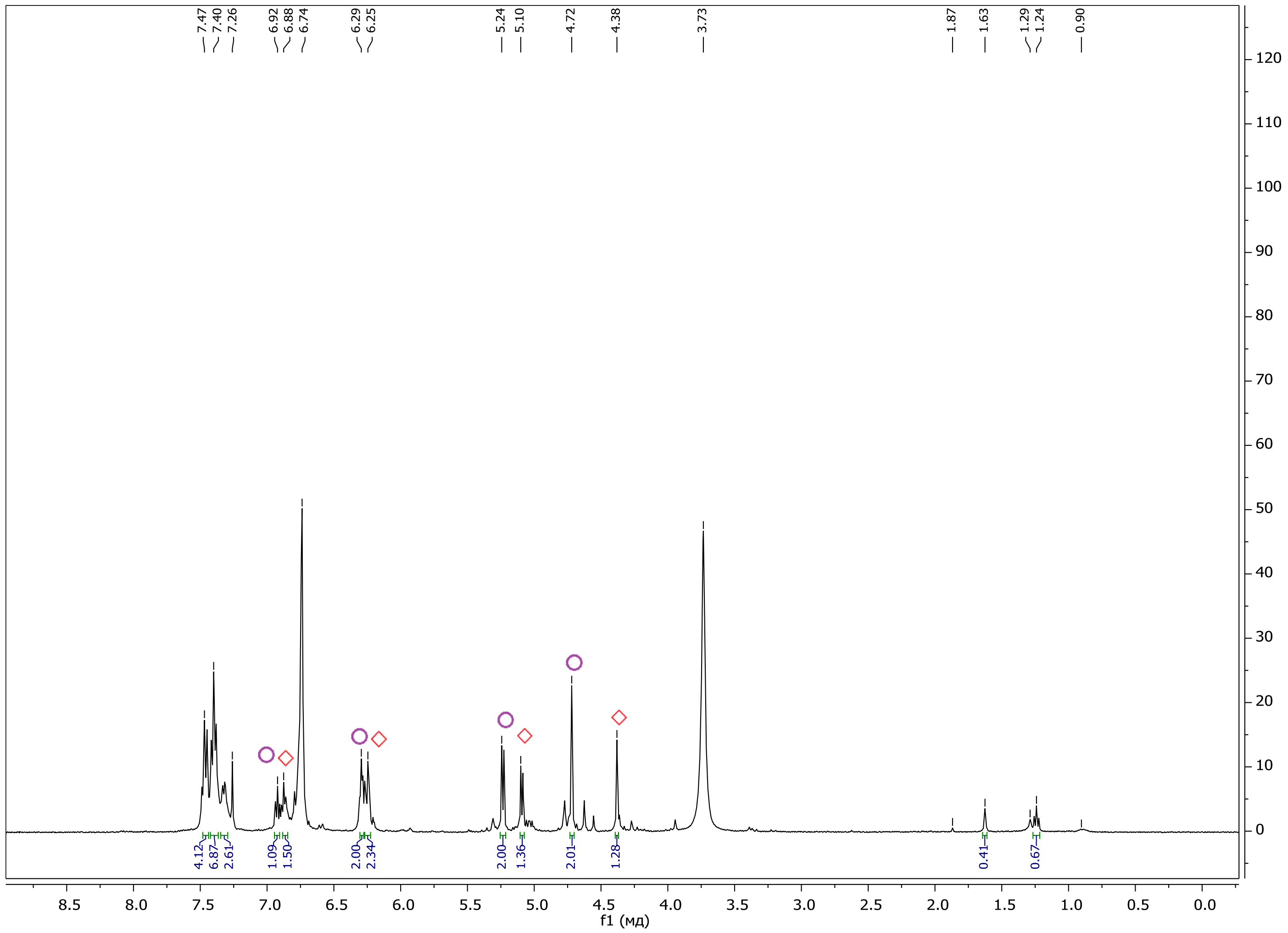

Figure S1. 2D NOESY spectrum of reaction mixture of H.

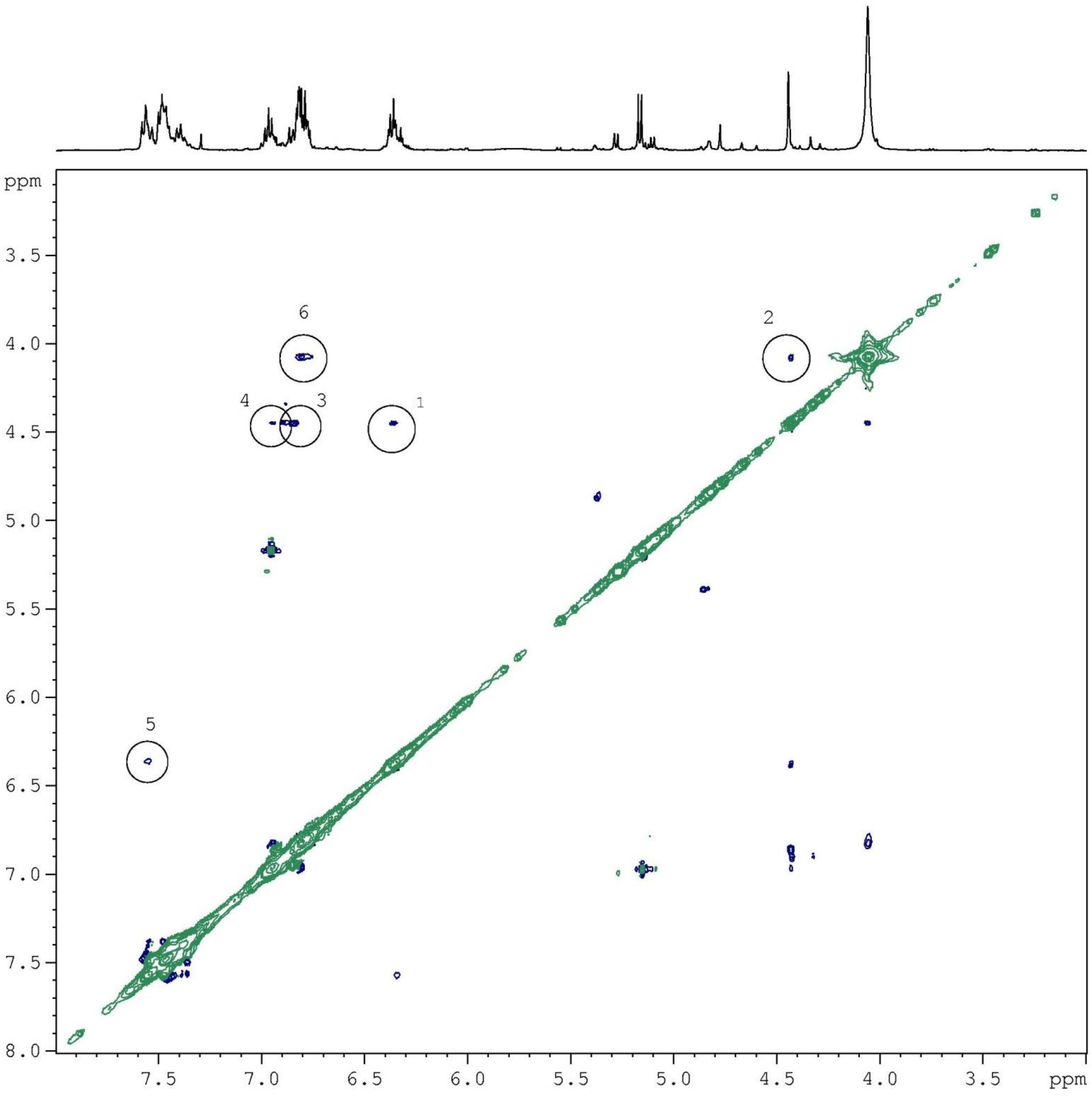

The numbers correspond to the correlations below.

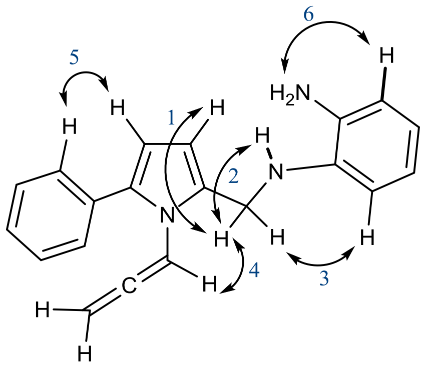

Main NOE correlations

Figure S2. 2D Spectrum ^{1}H-^{13}C HMBC reaction mixture.

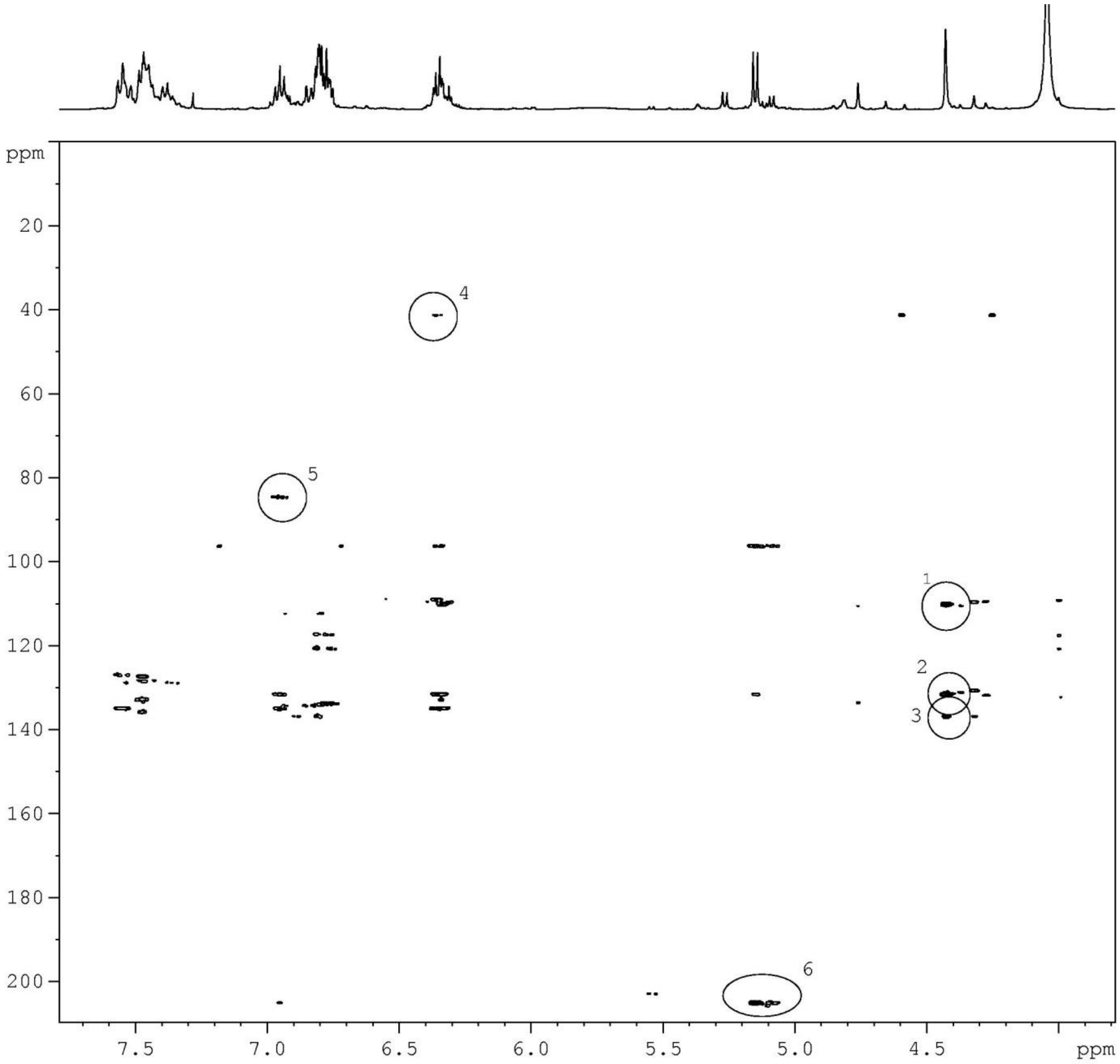

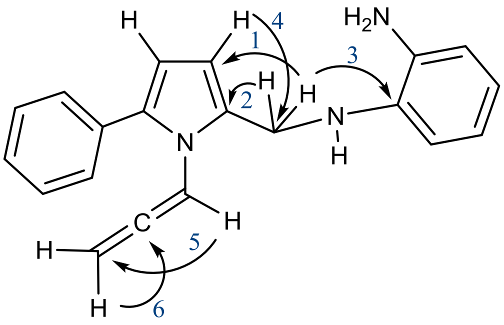

Main HMBC correlations

2. NMR spectra of the obtained compounds 3

Figures S3. ^{1}H-NMR (400 MHz, $CDCl_3$) spectrum of **3a**

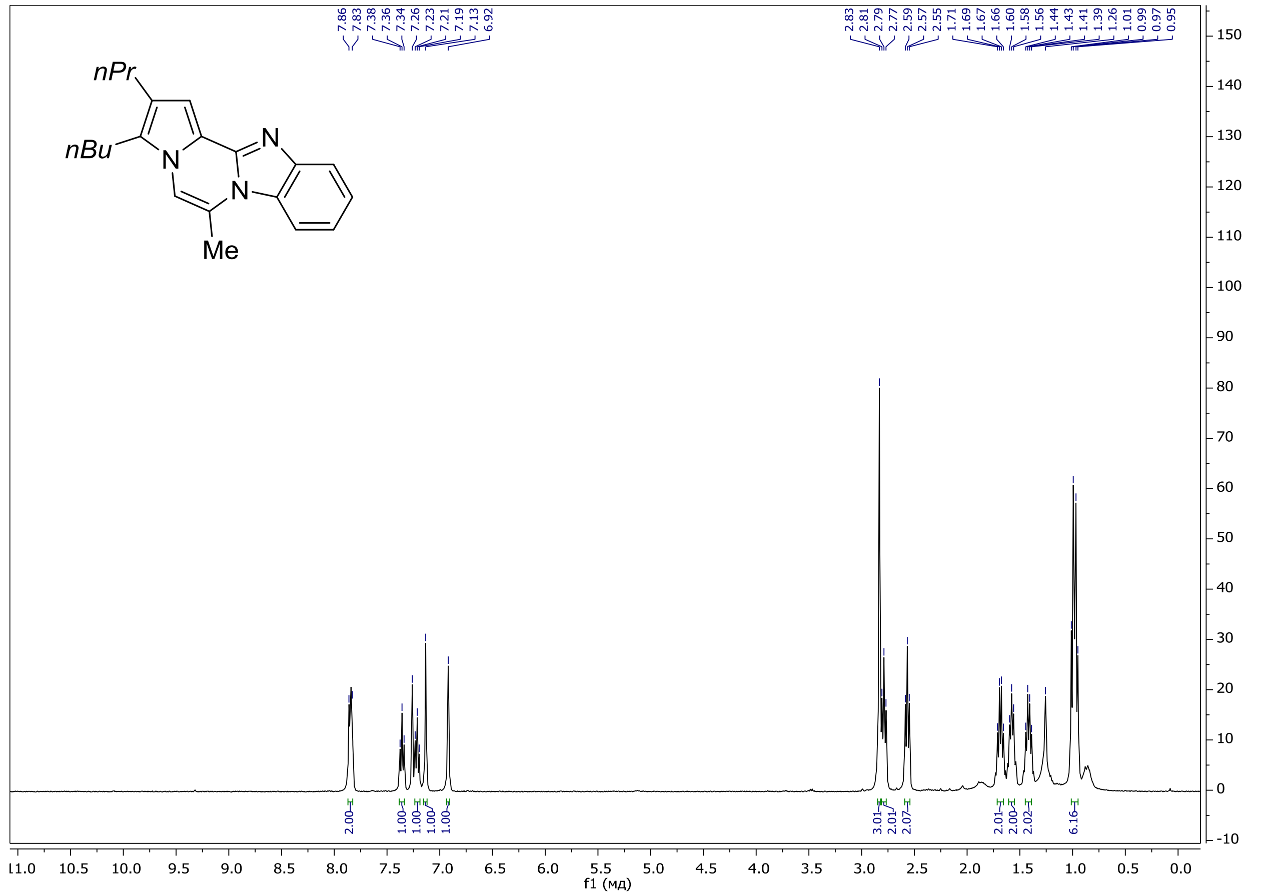

Figure S4. ^{13}C-NMR (100 MHz, $CDCl_3$) spectrum of **3a**

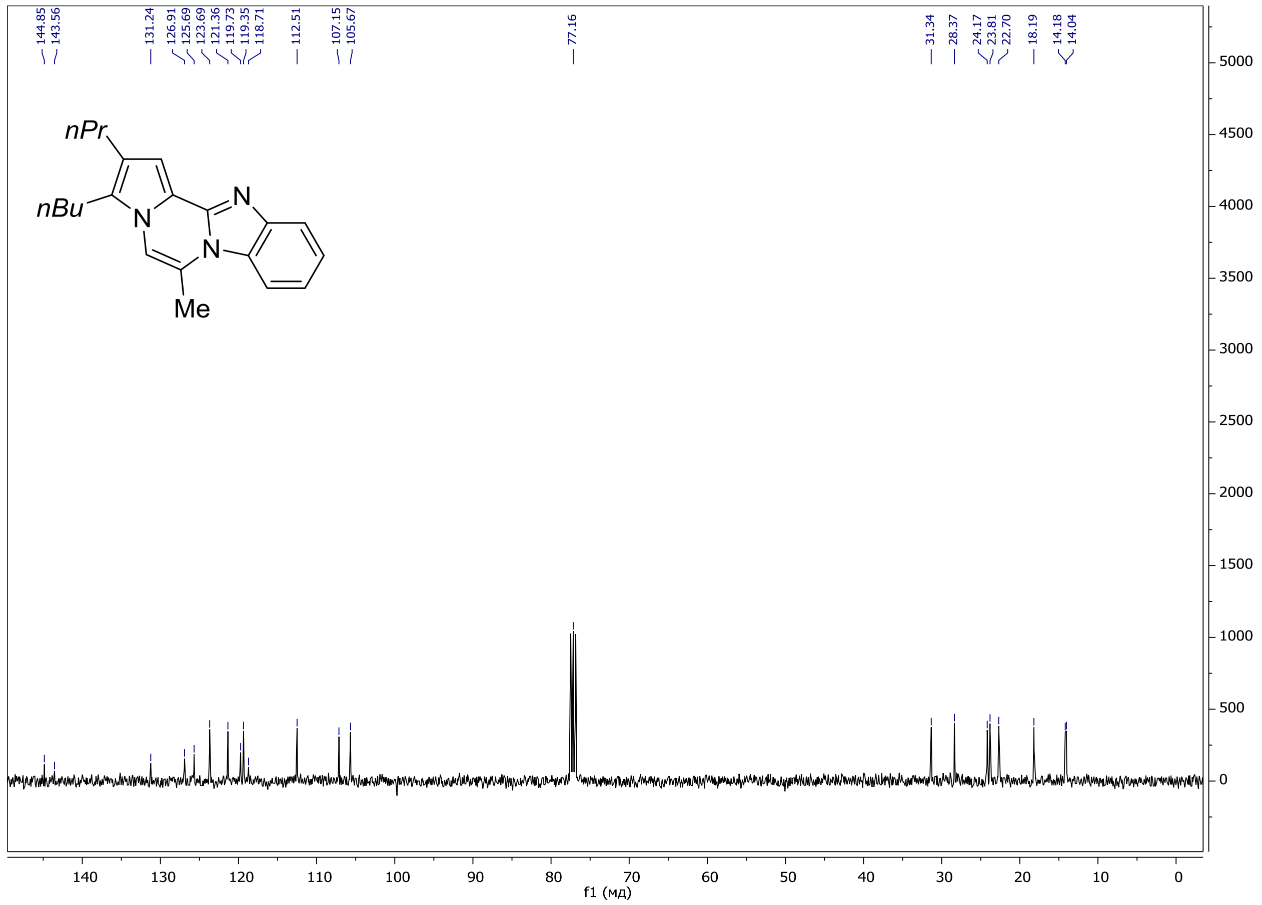

Figure S5. ^{1}H-NMR (400 MHz, $CDCl_3$) spectrum of **3b**

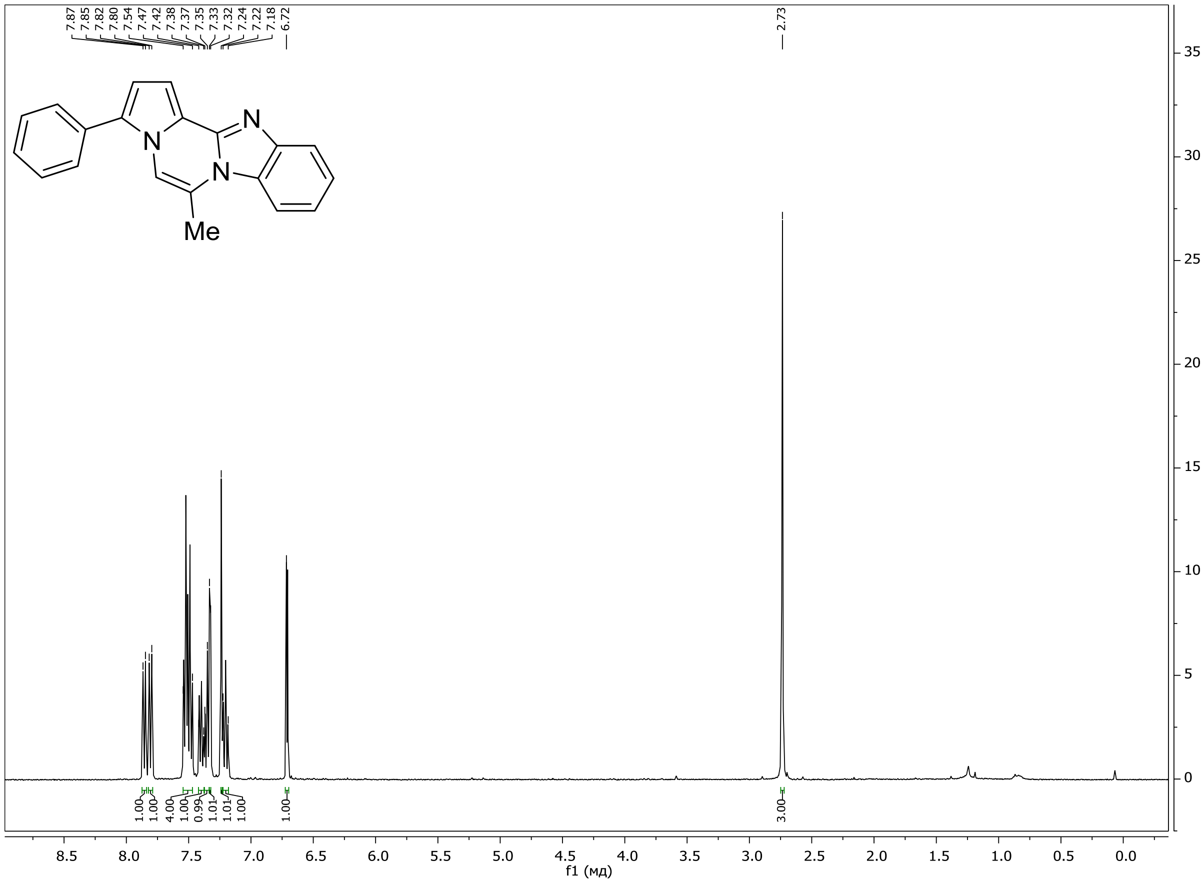

Figure S6. ^{13}C-NMR (100 MHz, $CDCl_3$) spectrum of **3b**

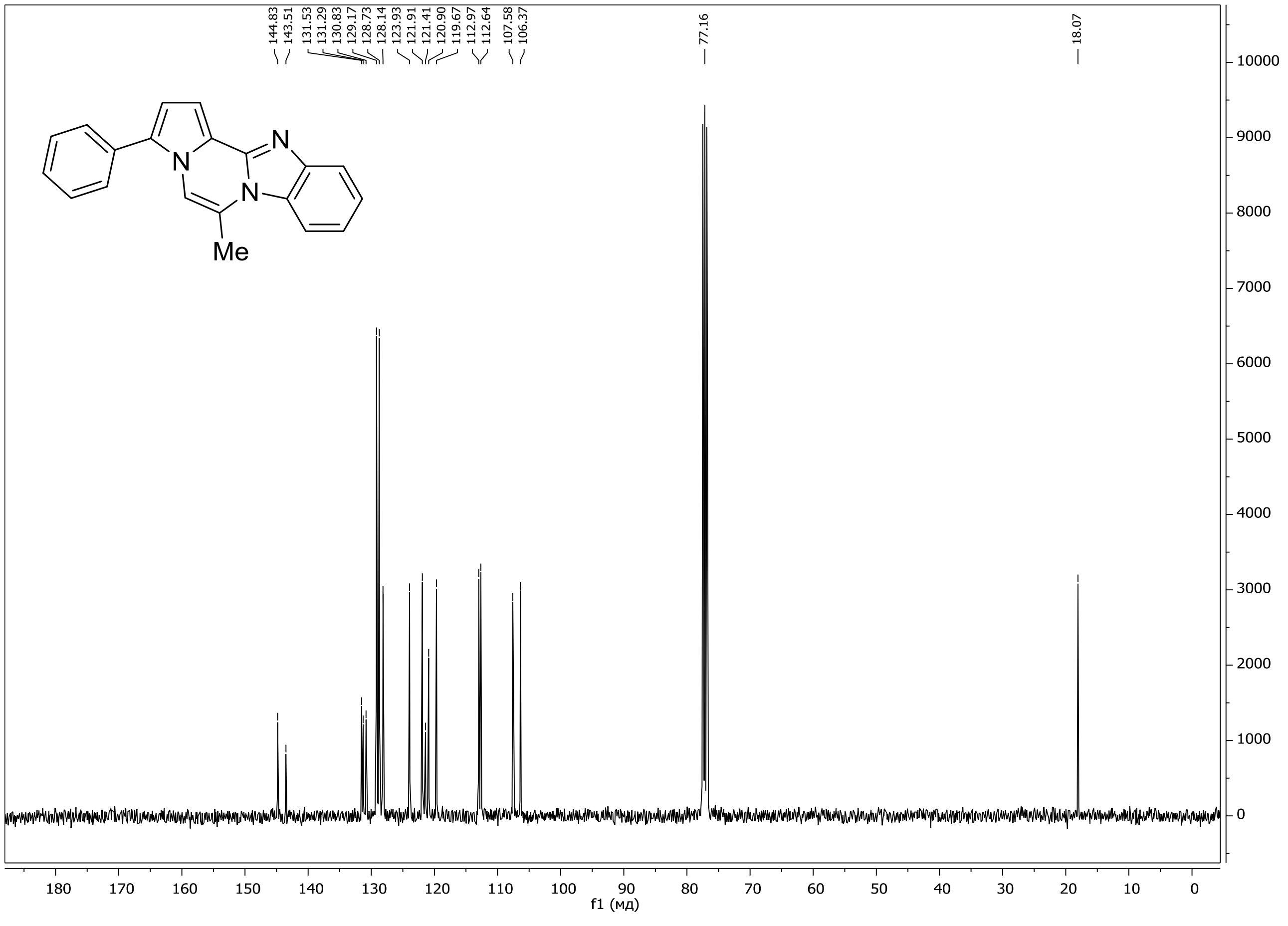

Figure S7. ^{1}H-NMR (400 MHz, $CDCl_3$) spectrum of **3c**

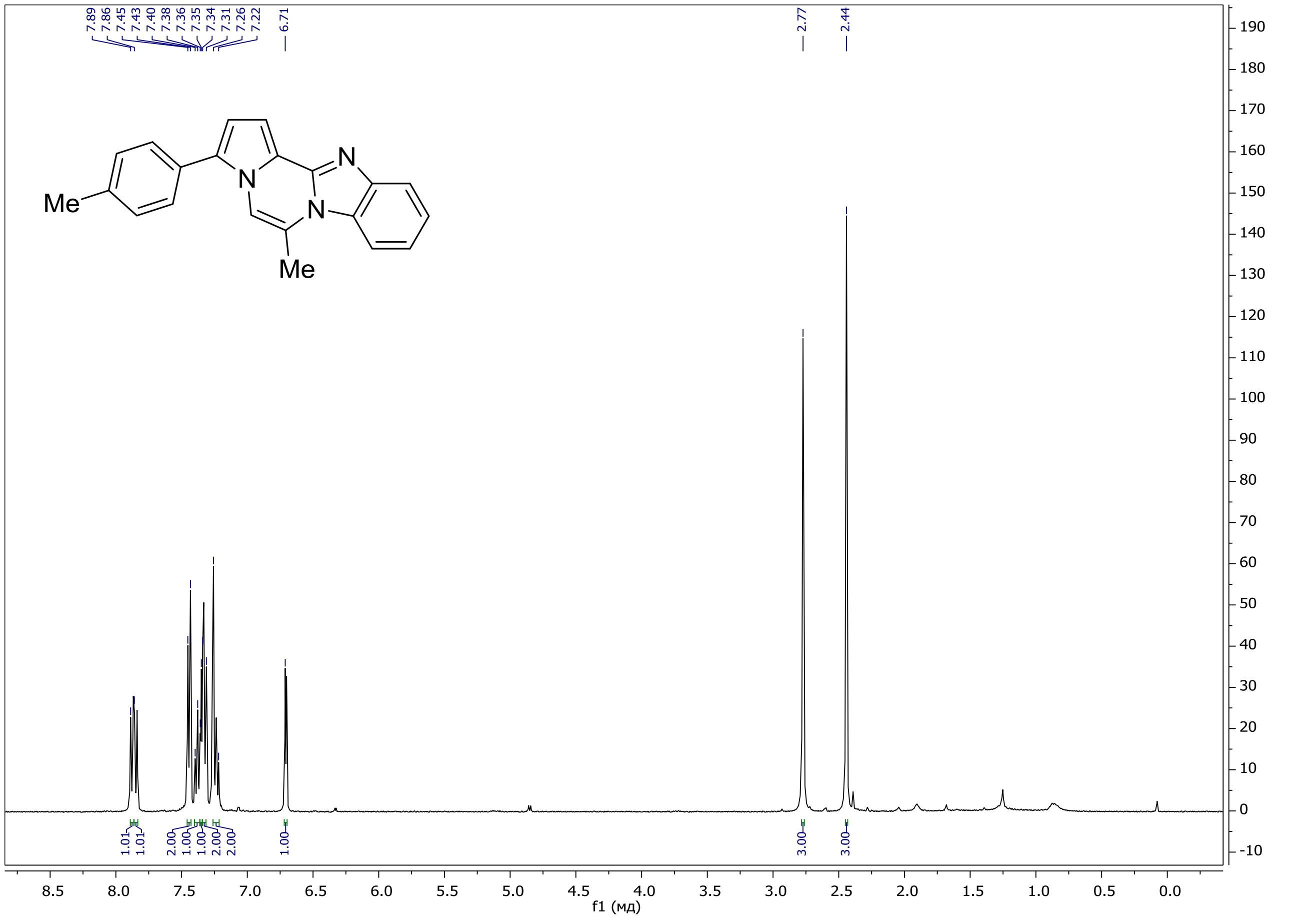

Figure S8. ^{13}C-NMR (100 MHz, $CDCl_3$) spectrum of **3c**

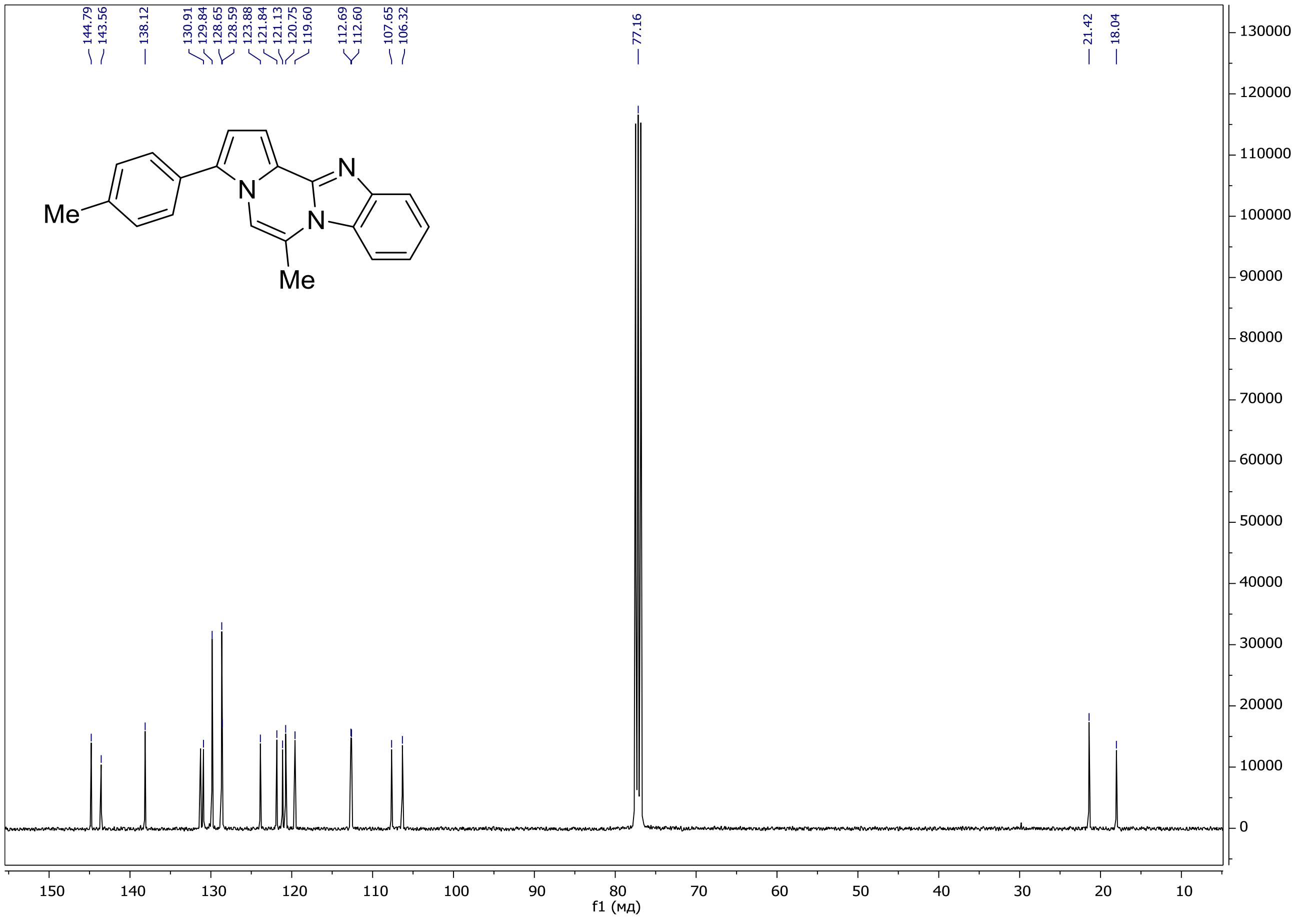

Figure S9. ^{1}H-NMR (400 MHz, $CDCl_3$) spectrum of **3d**

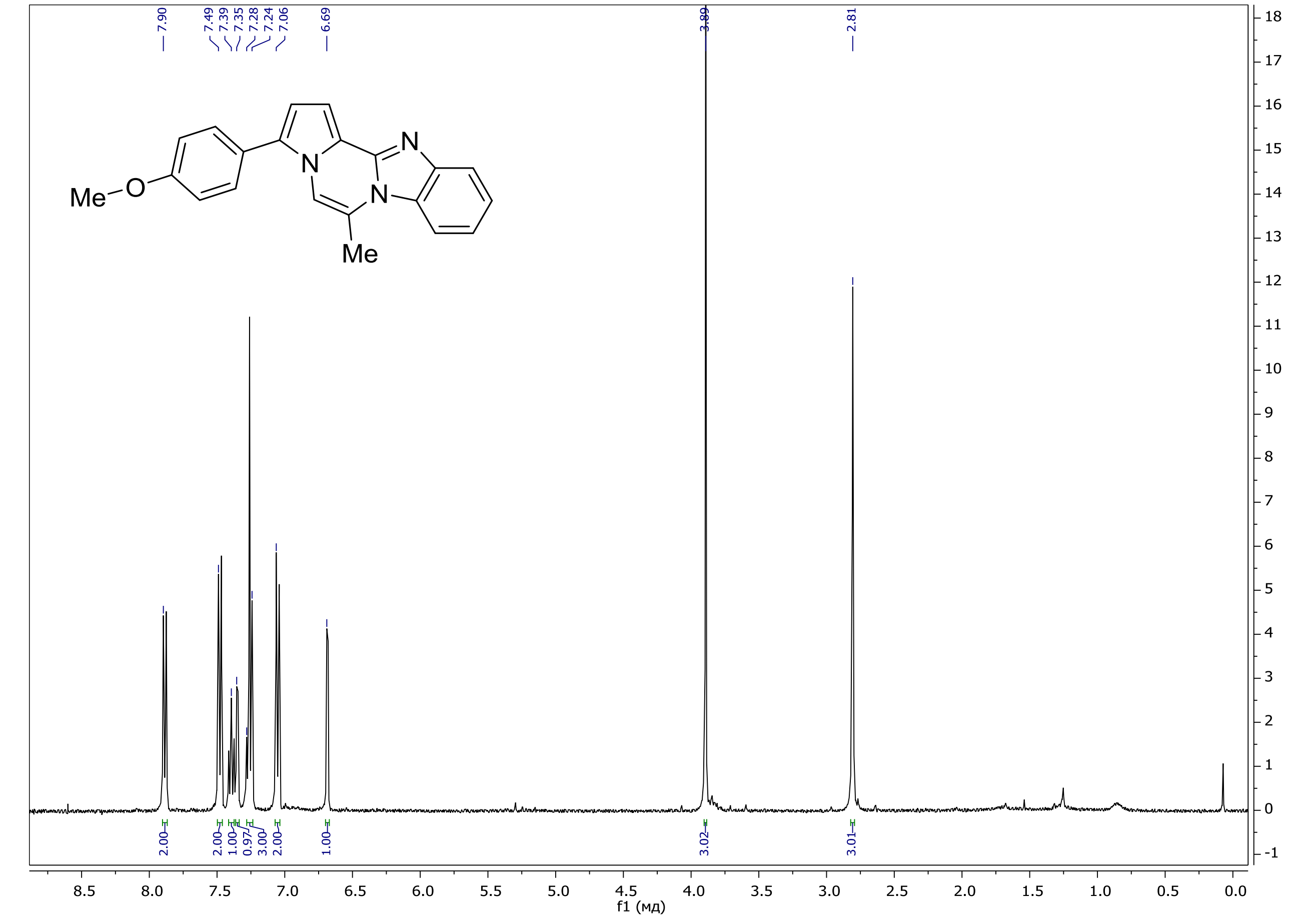

Figure S10. ^{13}C-NMR (100 MHz, $CDCl_3$) spectrum of **3d**

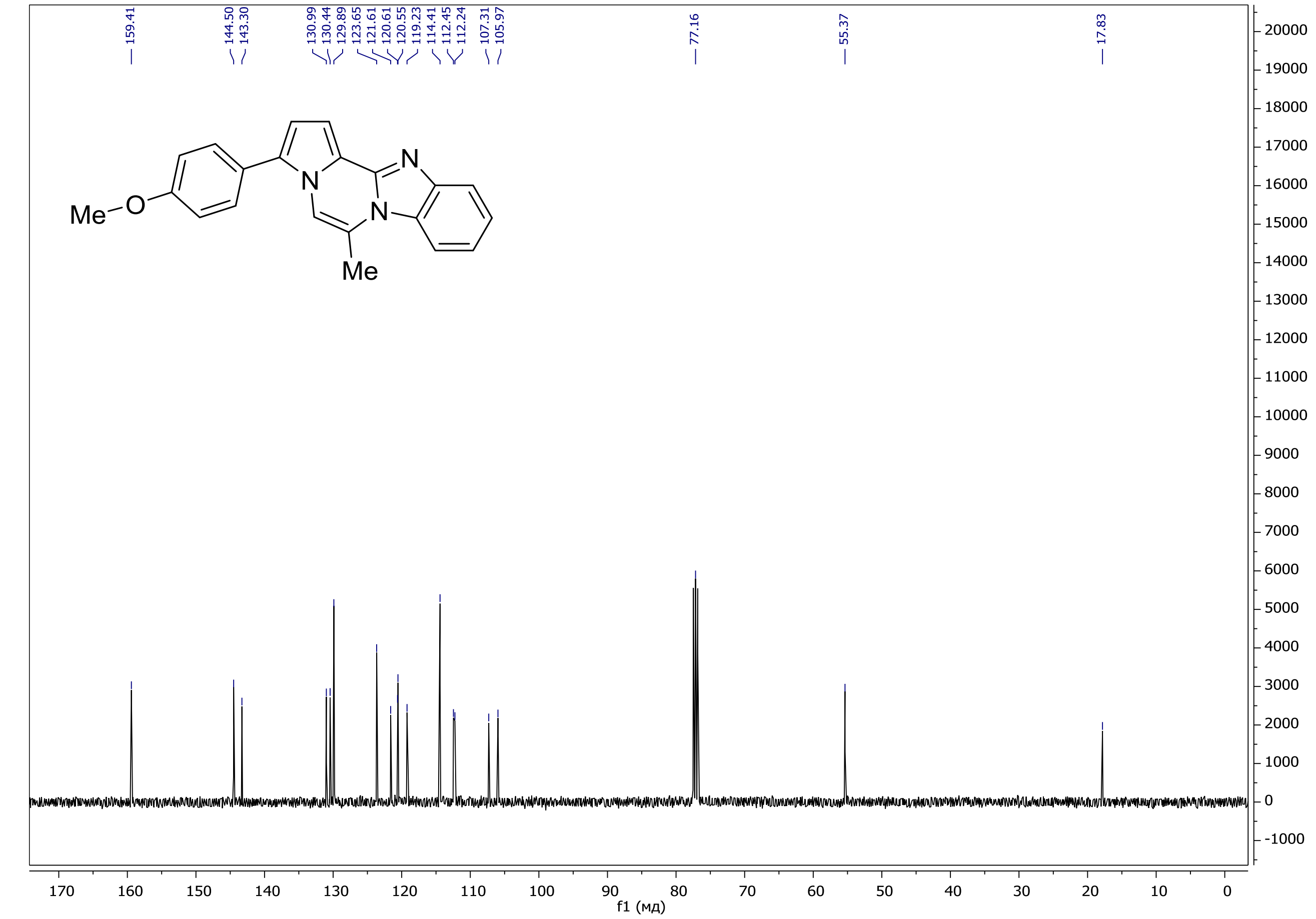

Figure S11. ^{1}H-NMR (400 MHz, $CDCl_3$) spectrum of **3e**

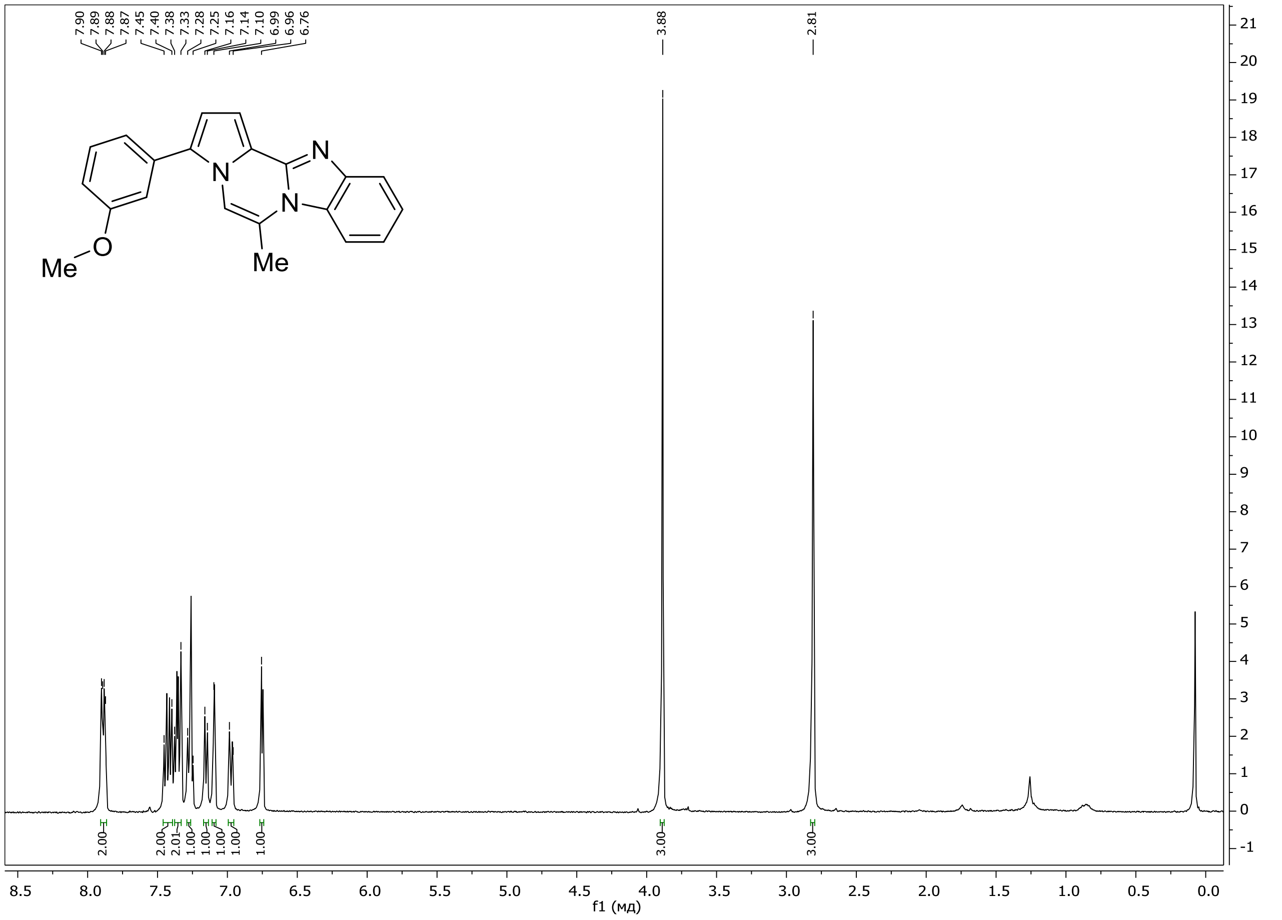

Figure S12. ^{13}C-NMR (100 MHz, $CDCl_3$) spectrum of **3e**

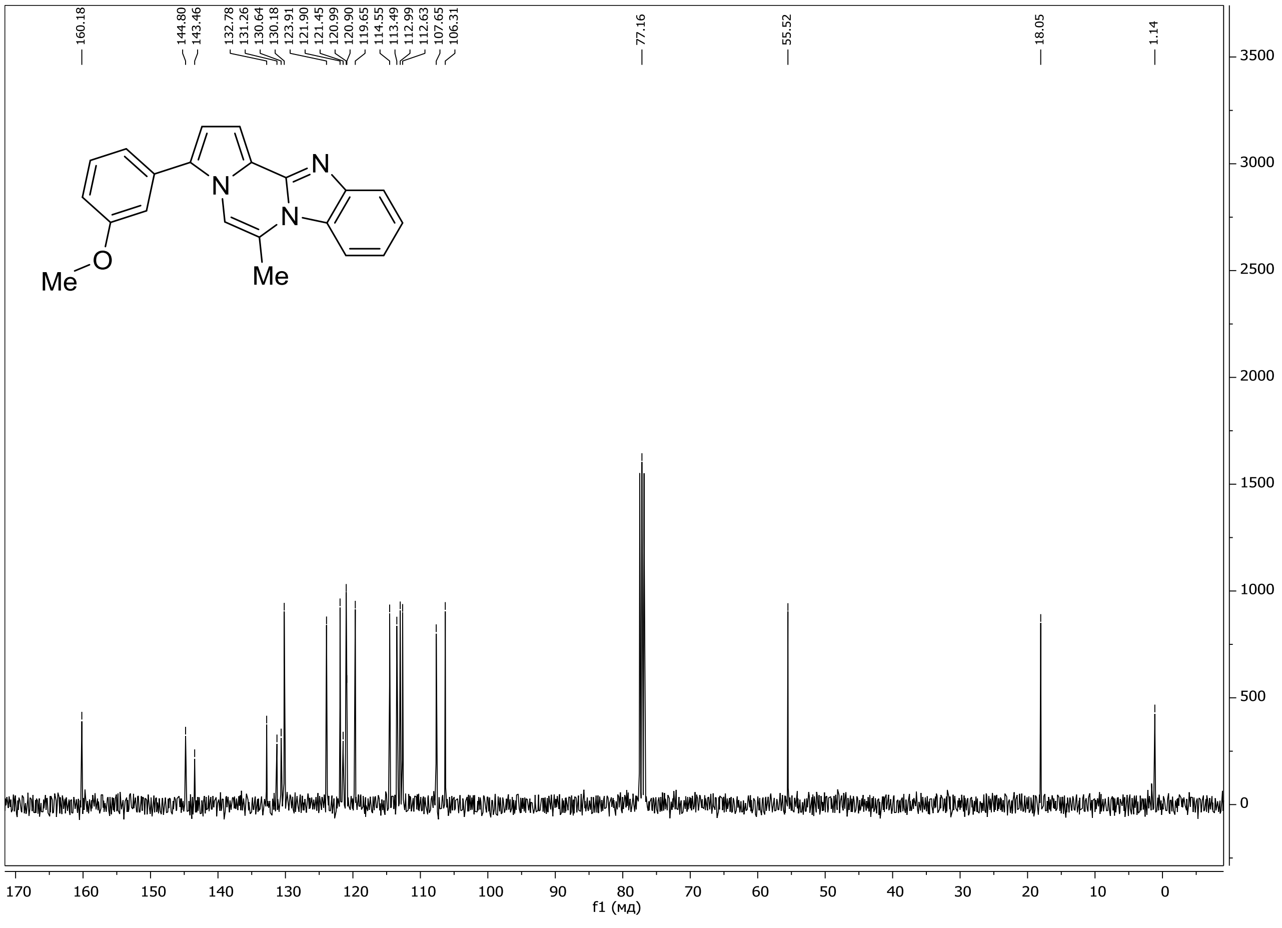

Figure S13. ^{1}H-NMR (400 MHz, $CDCl_3$) spectrum of **3f**

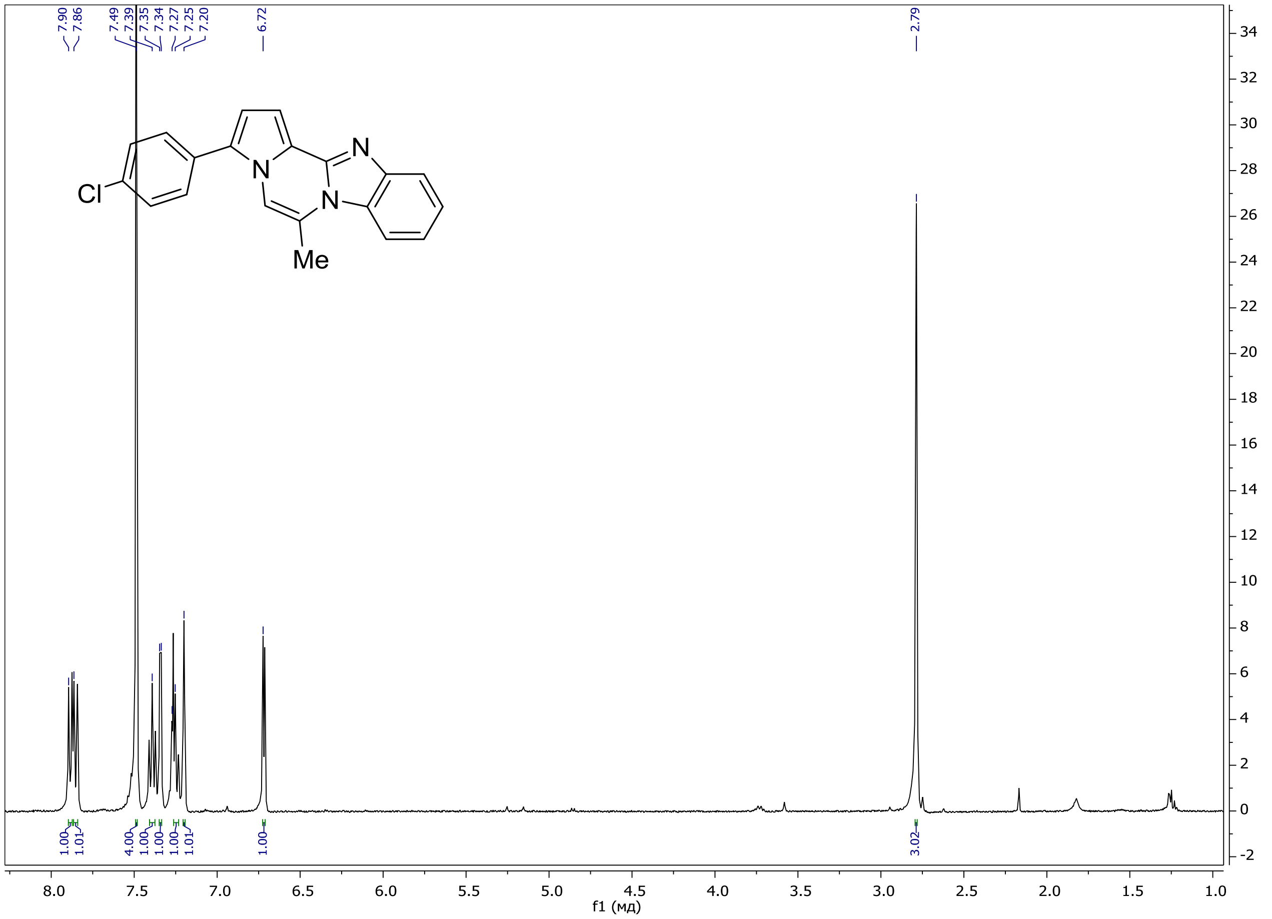

Figure S14. ^{13}C-NMR (100 MHz, $CDCl_3$) spectrum of **3f**

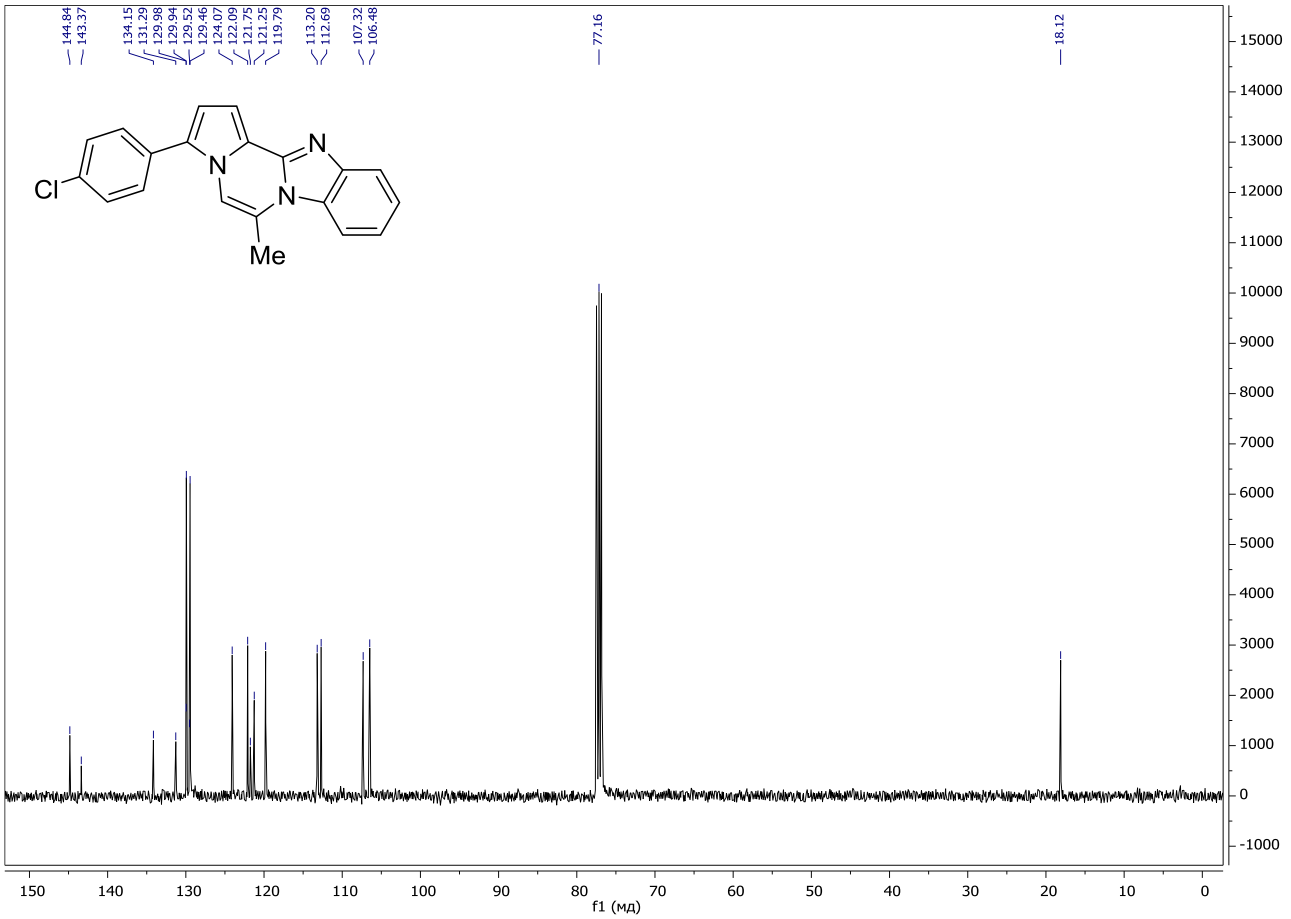

Figure S15. ^{1}H-NMR (400 MHz, $CDCl_3$) spectrum of **3g**

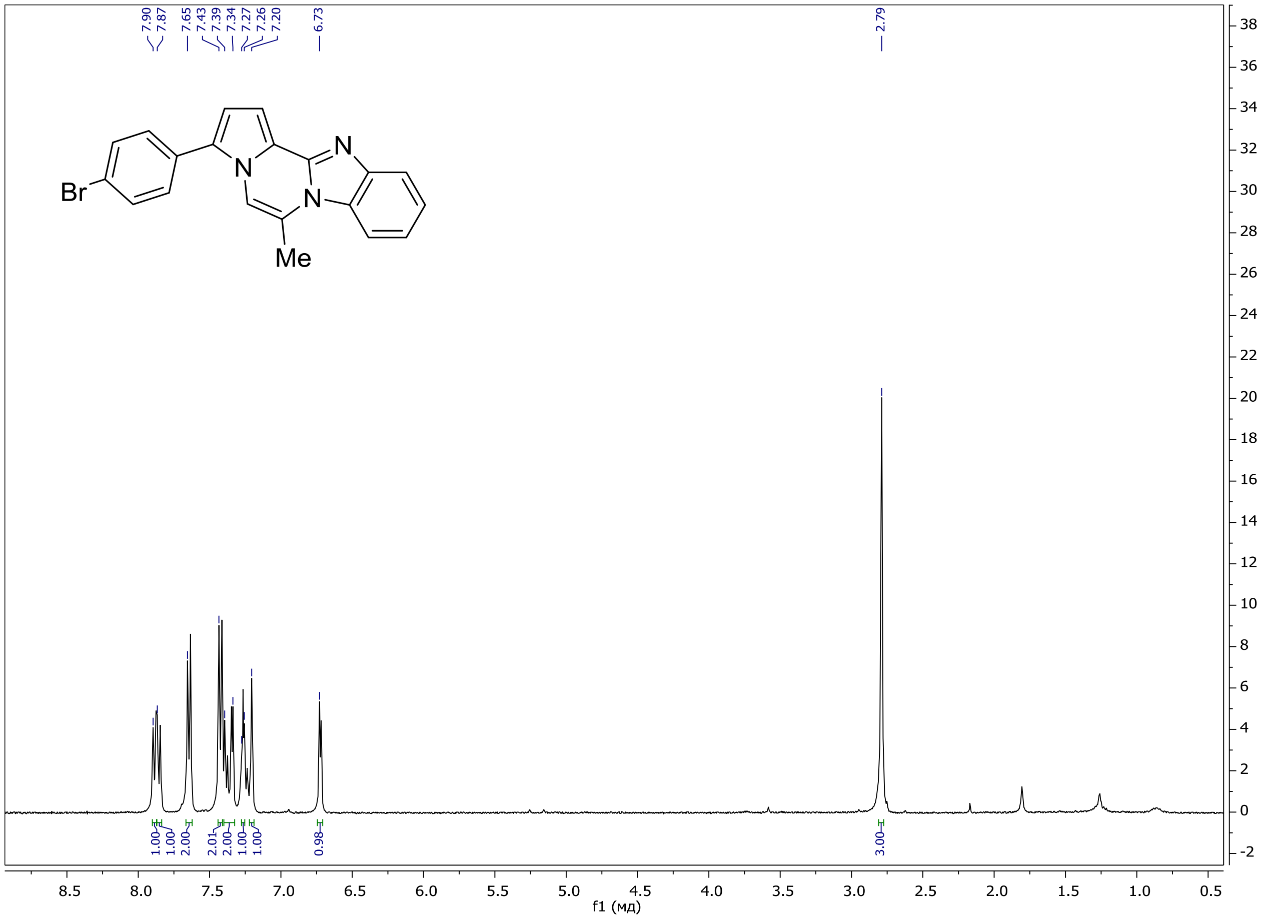

Figure S16. ^{13}C-NMR (100 MHz, $CDCl_3$) spectrum of **3g**

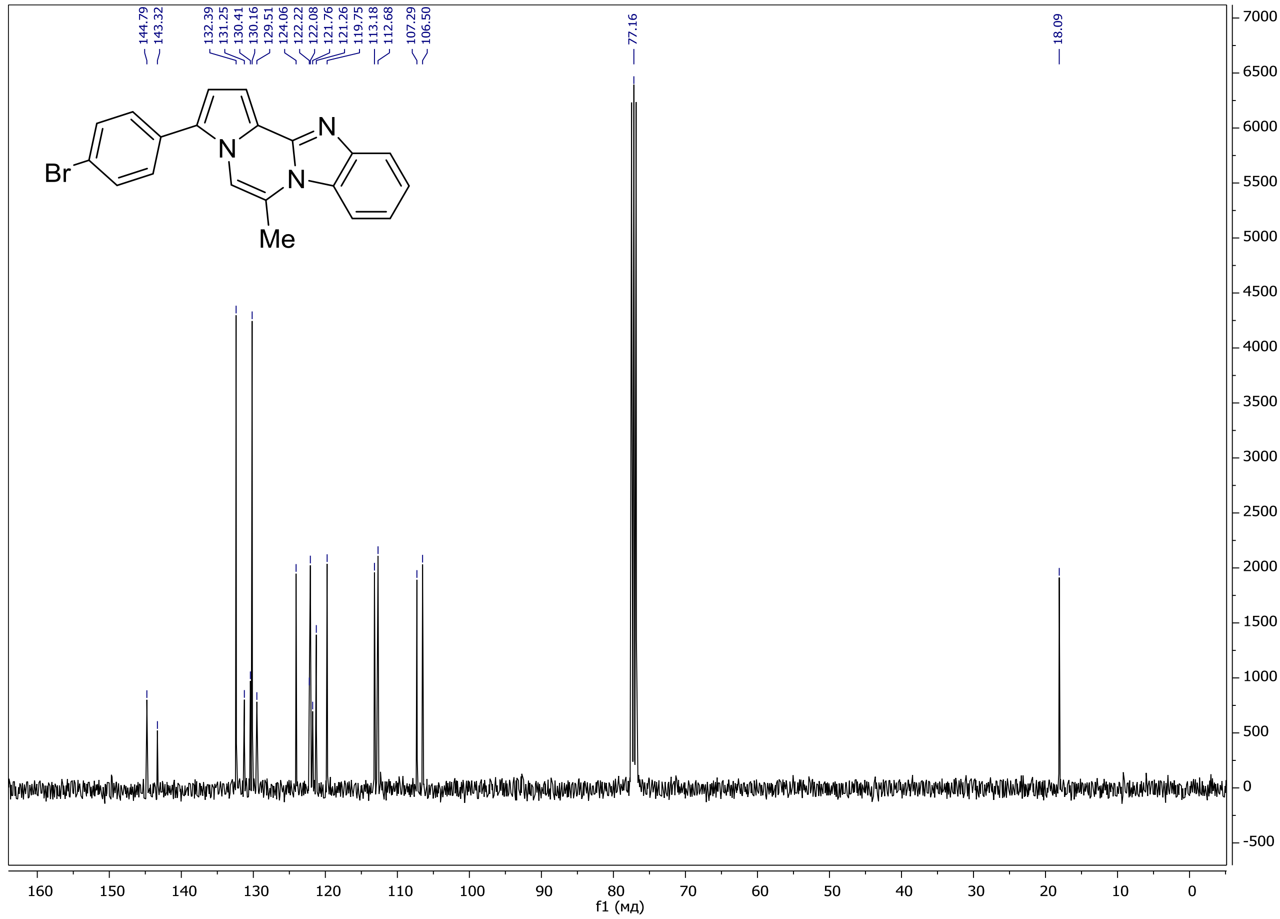

Figure S17. ^{1}H-NMR (400 MHz, $CDCl_3$) spectrum of **3h**

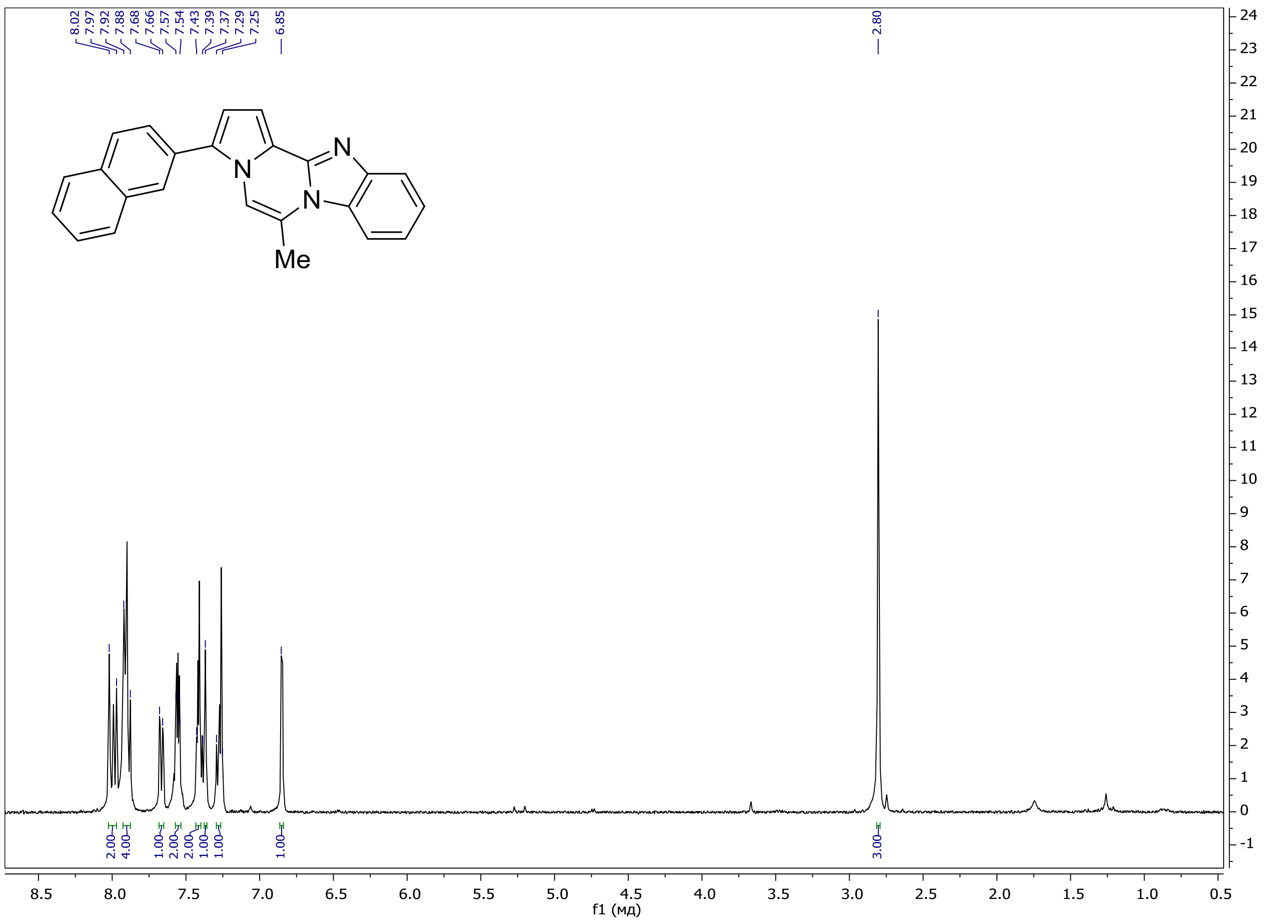

Figure S18. ^{13}C-NMR (100 MHz, $CDCl_3$) spectrum of **3h**

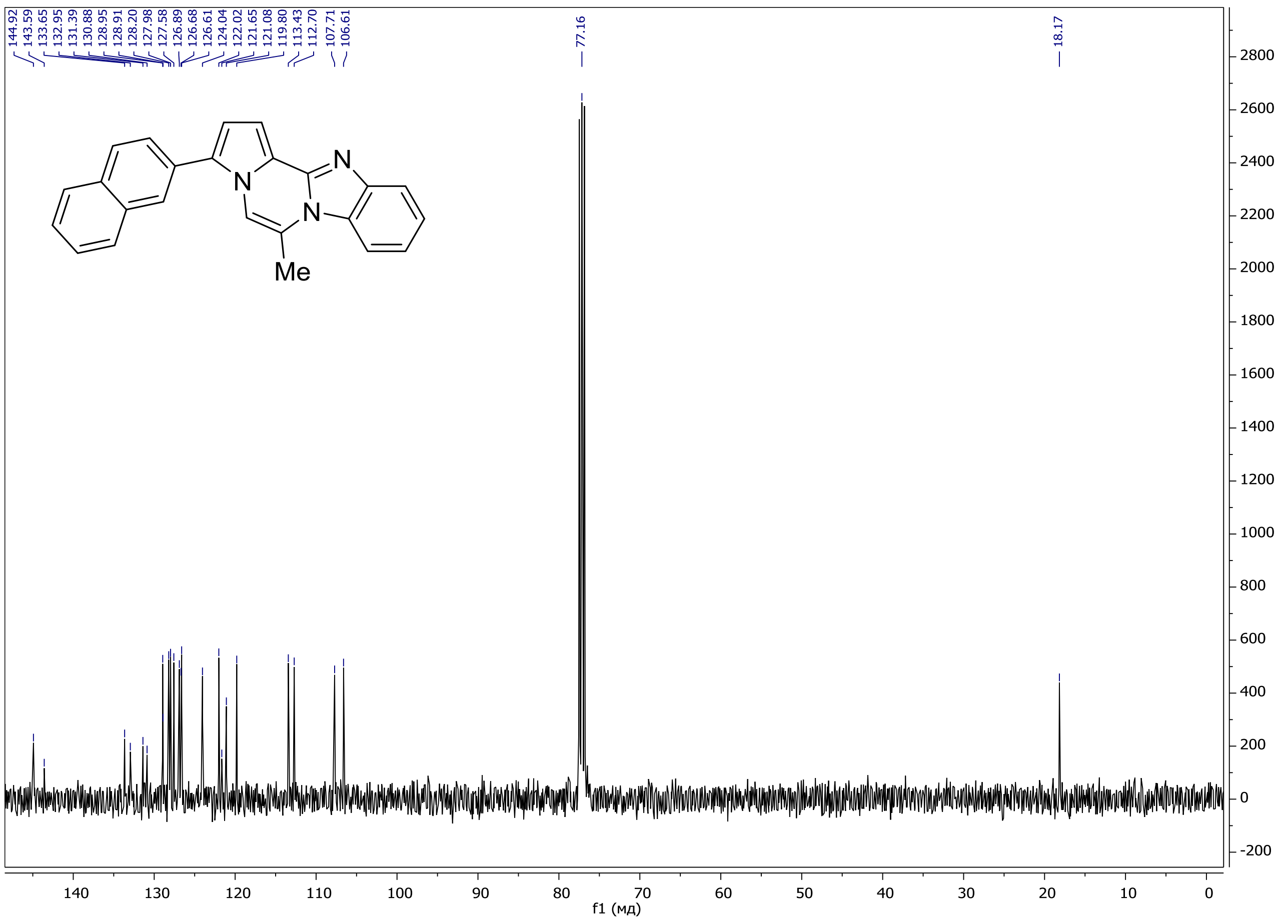

Figure S19. ^{1}H-NMR (400 MHz, $CDCl_3$) spectrum of **3i**

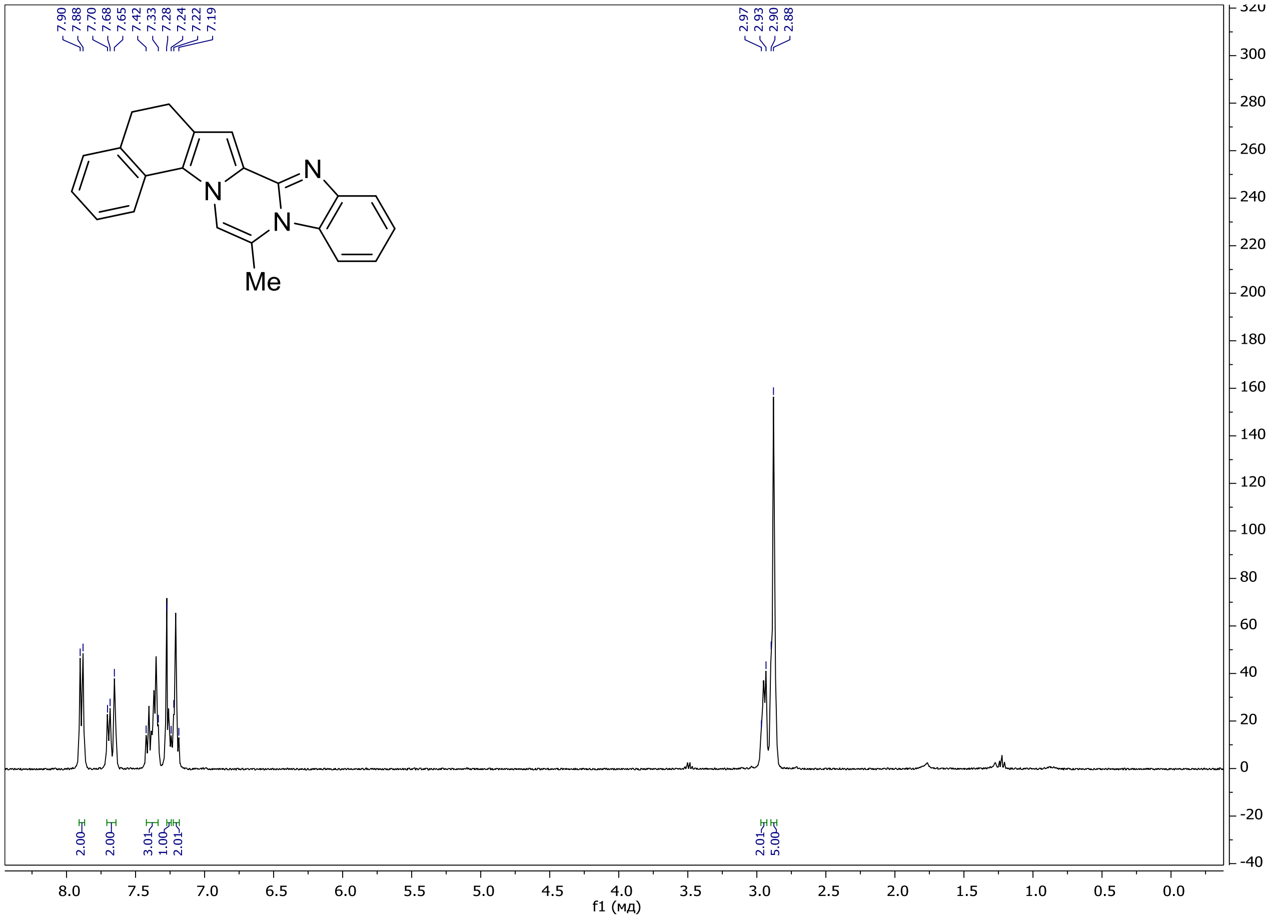

Figure S20. ^{13}C-NMR (100 MHz, $CDCl_3$) spectrum of **3i**

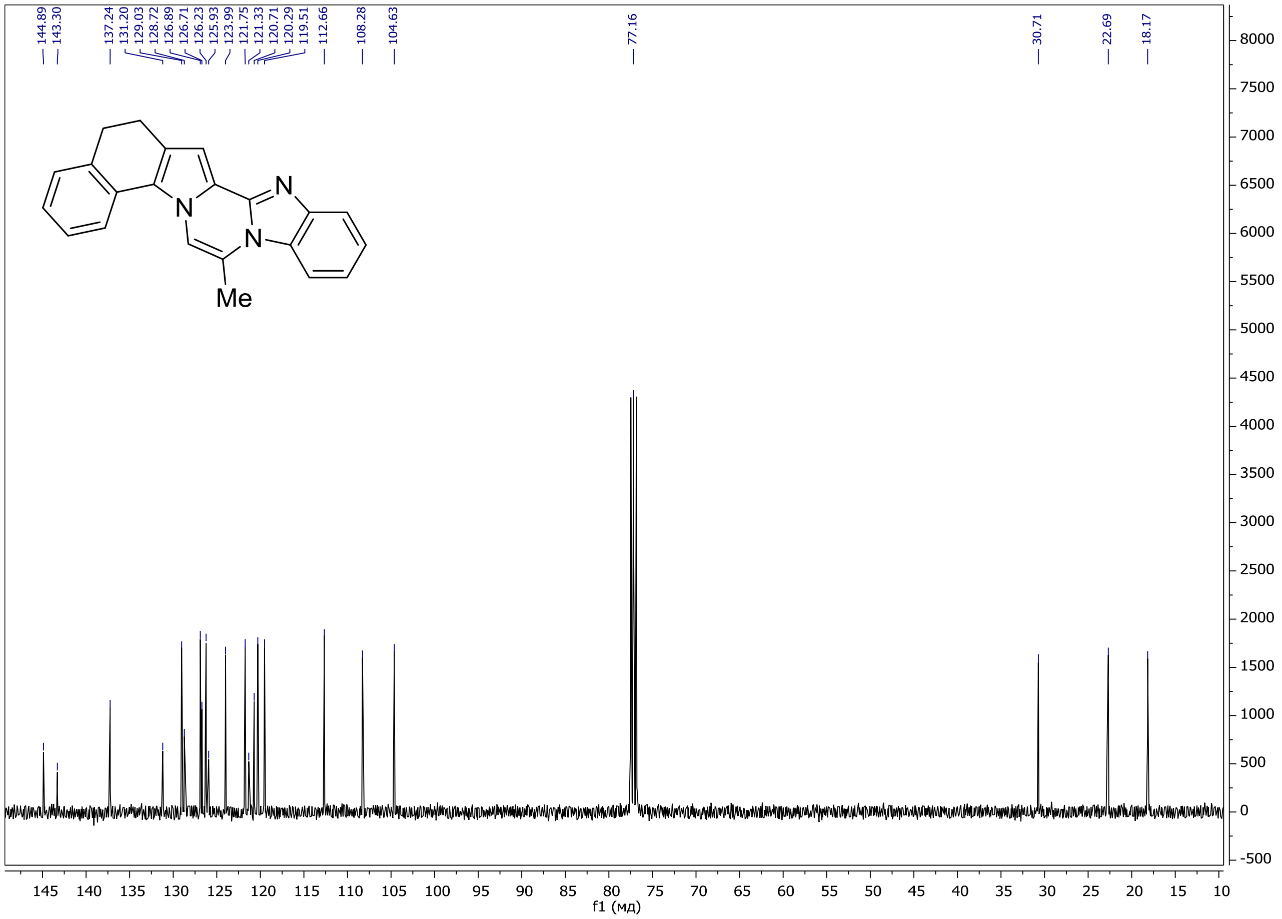

Figure S21. ^{1}H-NMR (400 MHz, $CDCl_3$) spectrum of **3j**

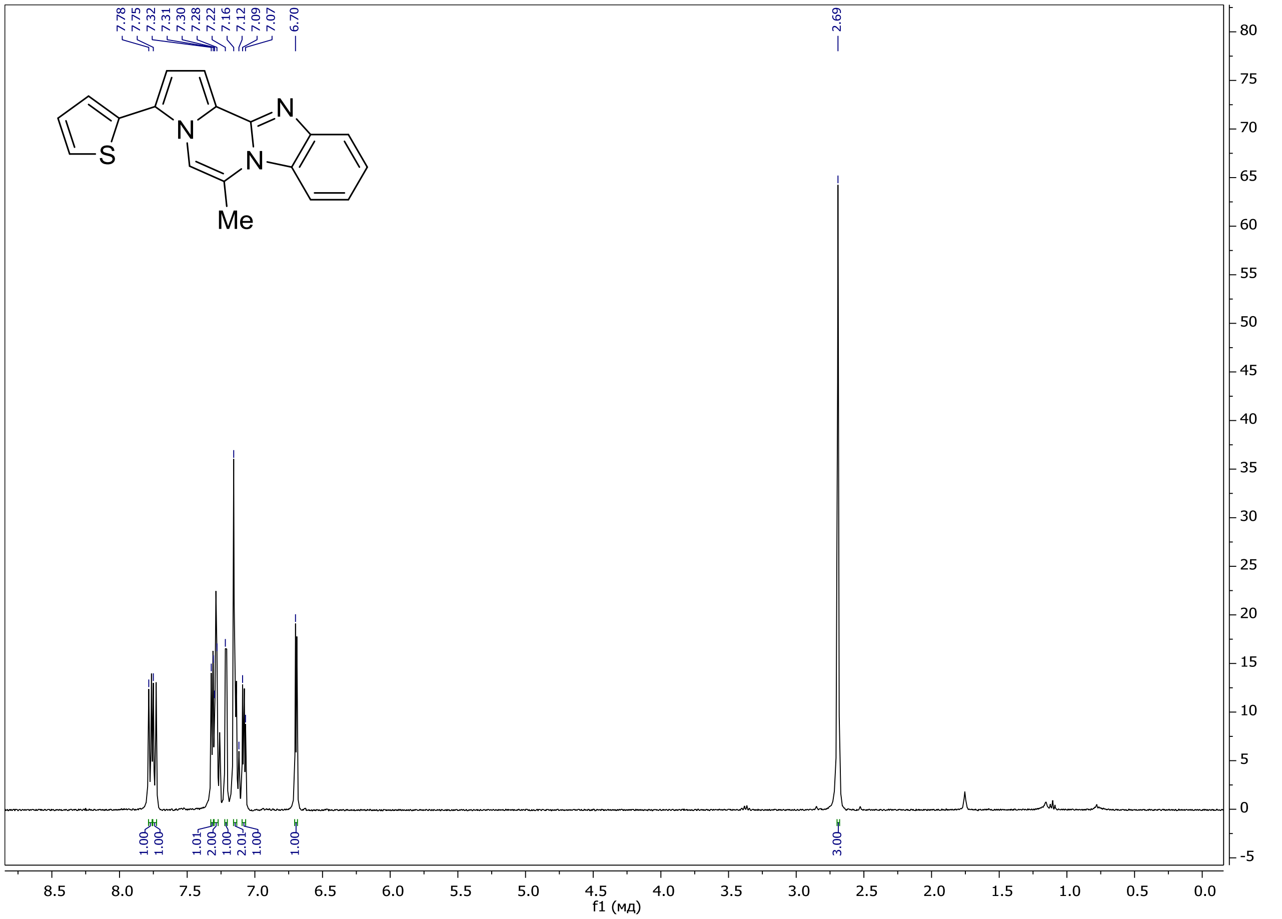

Figure S22. ^{13}C-NMR (100 MHz, $CDCl_3$) spectrum of **3j**

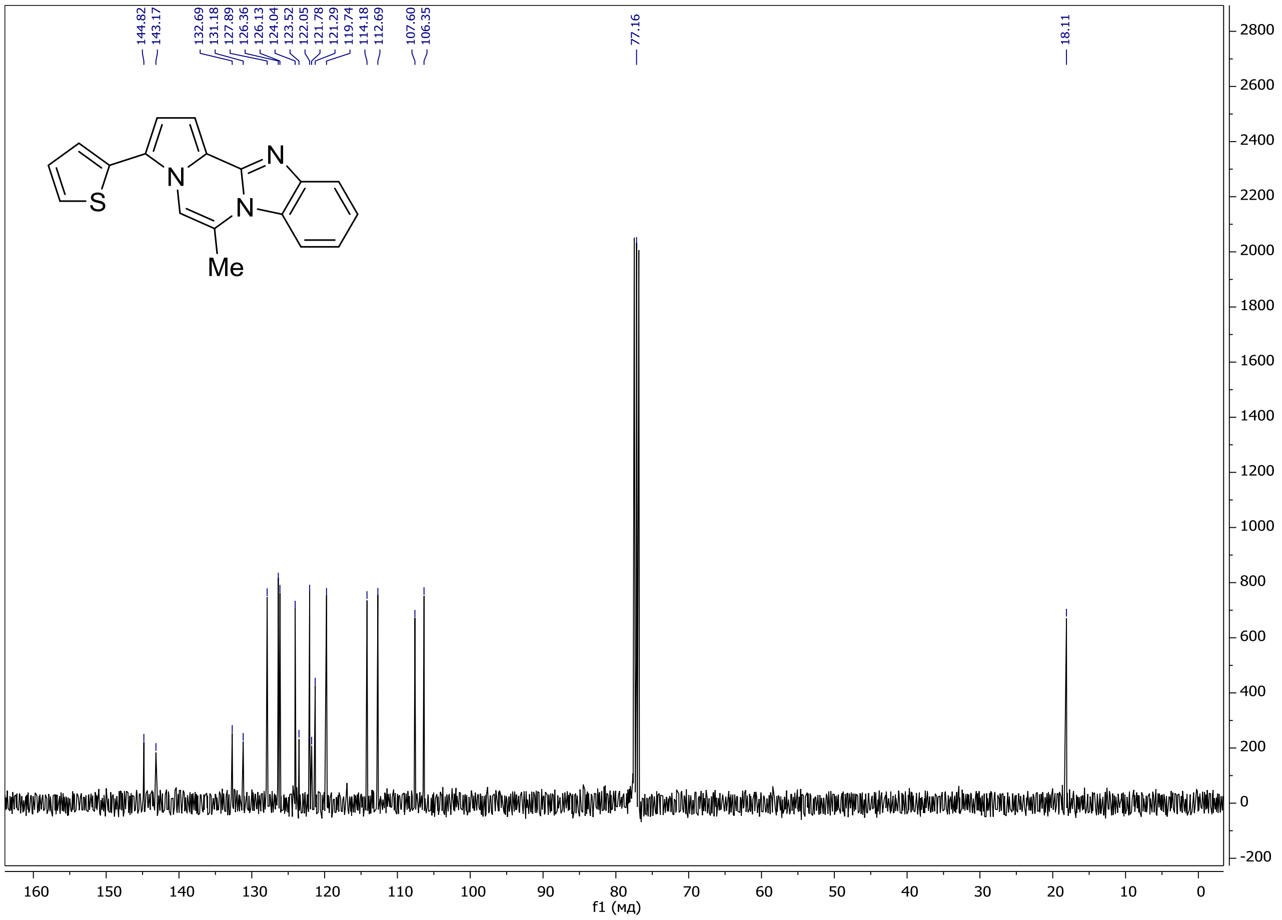

3. NMR spectra of the obtained compounds 4

Figure S23. ^{1}H-NMR (400 MHz, $CDCl_3$) spectrum of **4a**

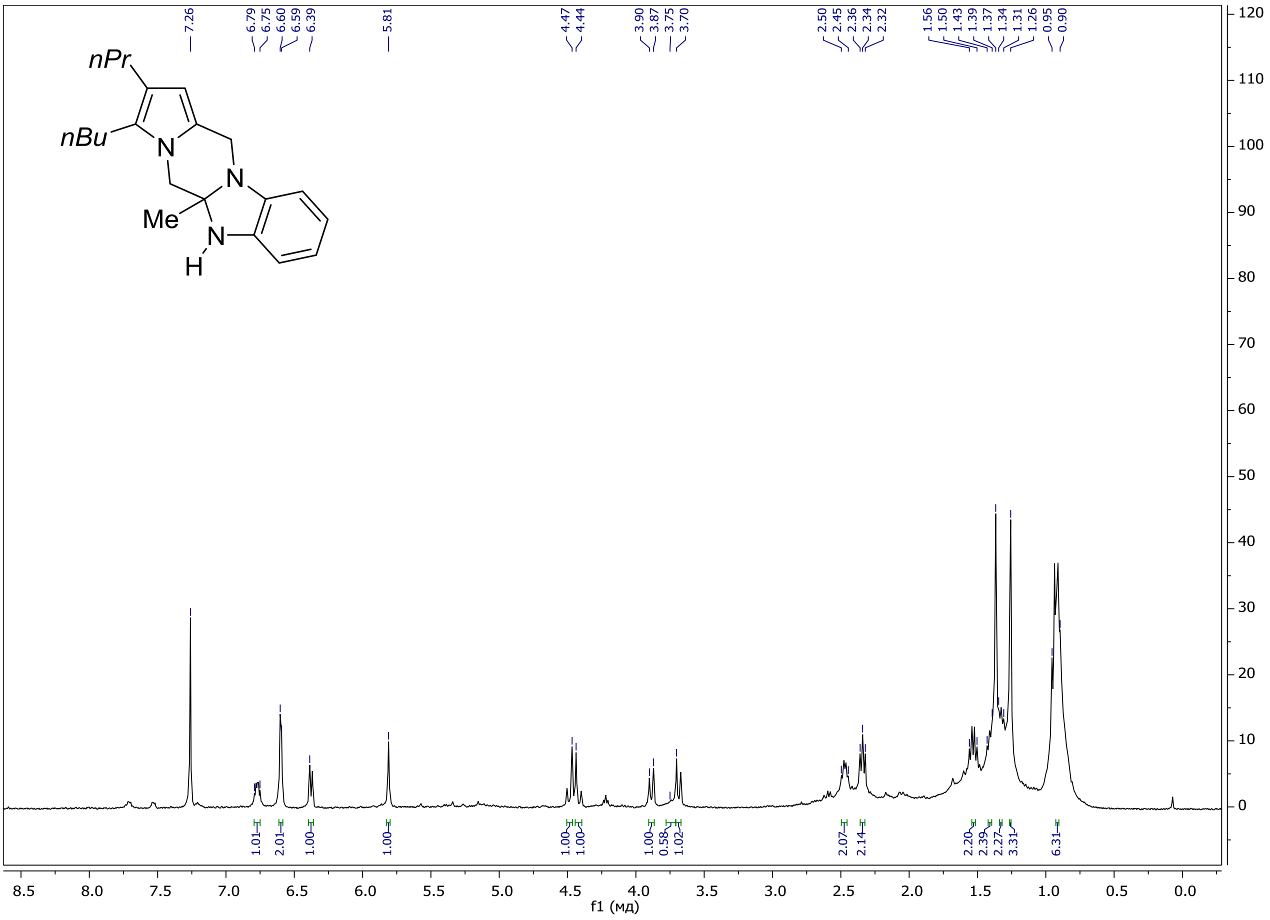

Figure S24. ^{1}H-NMR (400 MHz, $CDCl_3$) spectrum of **4b**

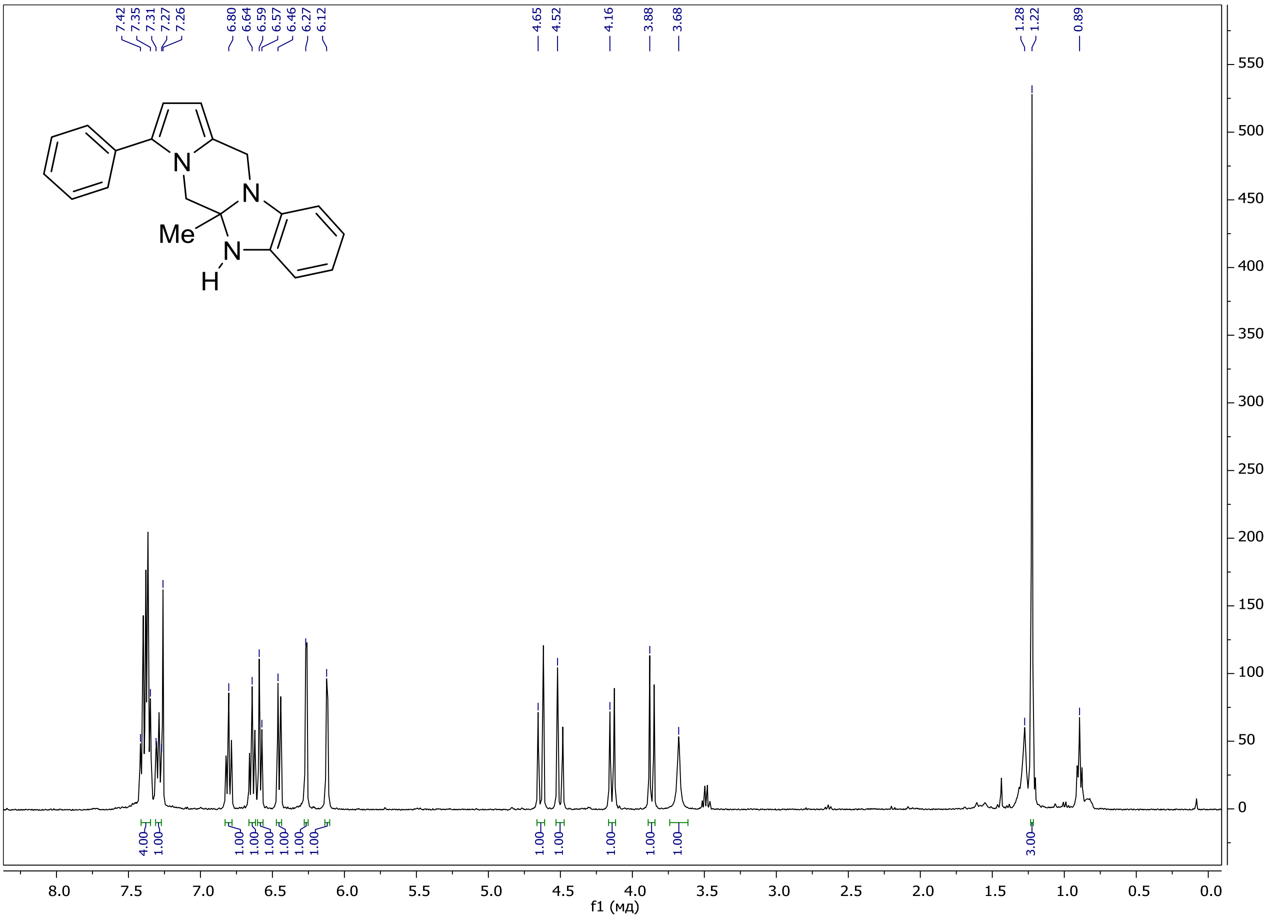

Figure S25. ^{13}C-NMR (100 MHz, $CDCl_3$) spectrum of **4b**

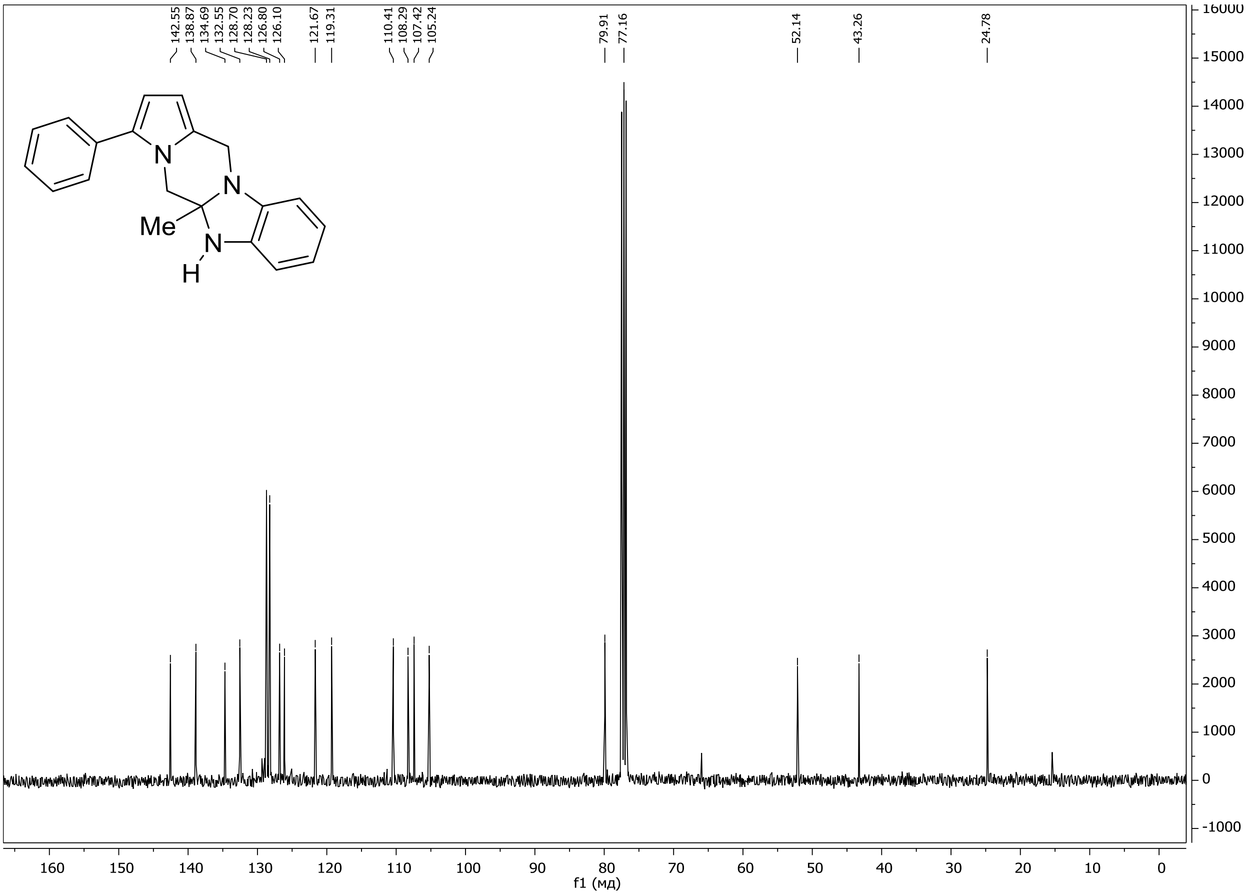

Figure S26. ^{1}H-NMR (400 MHz, $CDCl_3$) spectrum of **4c**

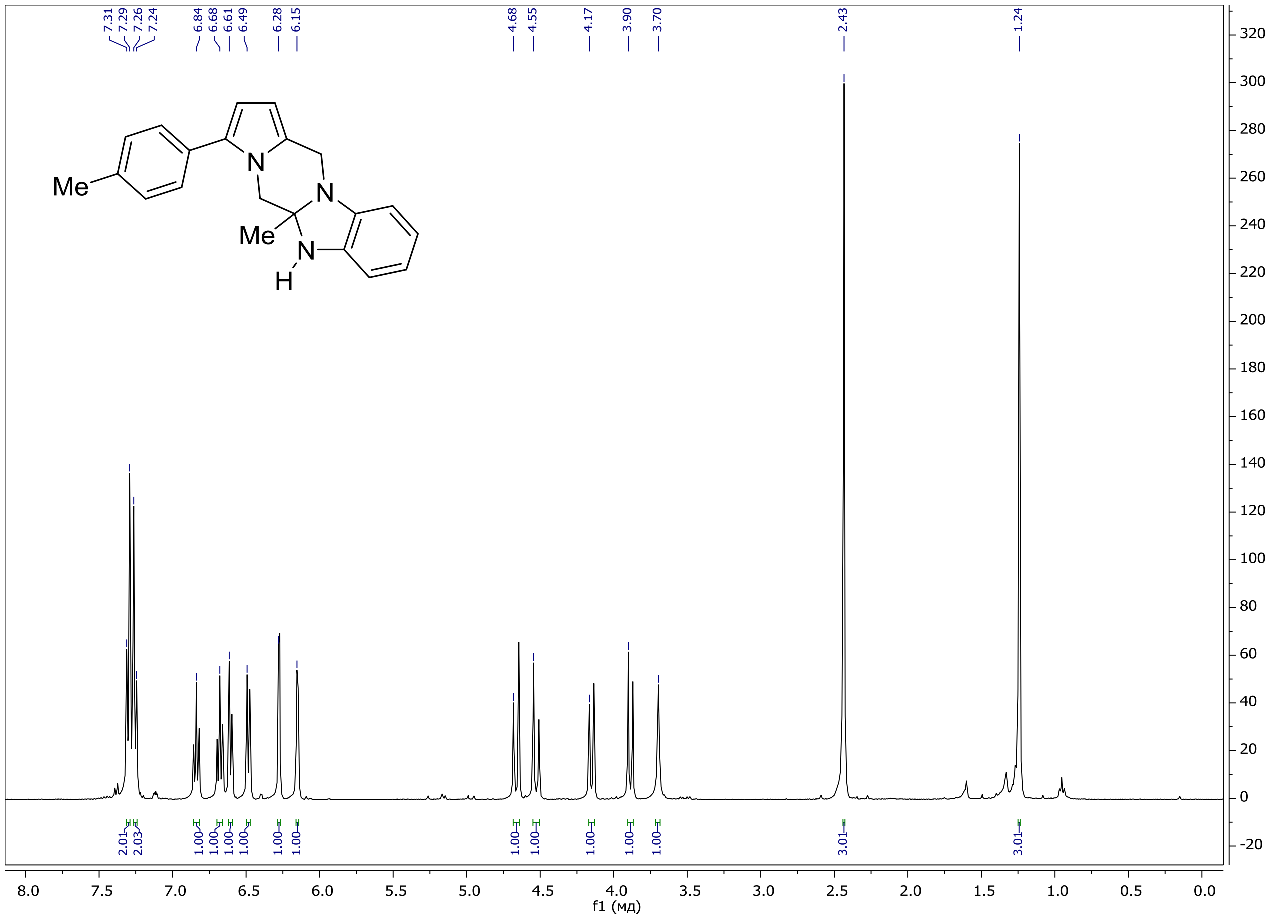

Figure S27. ^{13}C-NMR (100 MHz, $CDCl_3$) spectrum of **4c**

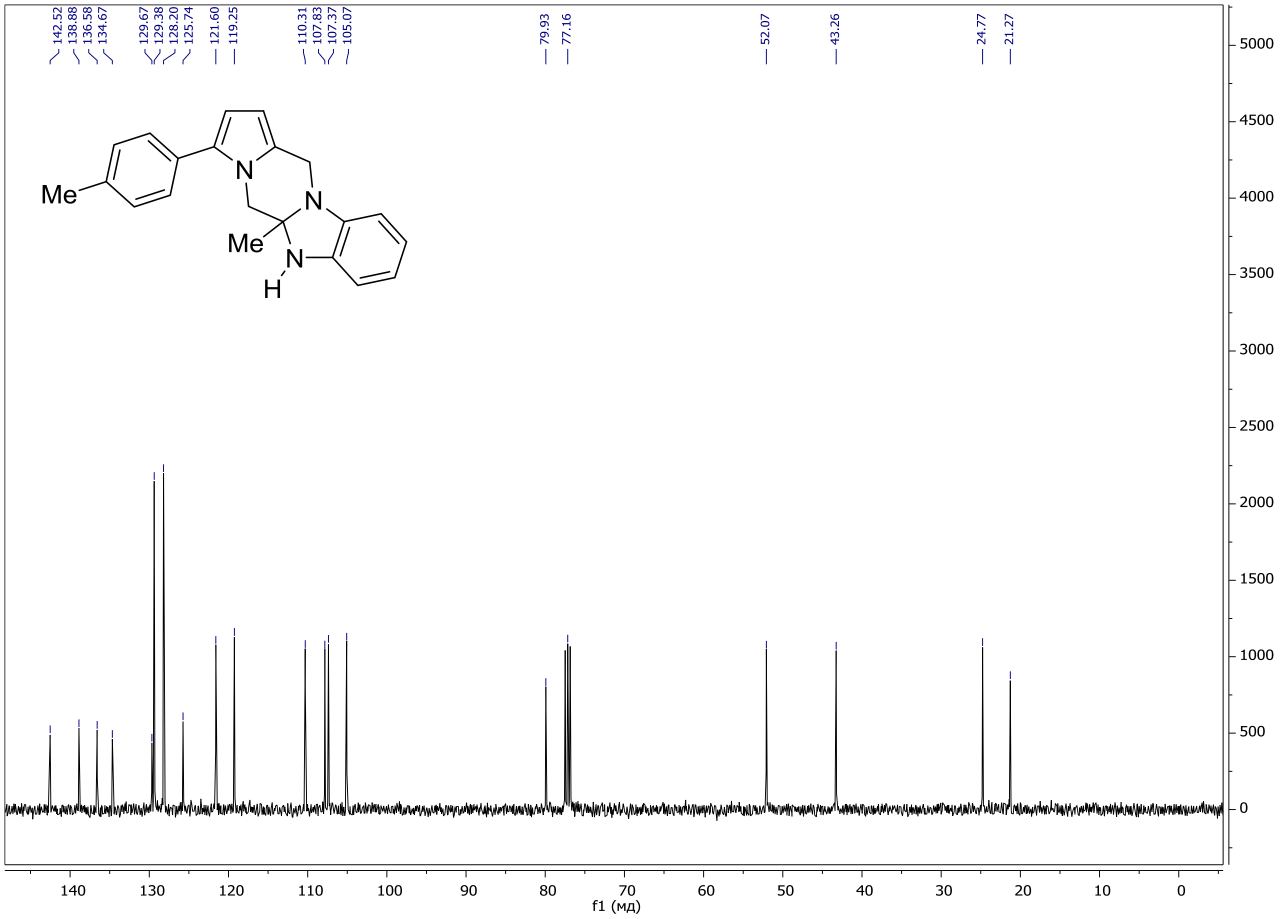

Figure S28. ^{1}H-NMR (400 MHz, $CDCl_3$) spectrum of **4d**

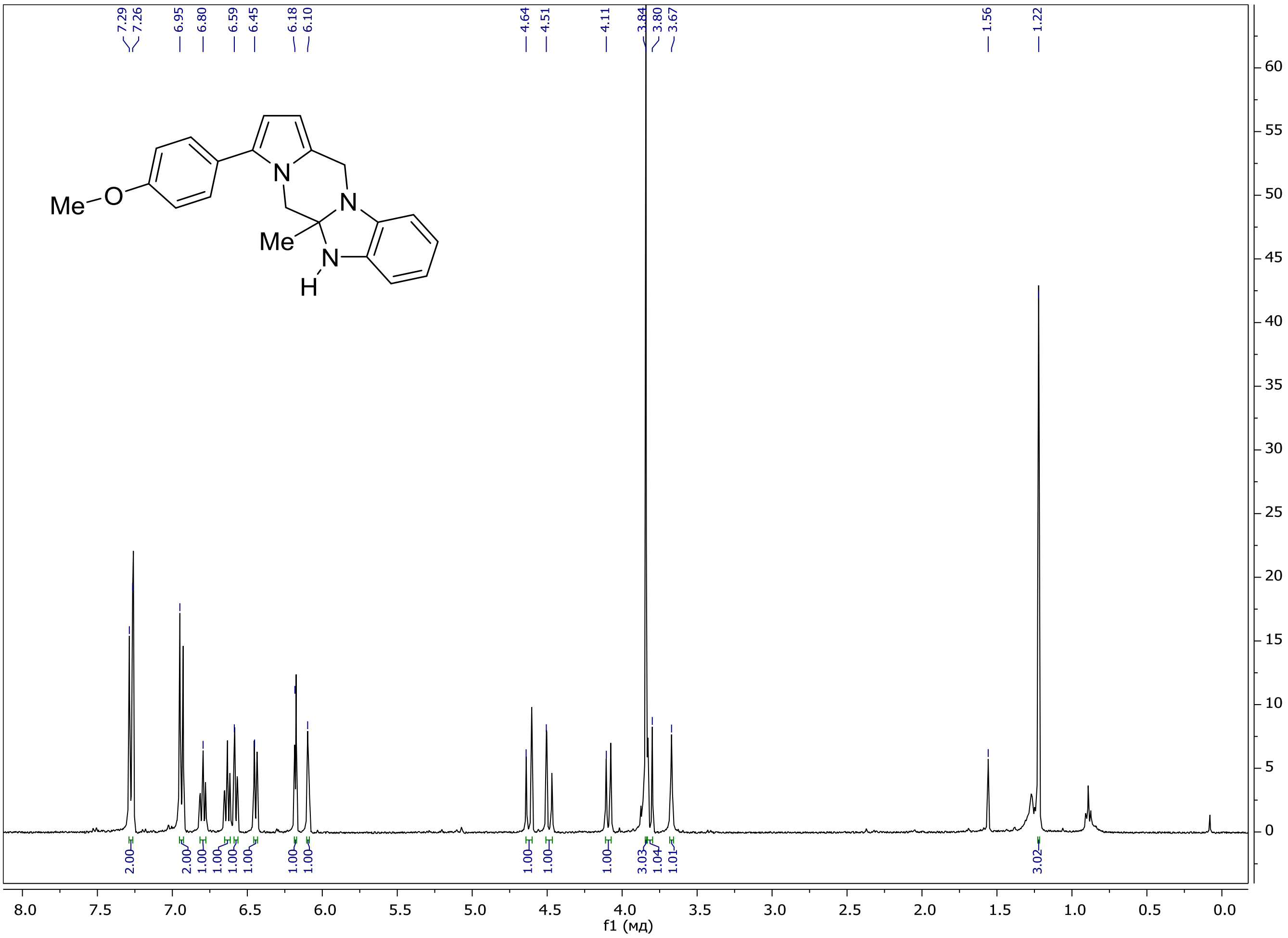

Figure S29. ^{13}C-NMR (100 MHz, $CDCl_3$) spectrum of **4d**

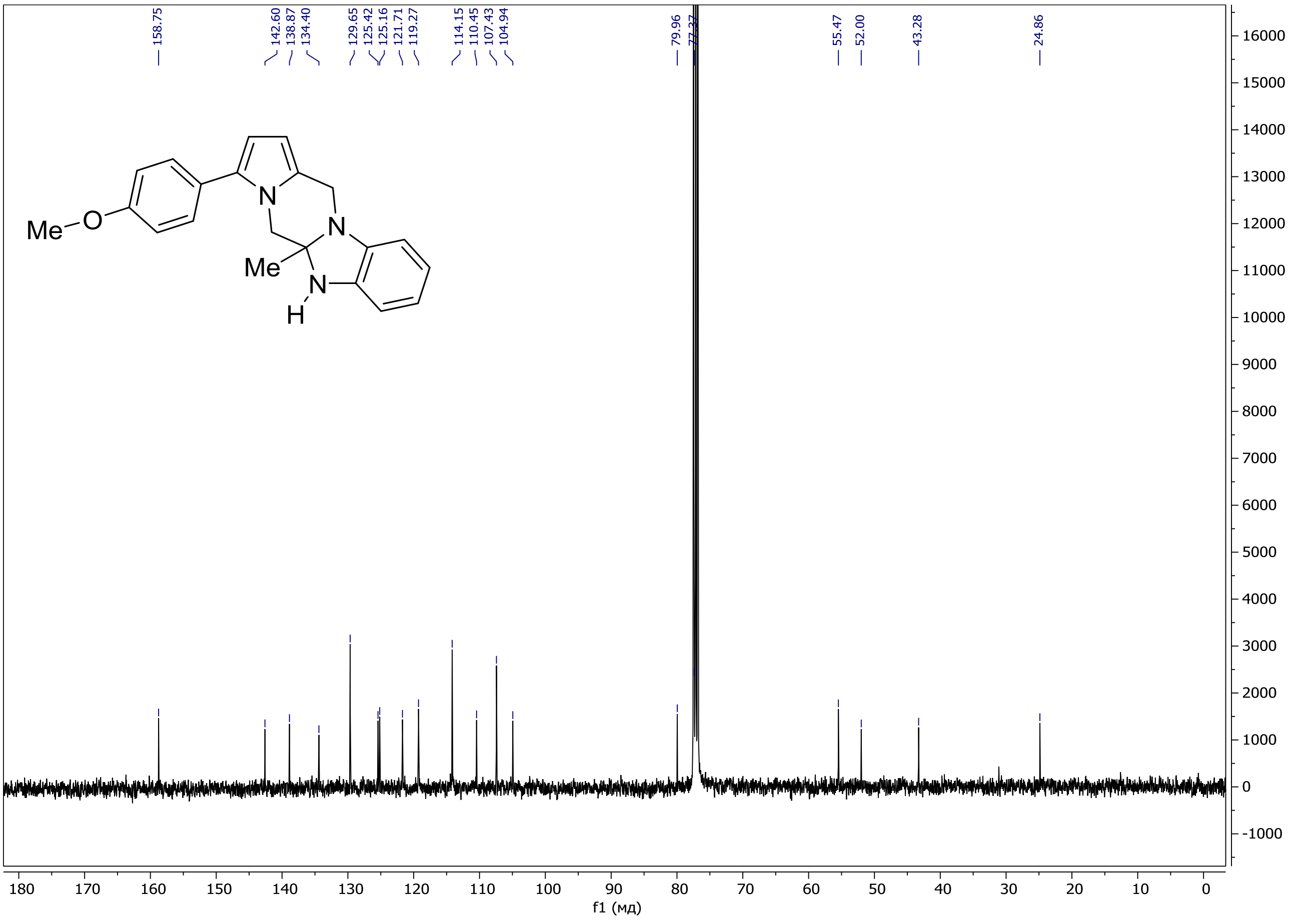

Figure S30. ^{1}H-NMR (400 MHz, $CDCl_3$) spectrum of **4e**

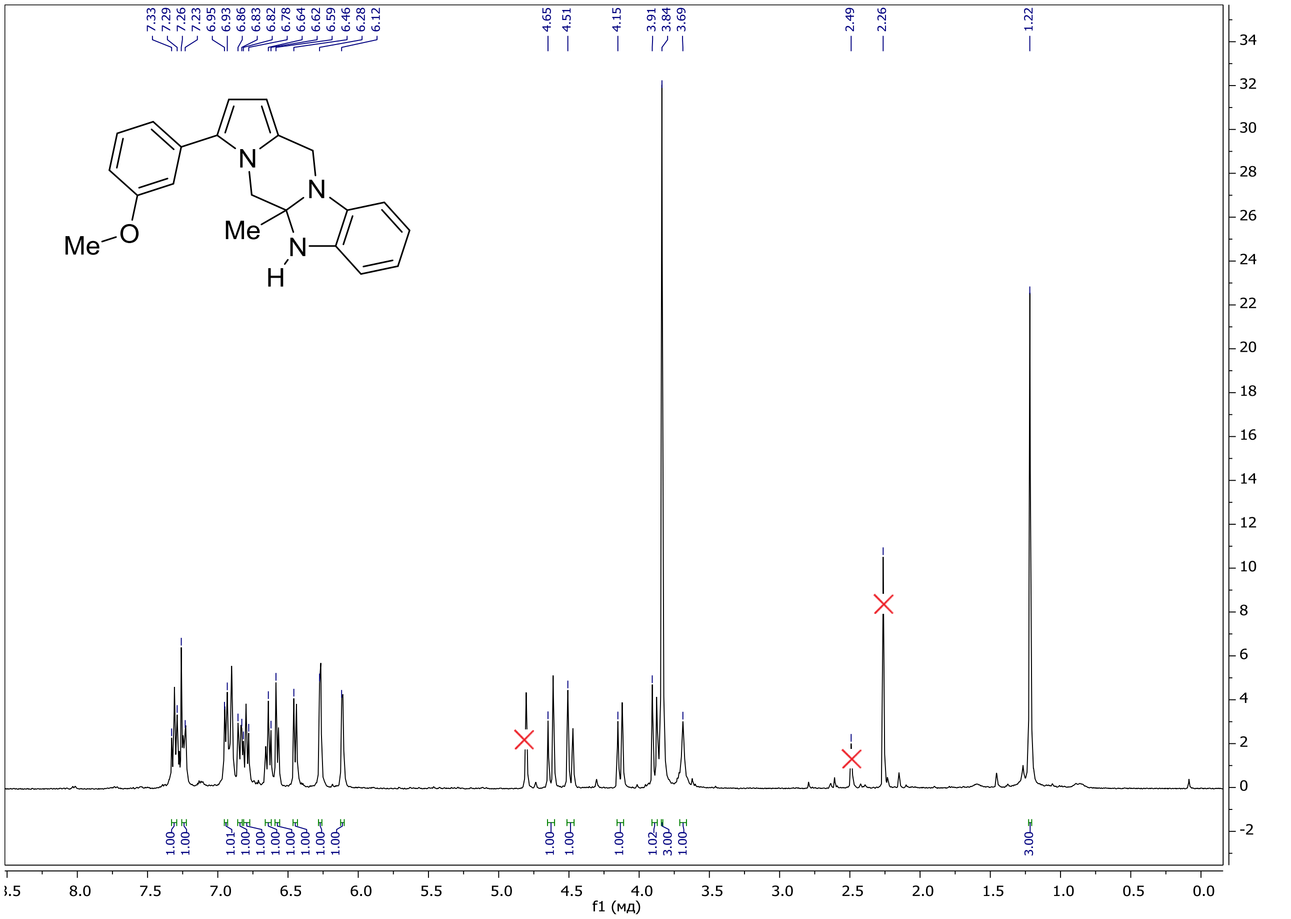

Figure S31. ^{13}C-NMR (100 MHz, $CDCl_3$) spectrum of **4e**

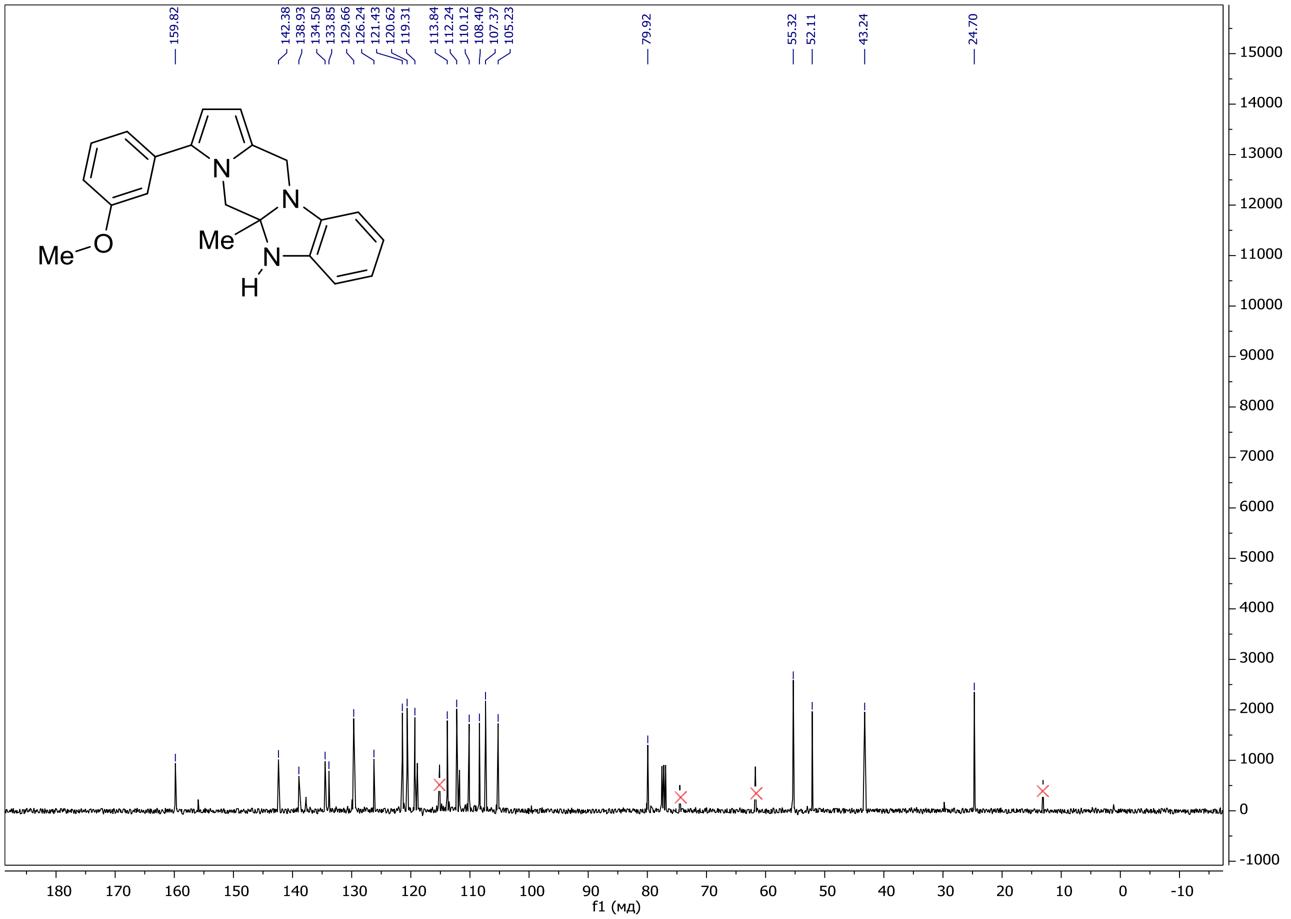

Figure S32. ^{1}H-NMR (400 MHz, $CDCl_3$) spectrum of **4f**

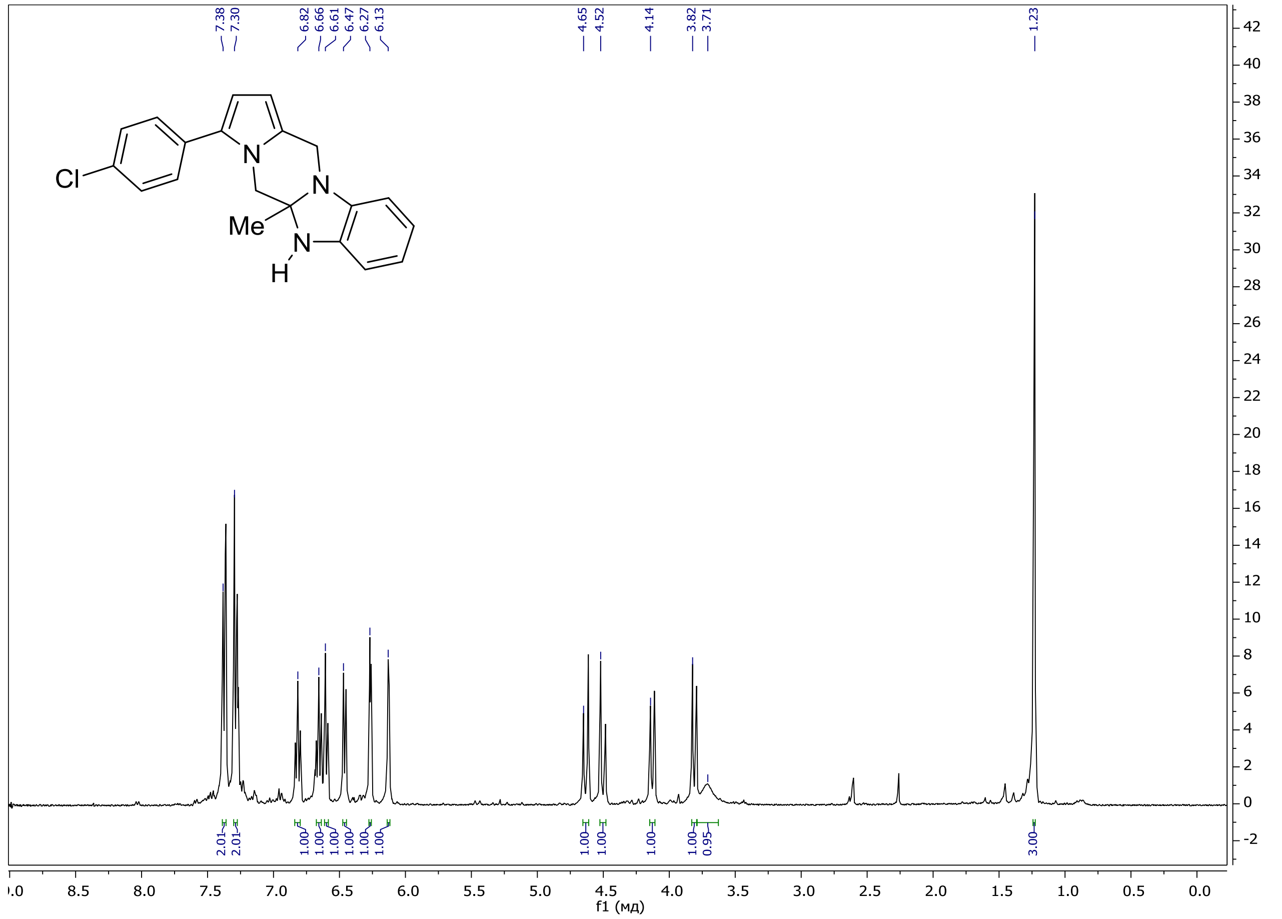

Figure S33. ^{13}C-NMR (100 MHz, $CDCl_3$) spectrum of **4f**

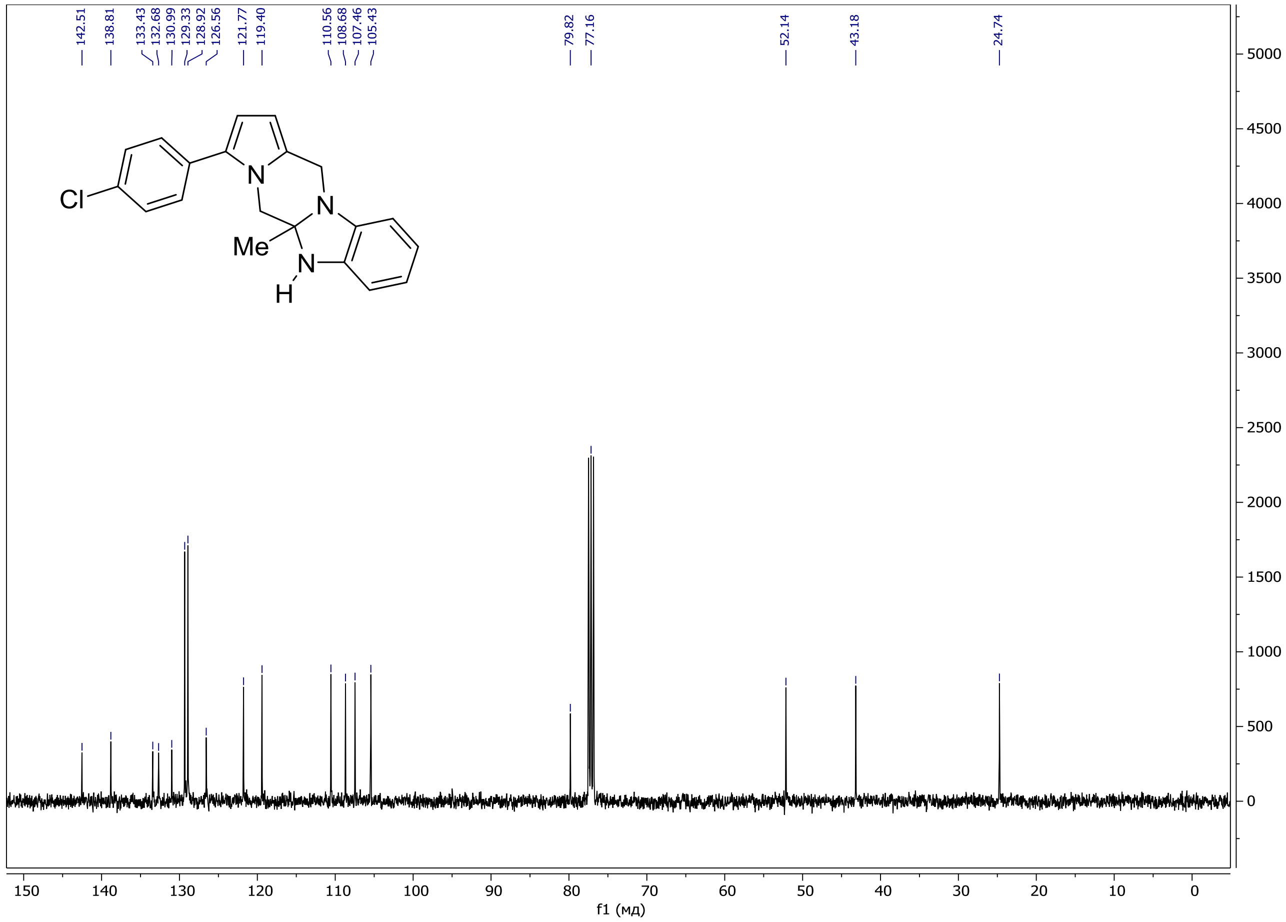

Figure S34. ^{1}H-NMR (400 MHz, $CDCl_3$) spectrum of **4g**

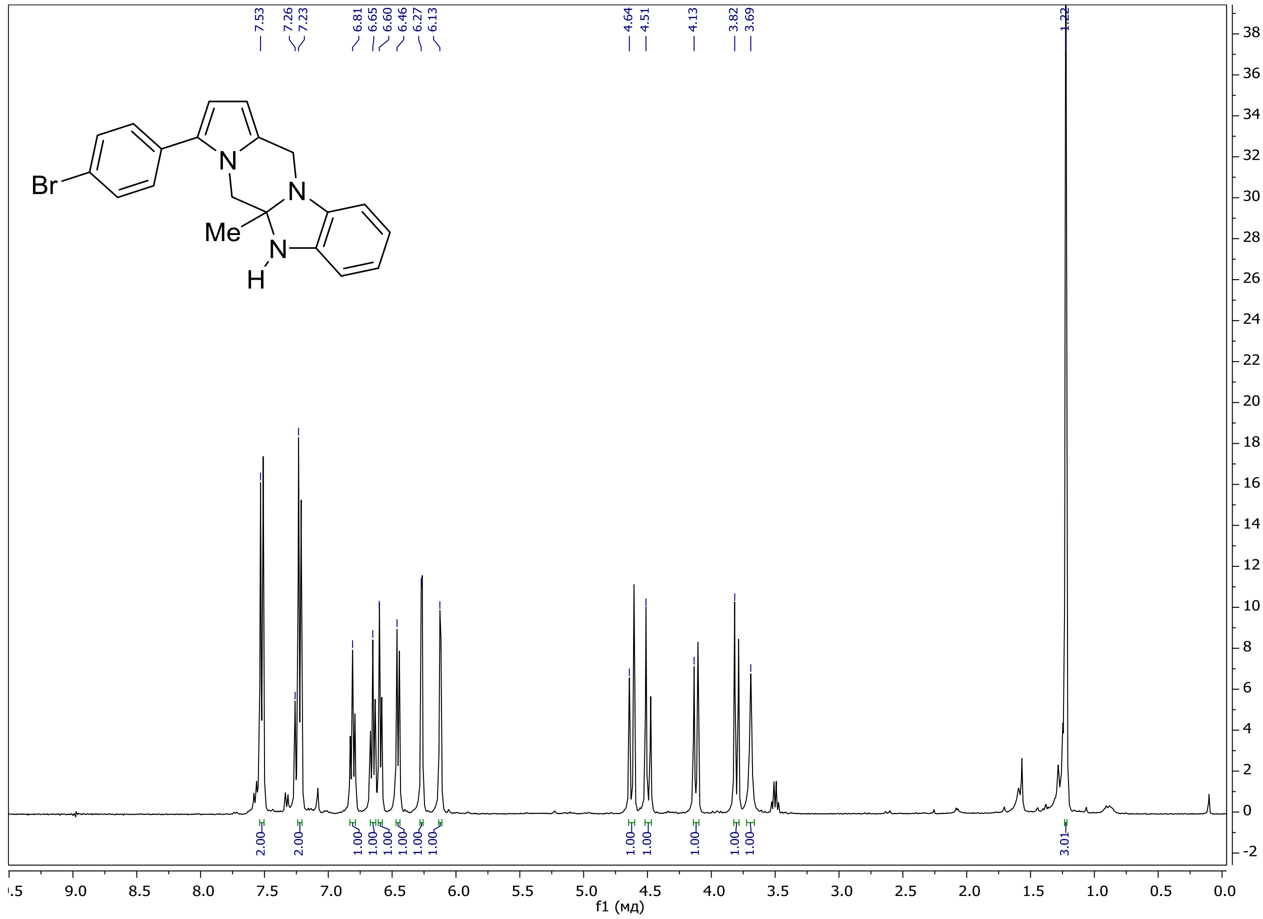

Figure S35. ^{13}C-NMR (100 MHz, $CDCl_3$) spectrum of **4g**

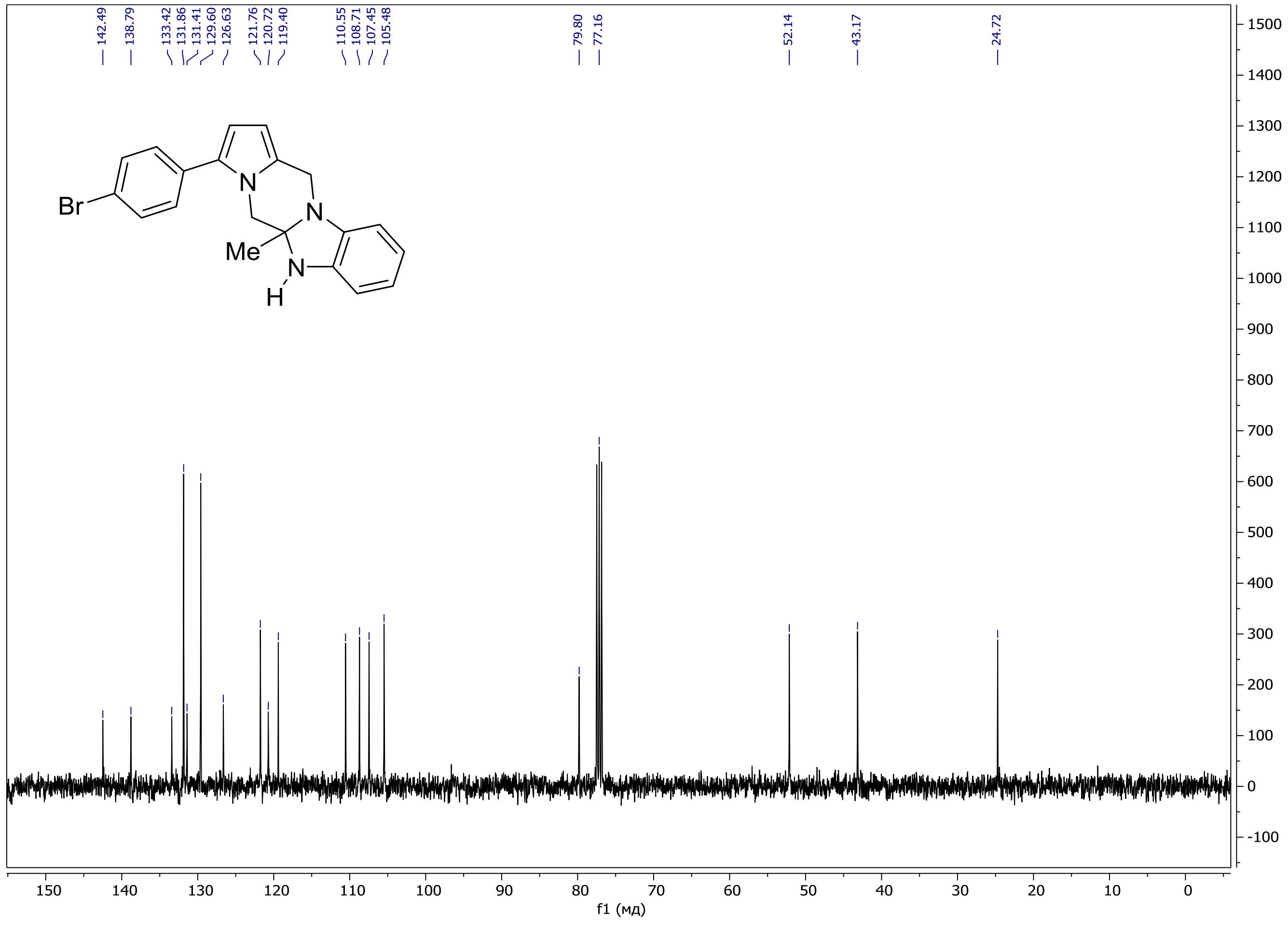

Figure S36. ^{1}H-NMR (400 MHz, $CDCl_3$) spectrum of **4h**

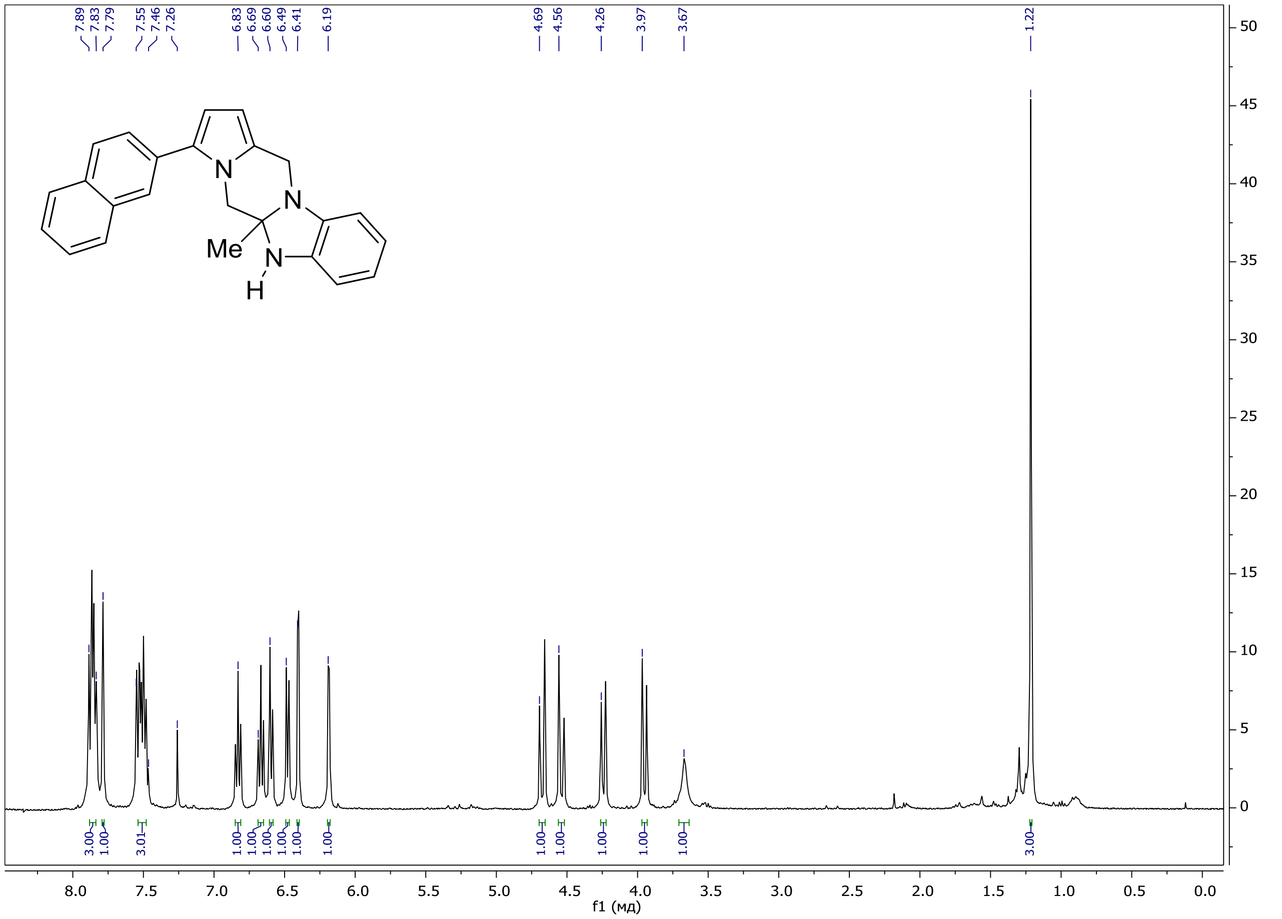

Figure S37. ^{13}C-NMR (100 MHz, $CDCl_3$) spectrum of **4h**

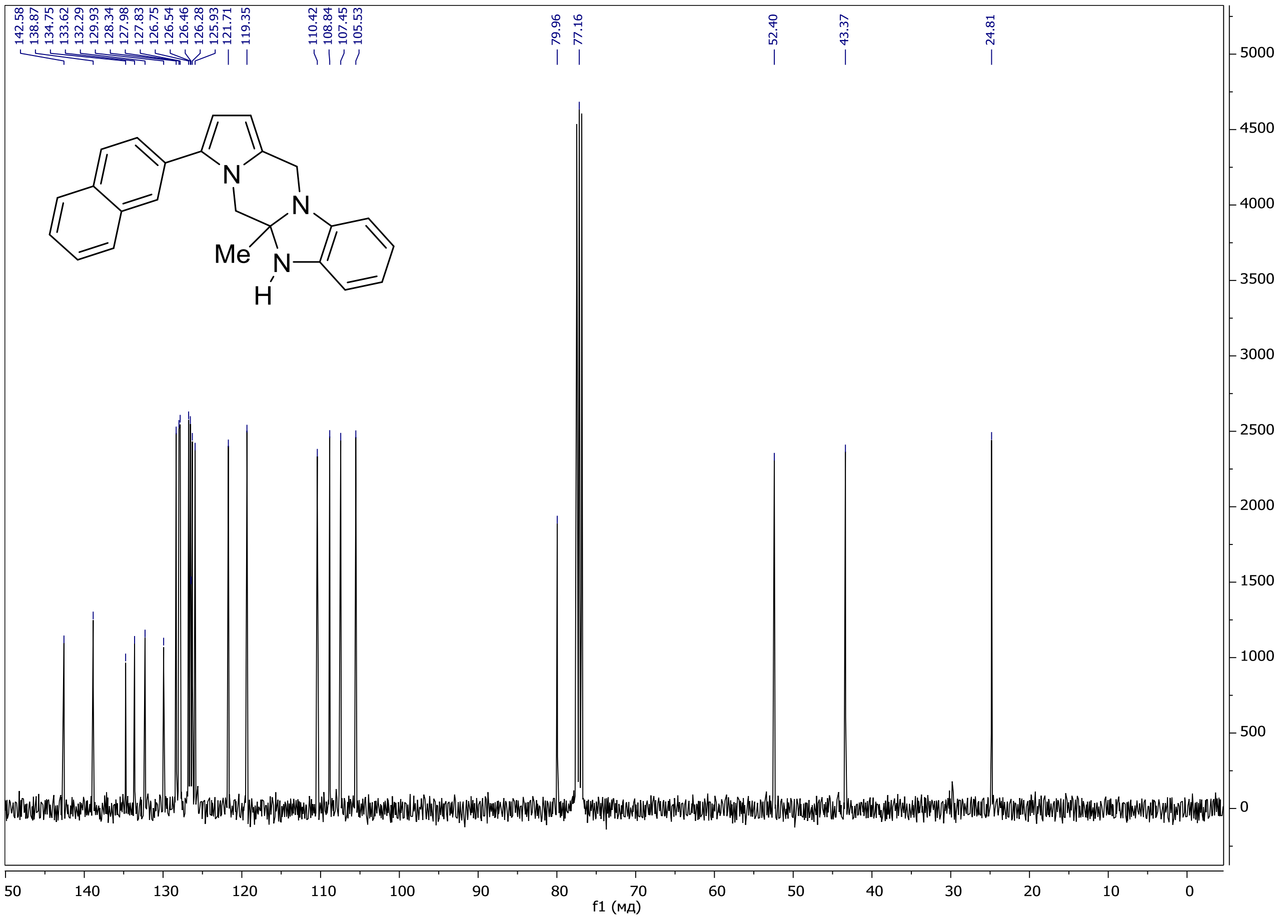

Figure S38. ^{1}H-NMR (400 MHz, $CDCl_3$) spectrum of **4i**

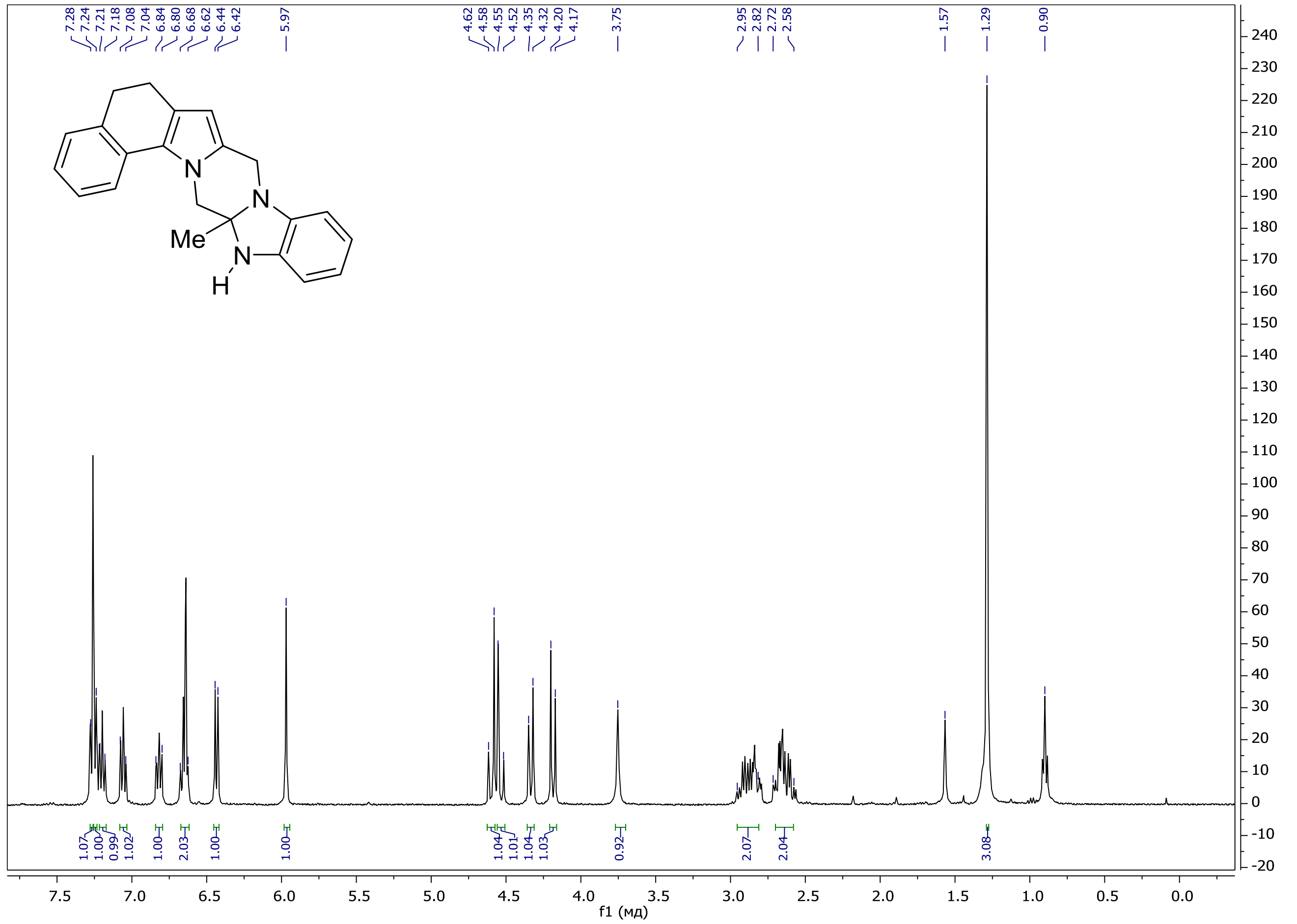

Figure S39. ^{13}C-NMR (100 MHz, $CDCl_3$) spectrum of **4i**

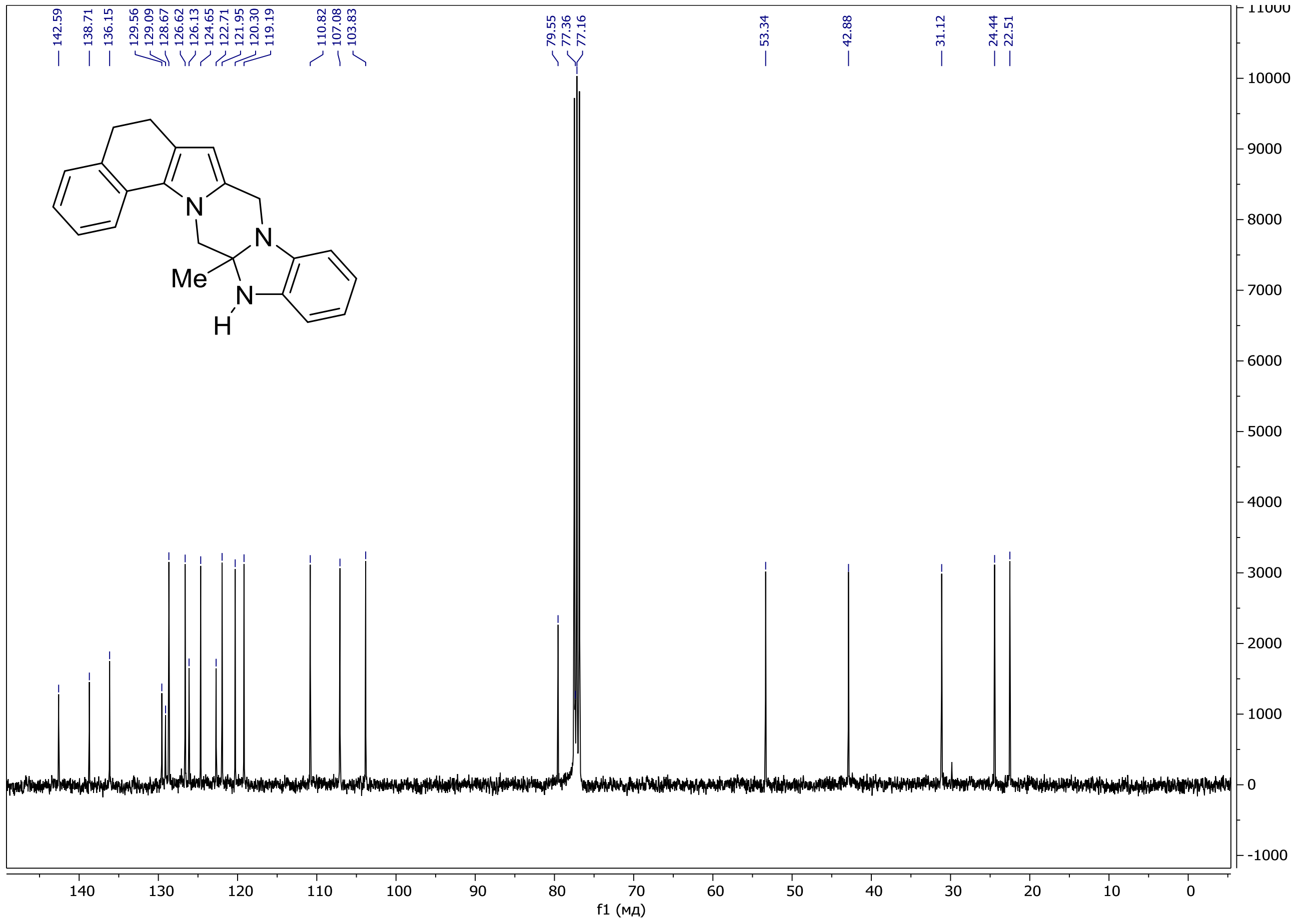

Figure S40. ^{1}H-NMR (400 MHz, $CDCl_3$) spectrum of **4j**

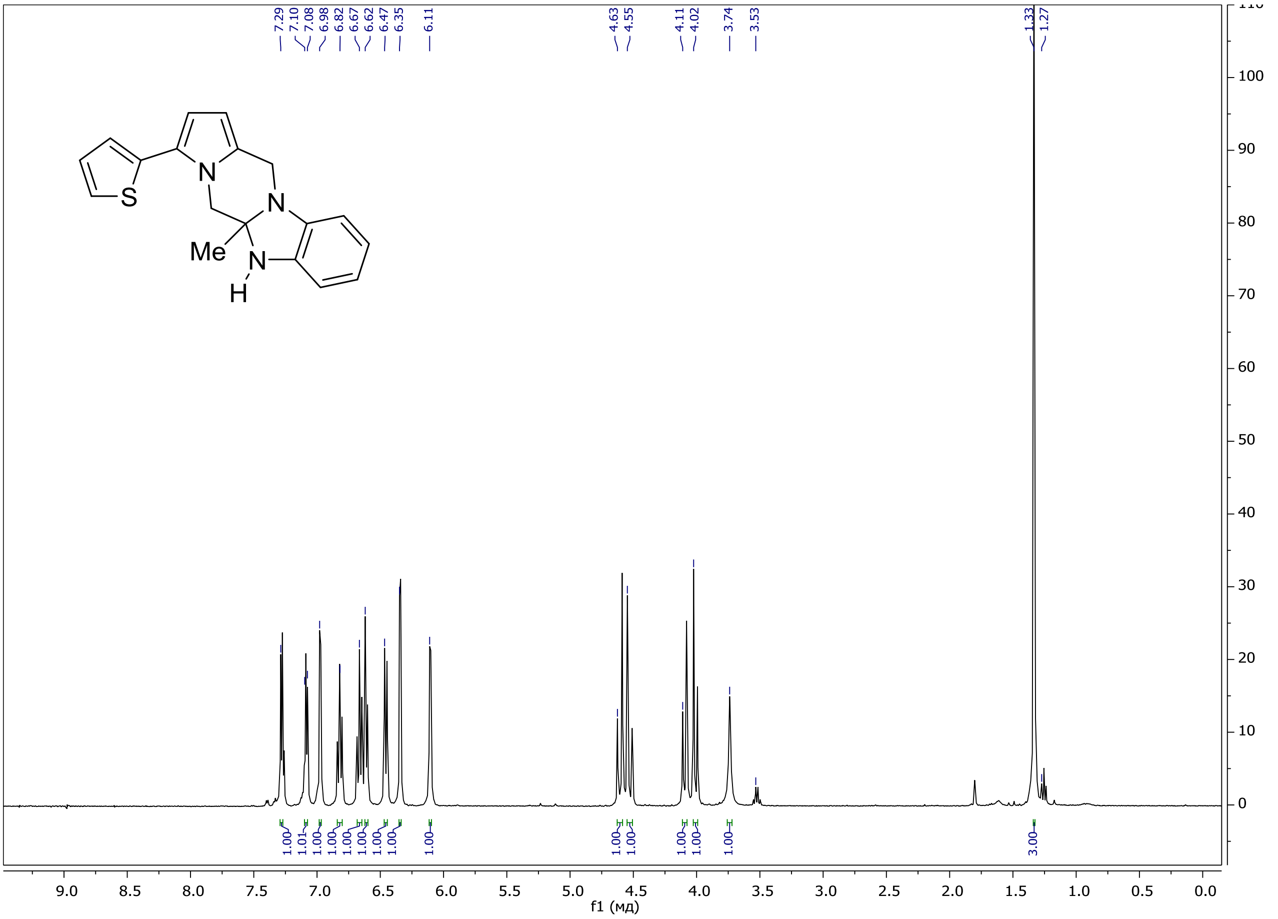

Figure S41. ^{13}C-NMR (100 MHz, $CDCl_3$) spectrum of **4j**

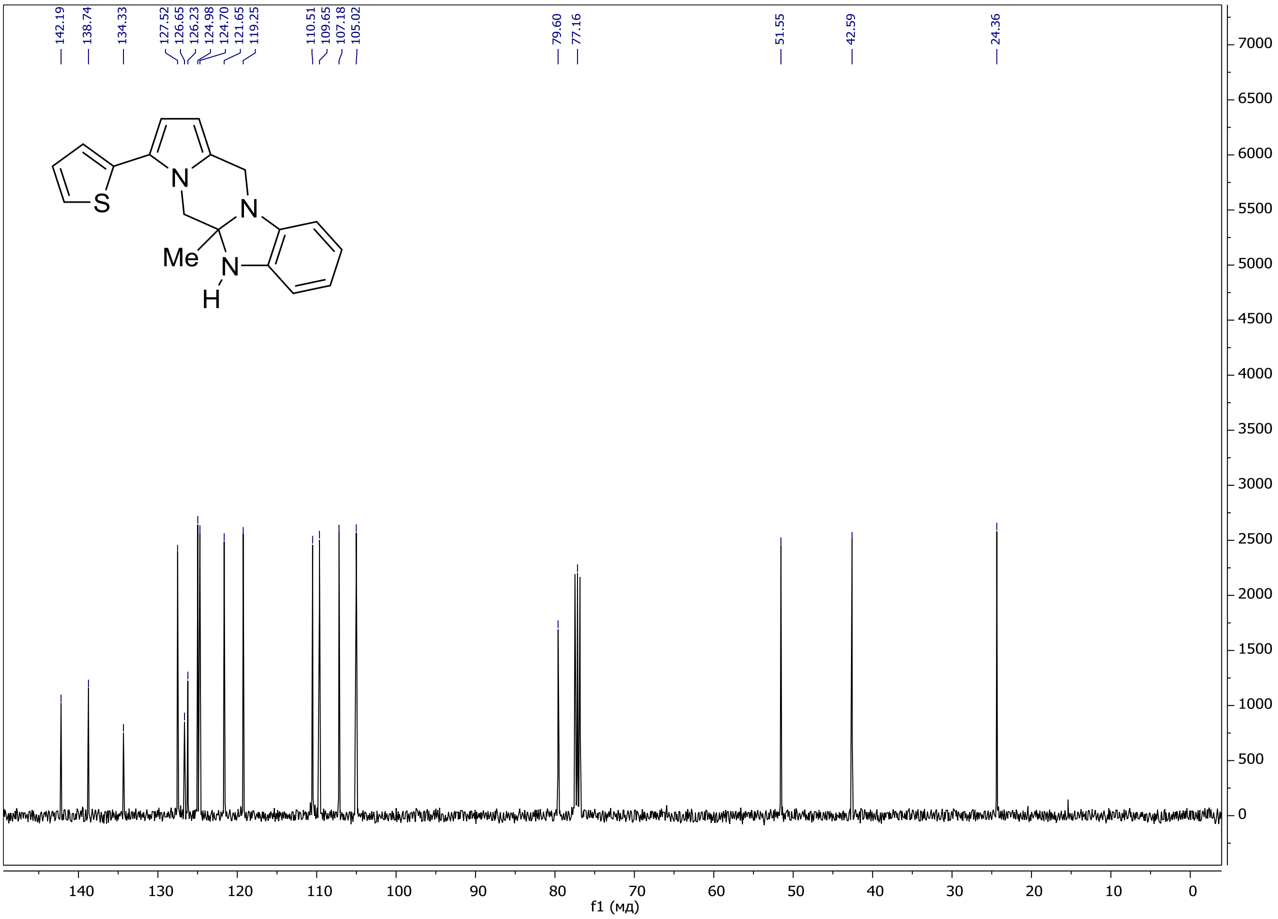

4. NMR spectra of the obtained compounds 5

Figure S42. ^{1}H-NMR (400 MHz, DMSO-d6) spectrum of **5a**

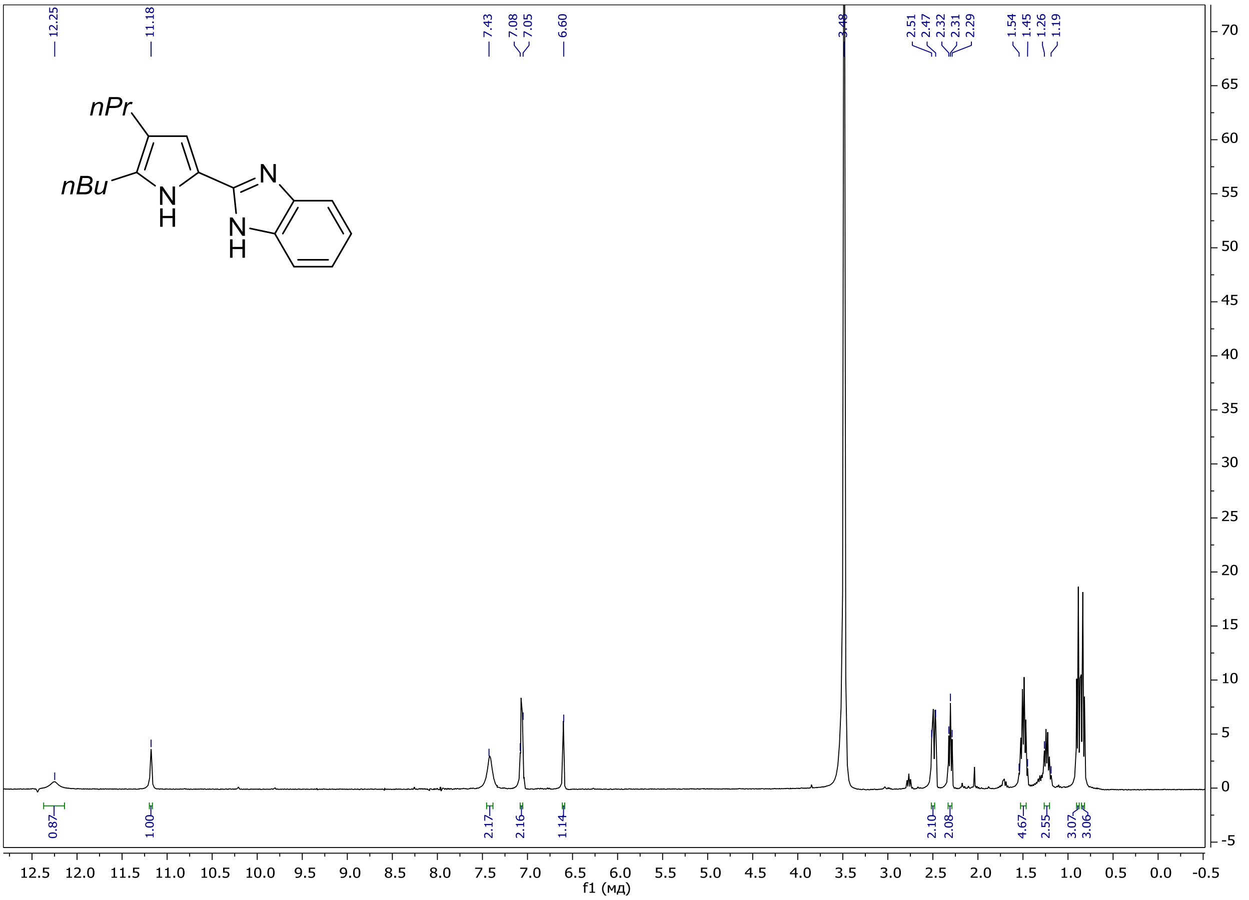

Figure S43. ^{13}C-NMR (400 MHz, DMSO-d6) spectrum of **5a**

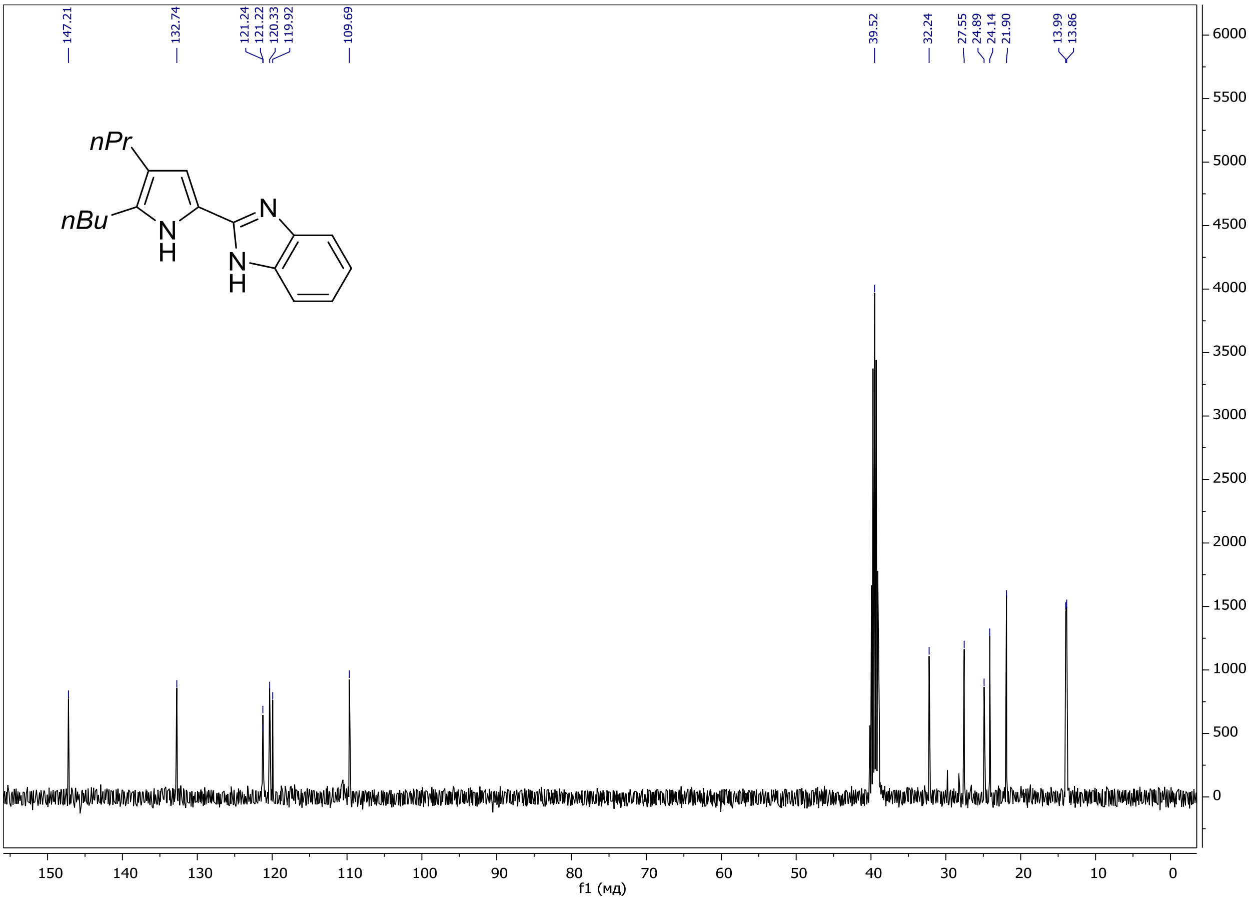

Figure S44. ^{1}H-NMR (400 MHz, DMSO-d6) spectrum of **5l**

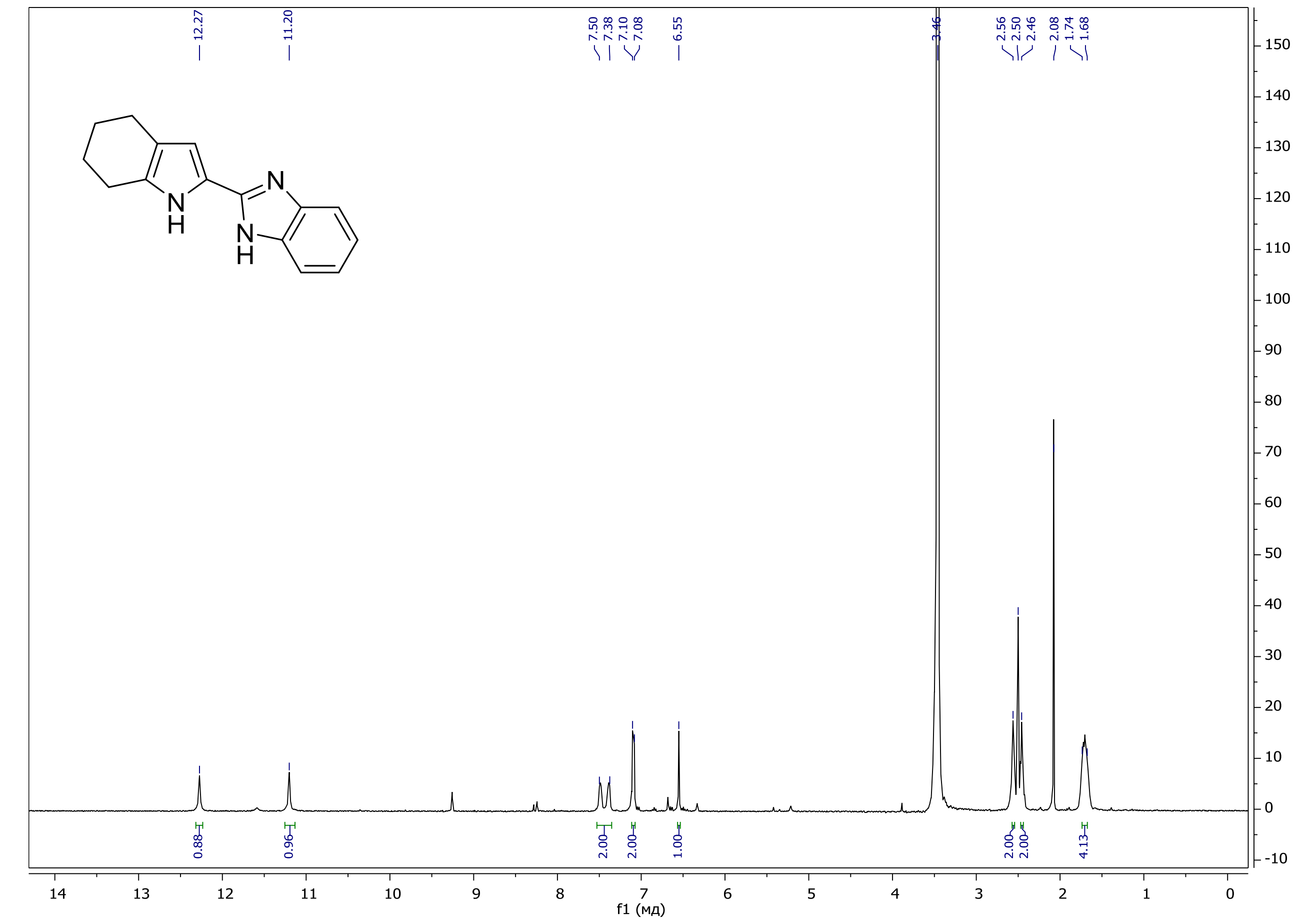

Figure S45. ^{13}C-NMR (100 MHz, DMSO-d6) spectrum of **5l**

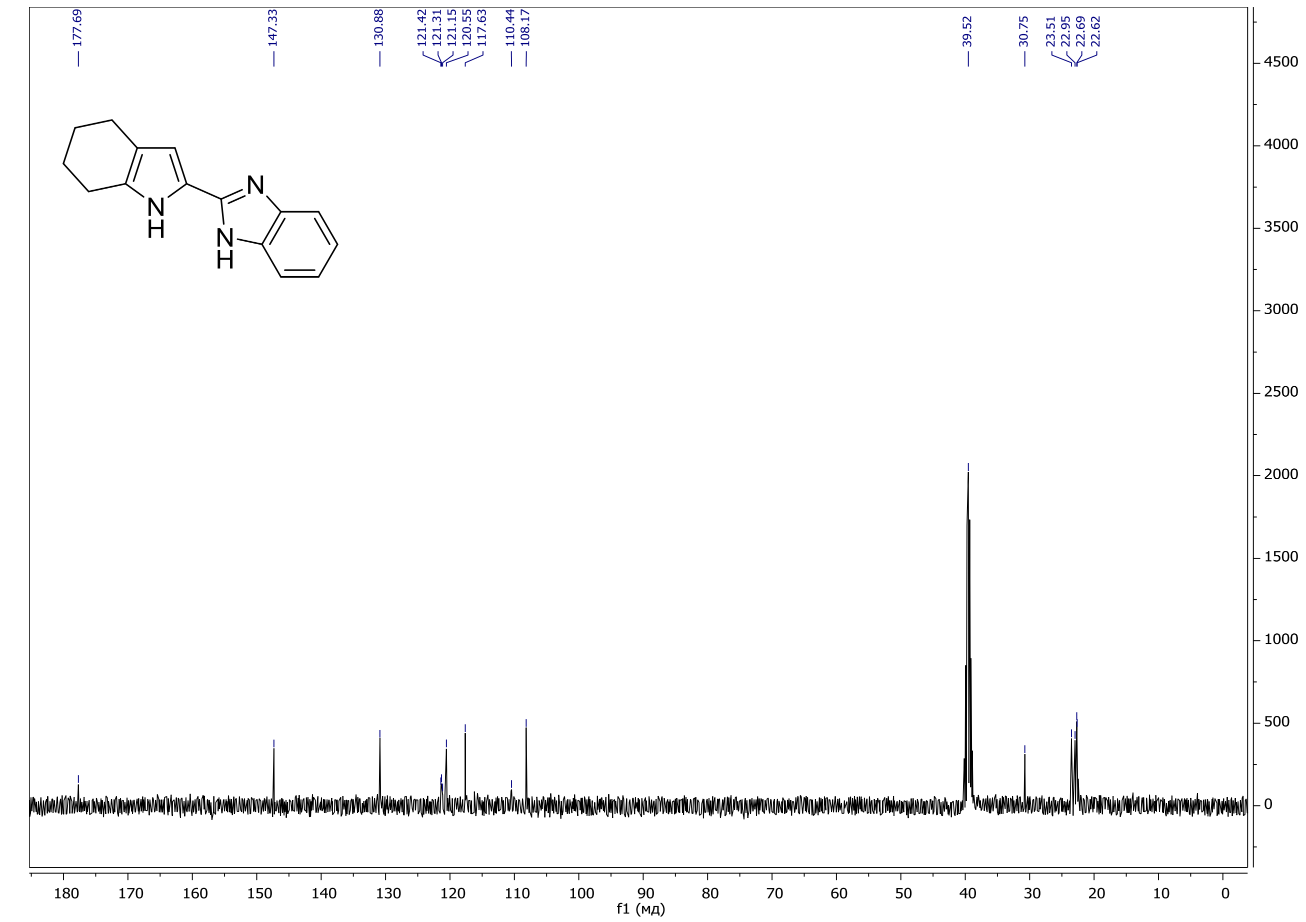

Figure S46. ^{1}H-NMR (400 MHz, DMSO-d6) spectrum of **5c**

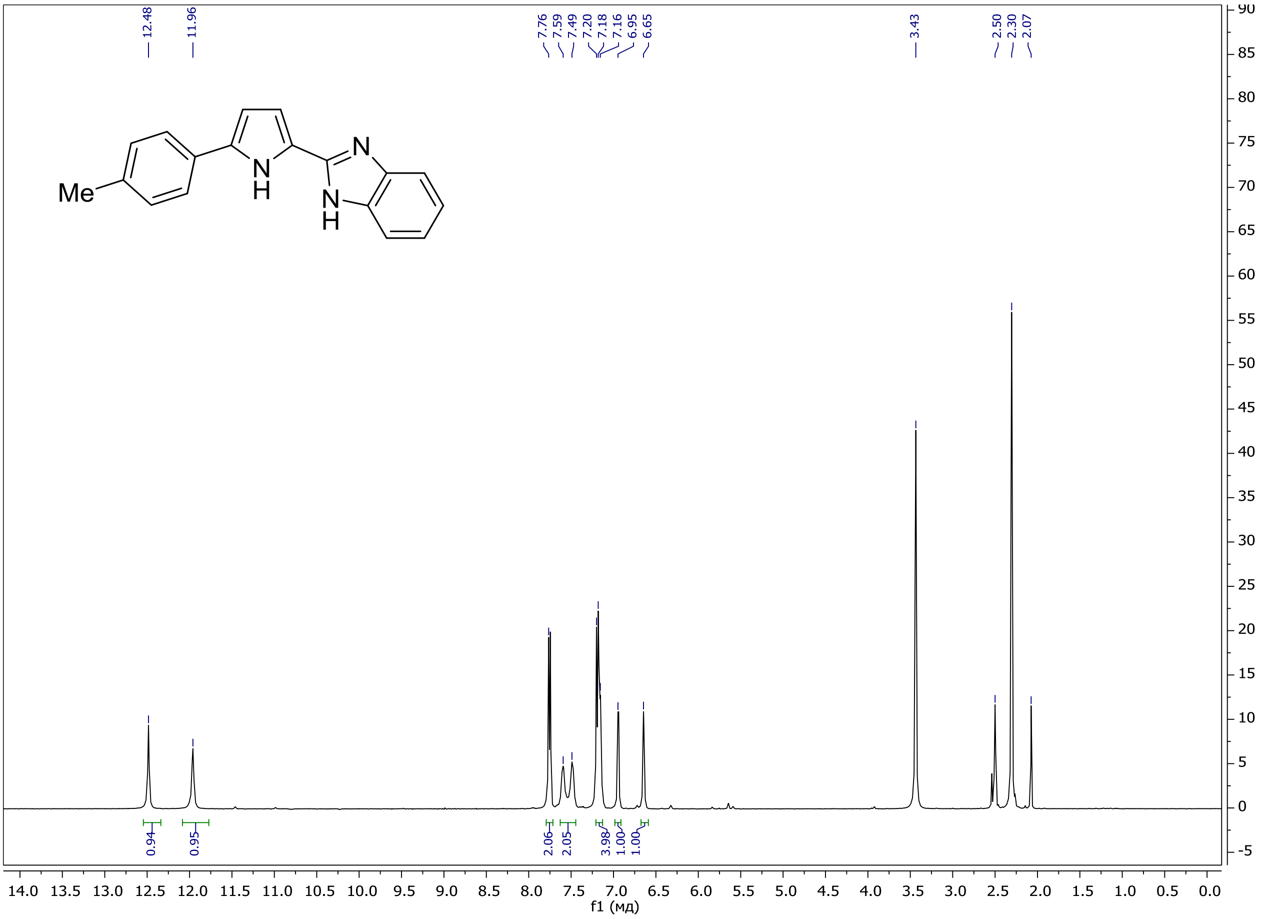

Figure S47. ^{13}C-NMR (100 MHz, DMSO-d6) spectrum of **5c**

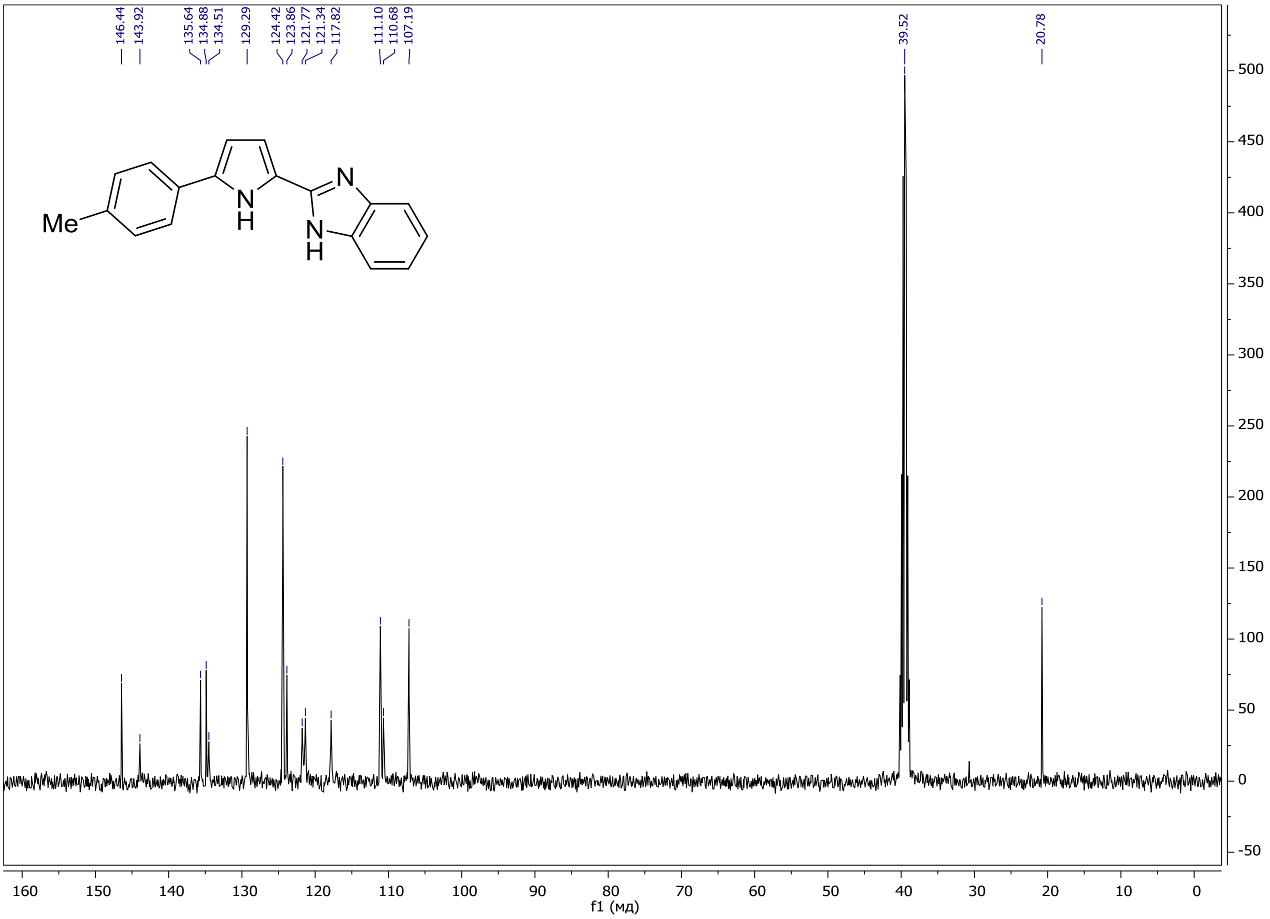

Figure S48. ^{1}H-NMR (400 MHz, DMSO-d6) spectrum of **5d**

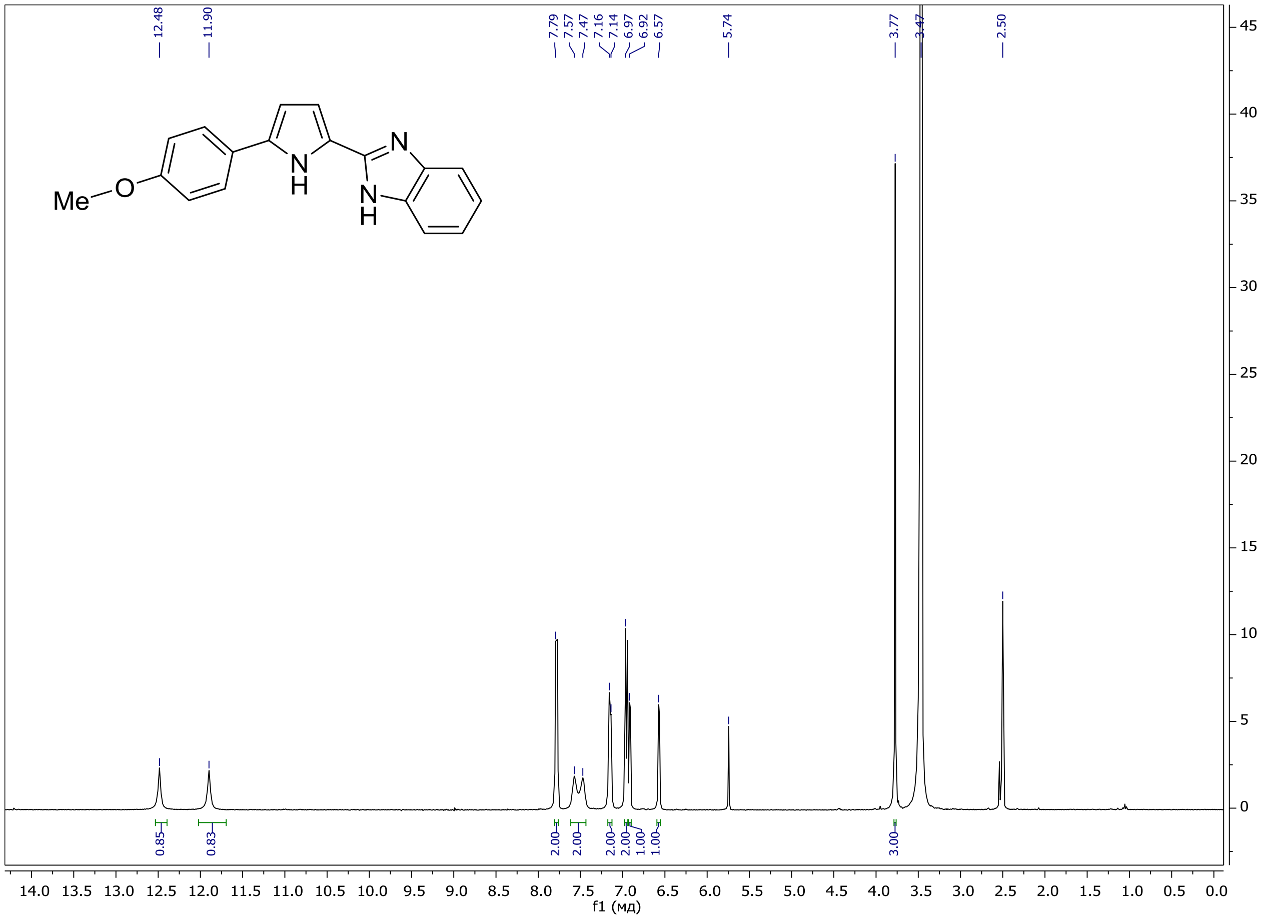

Figure S49. ^{13}C-NMR (100 MHz, DMSO-d6) spectrum of **5d**

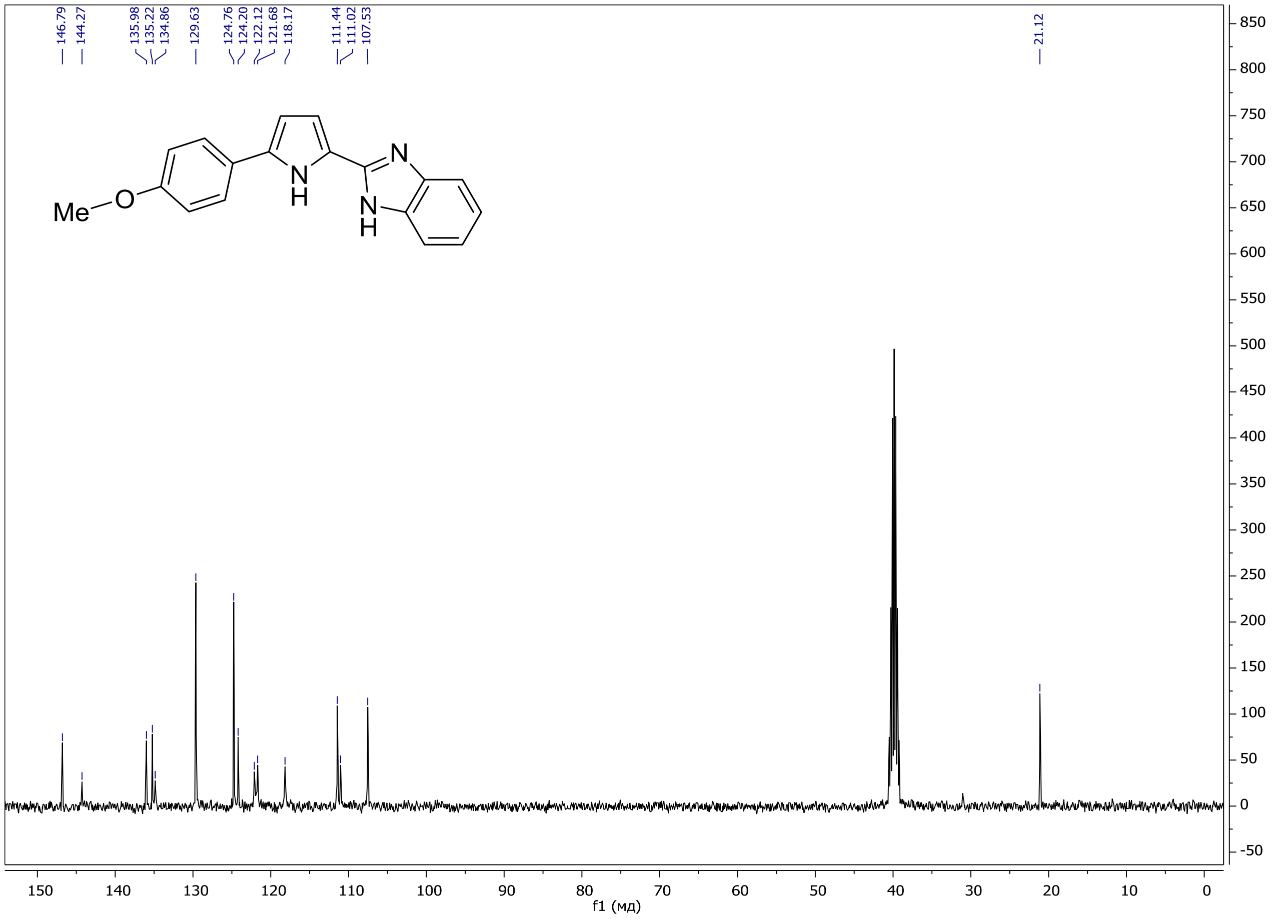

Figure S50. ^{1}H-NMR (400 MHz, DMSO-d6) spectrum of **5e**

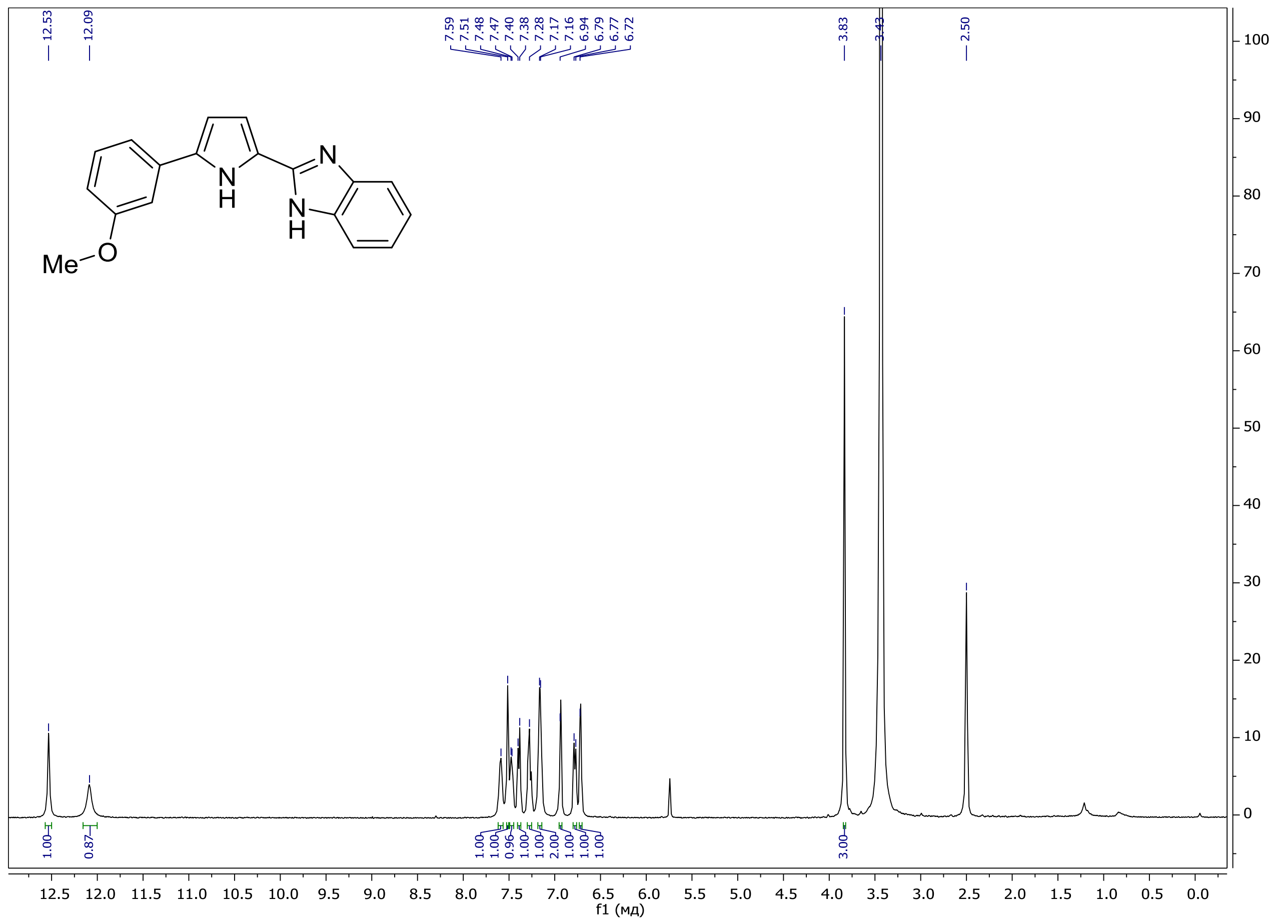

Figure S51. ^{13}C-NMR (100 MHz, DMSO-d6) spectrum of **5e**

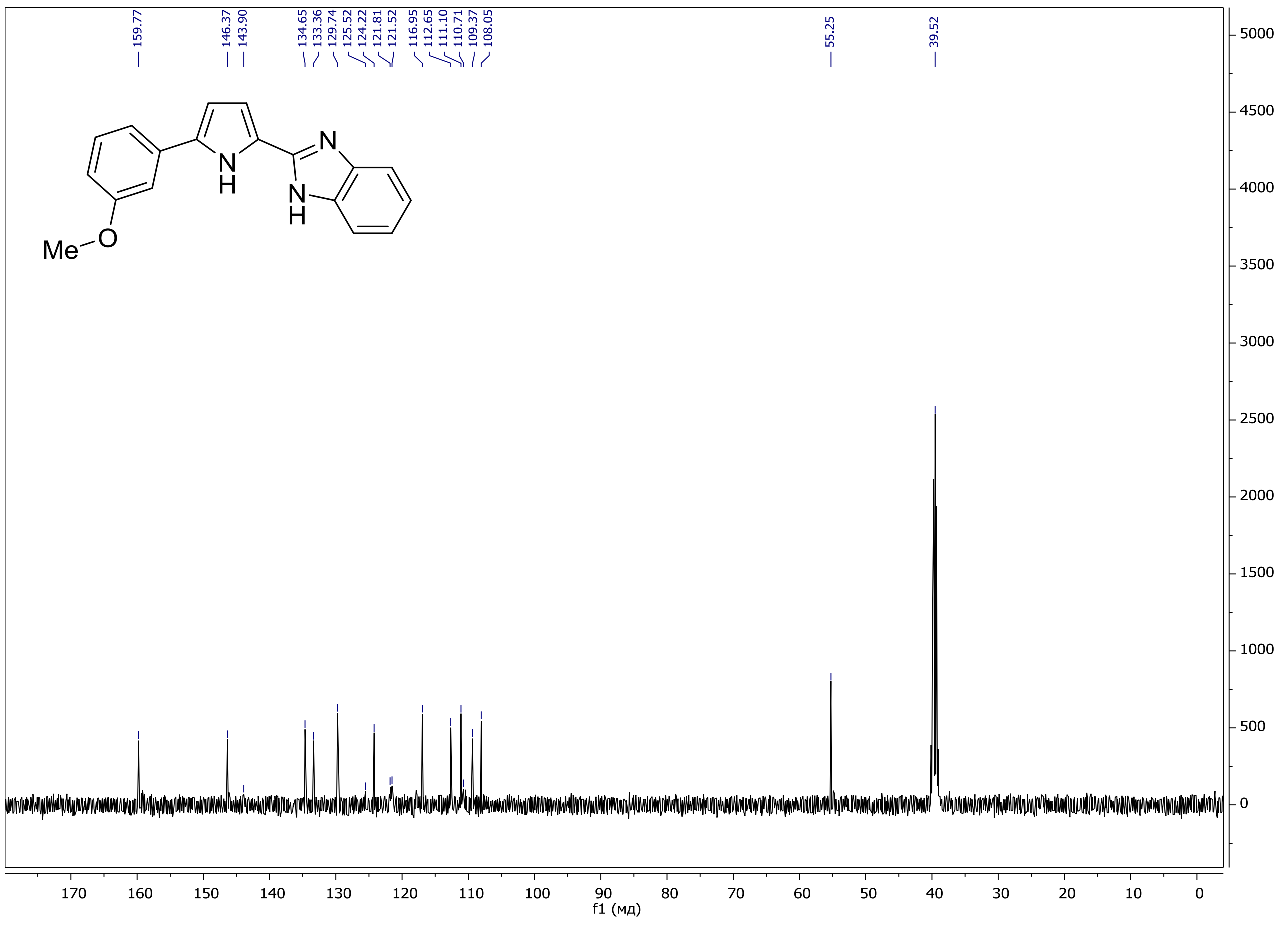

Figure S52. ^{1}H-NMR (400 MHz, DMSO-d6) spectrum of **5f**

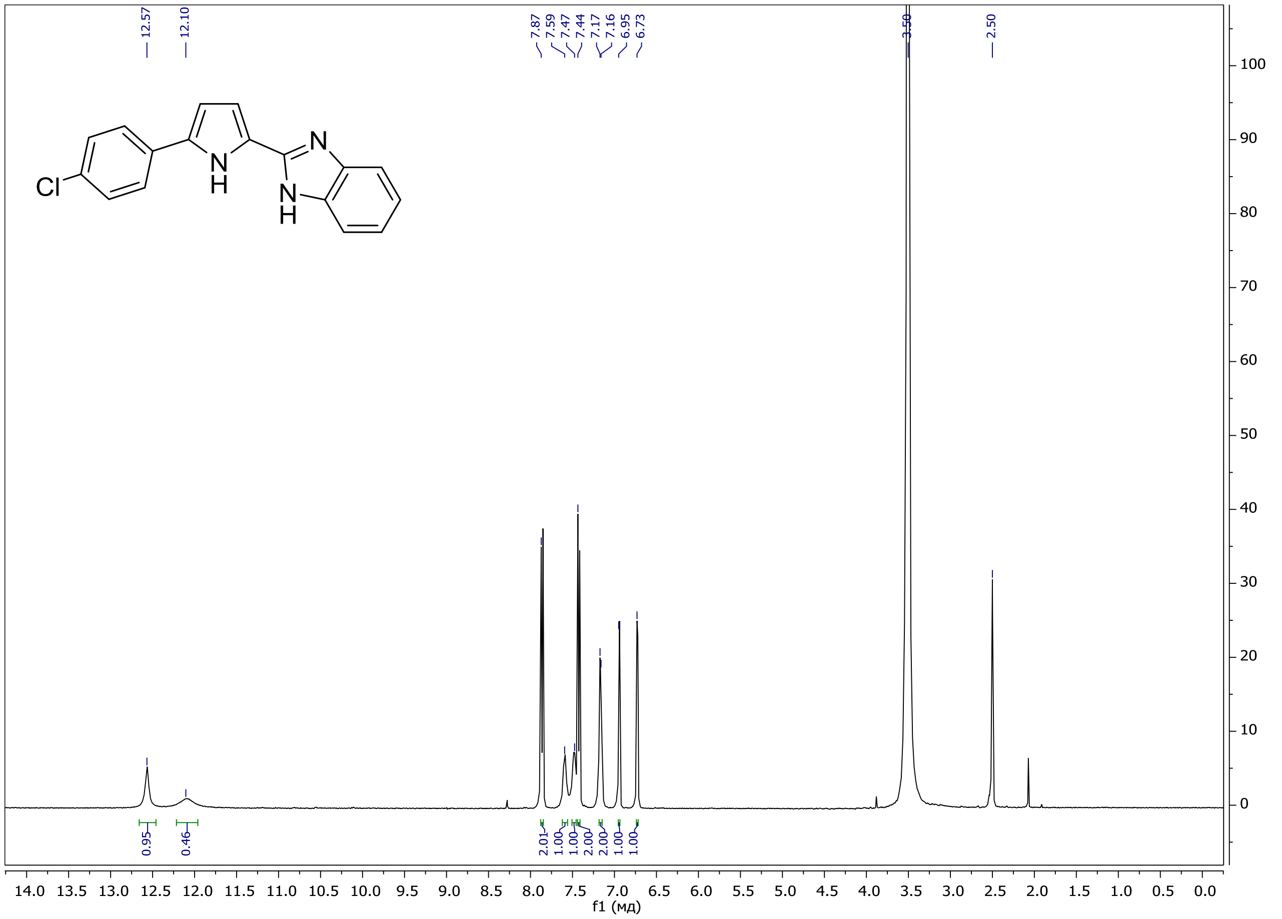

Figure S53. ^{13}C-NMR (100 MHz, DMSO-d6) spectrum of **5f**

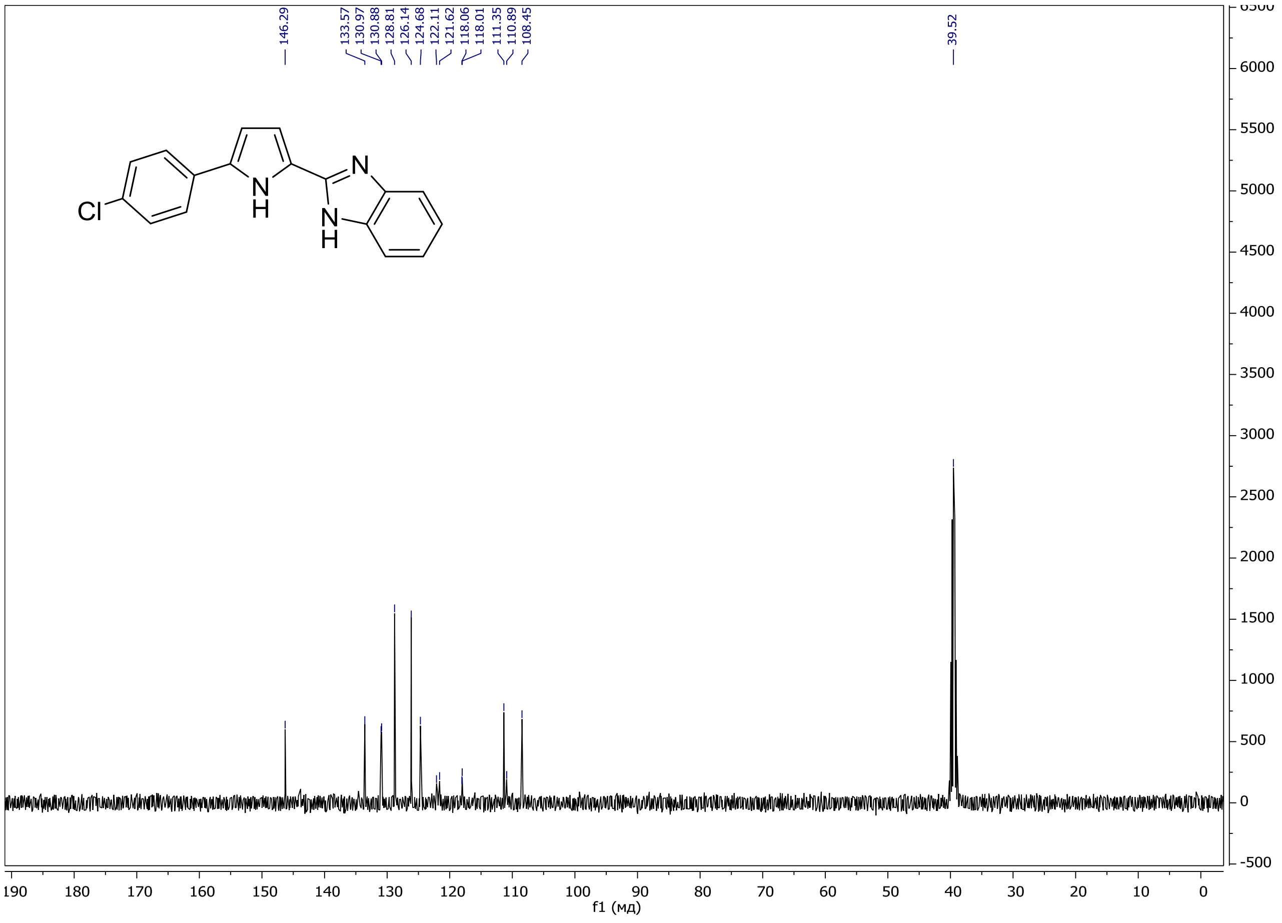

Figure S54. ^{1}H-NMR (400 MHz, DMSO-d6) spectrum of **5g**

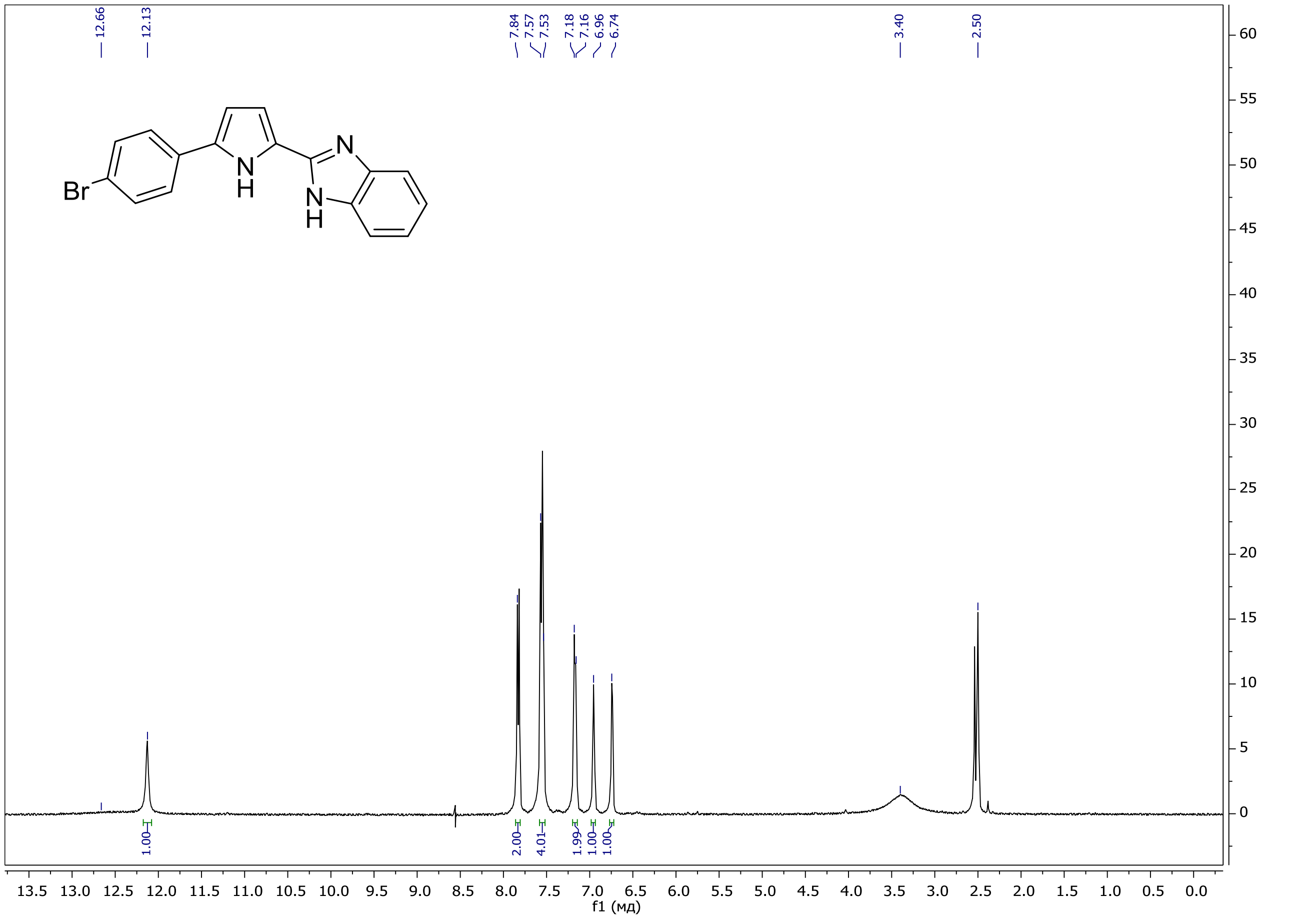

Figure S55. ^{13}C-NMR (100 MHz, DMSO-d6) spectrum of **5g**

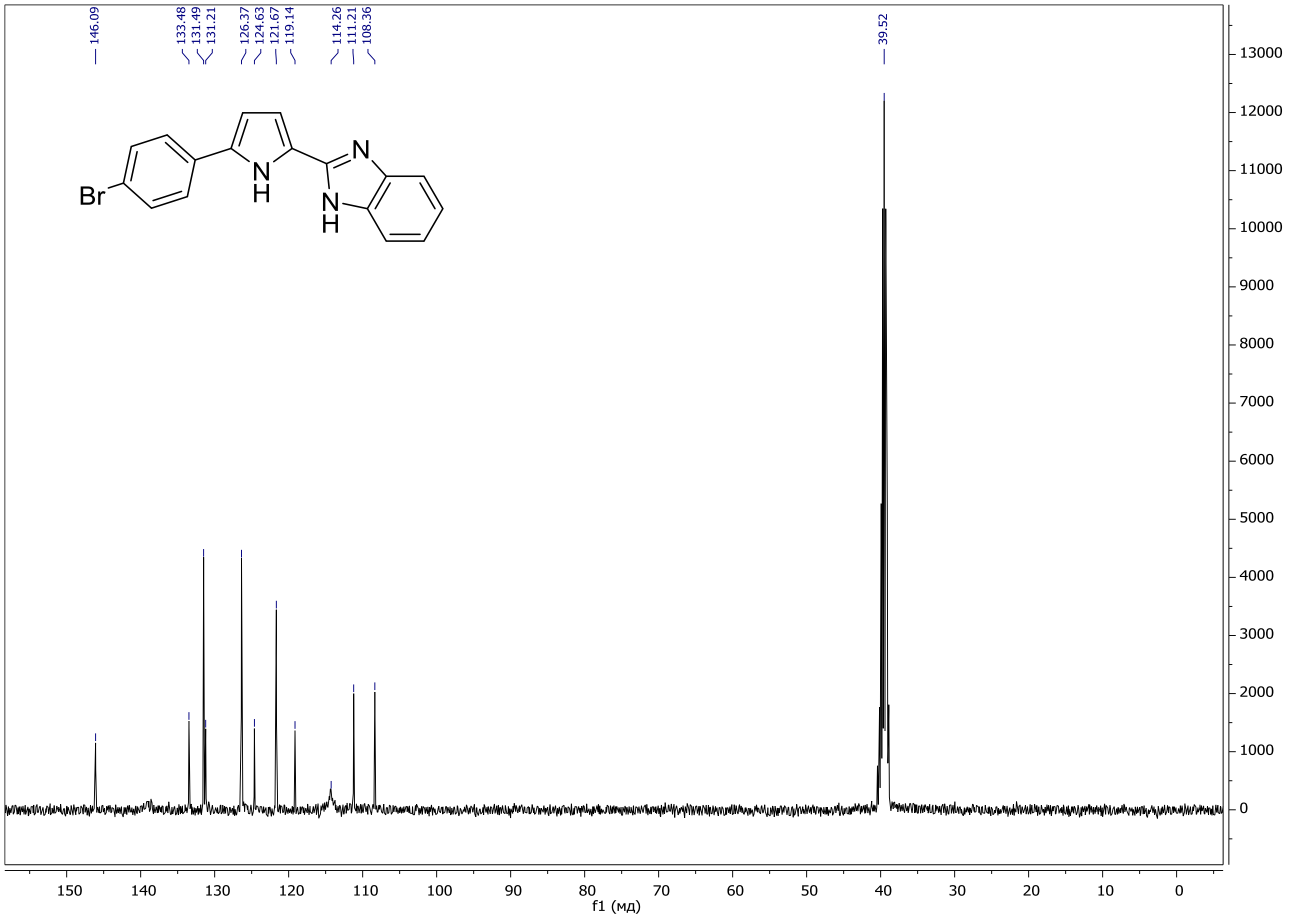

Figure S56. ^{1}H-NMR (400 MHz, DMSO-d6) spectrum of **5h**

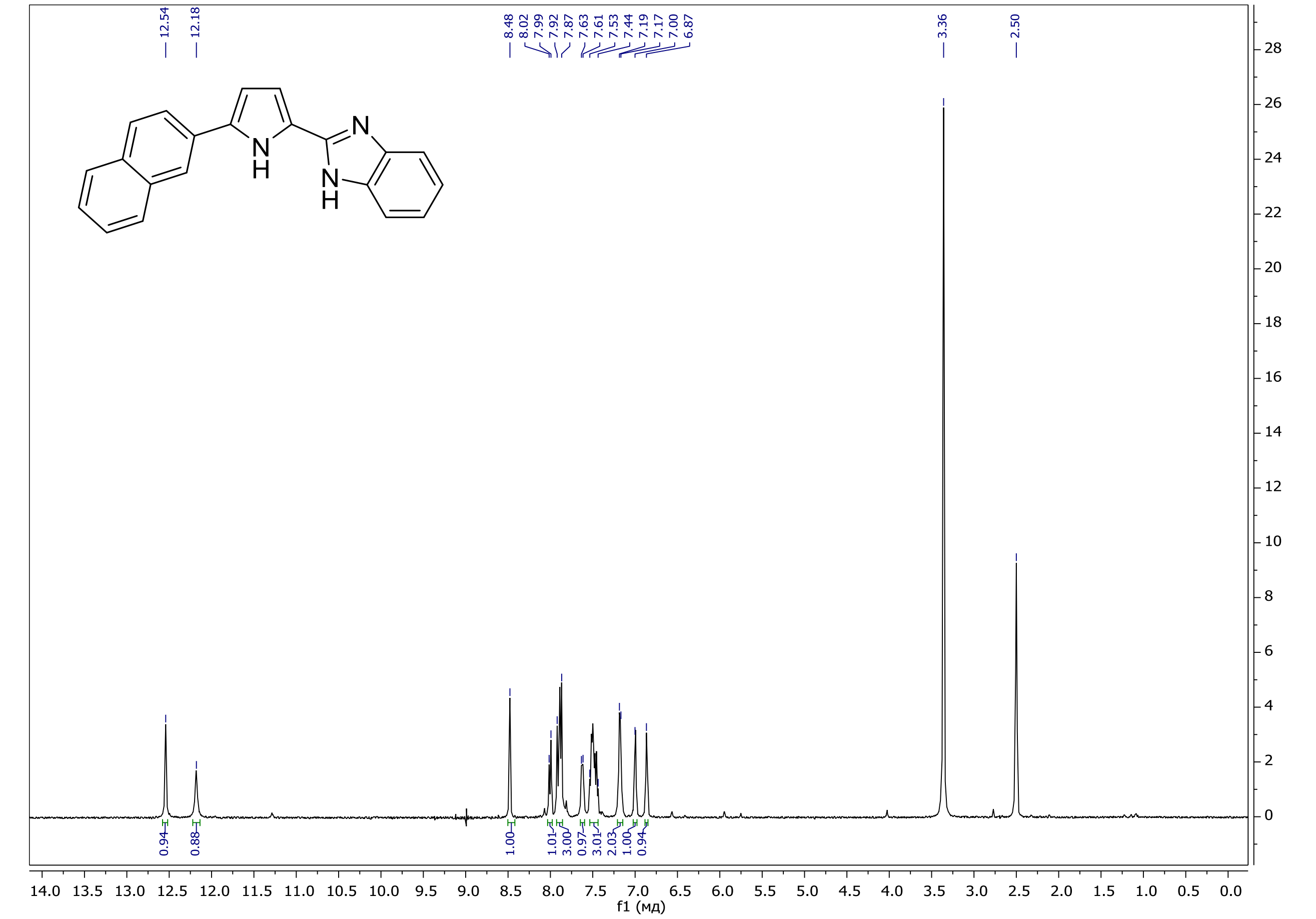

Figure S57. ^{13}C-NMR (100 MHz, DMSO-d6) spectrum of **5h**

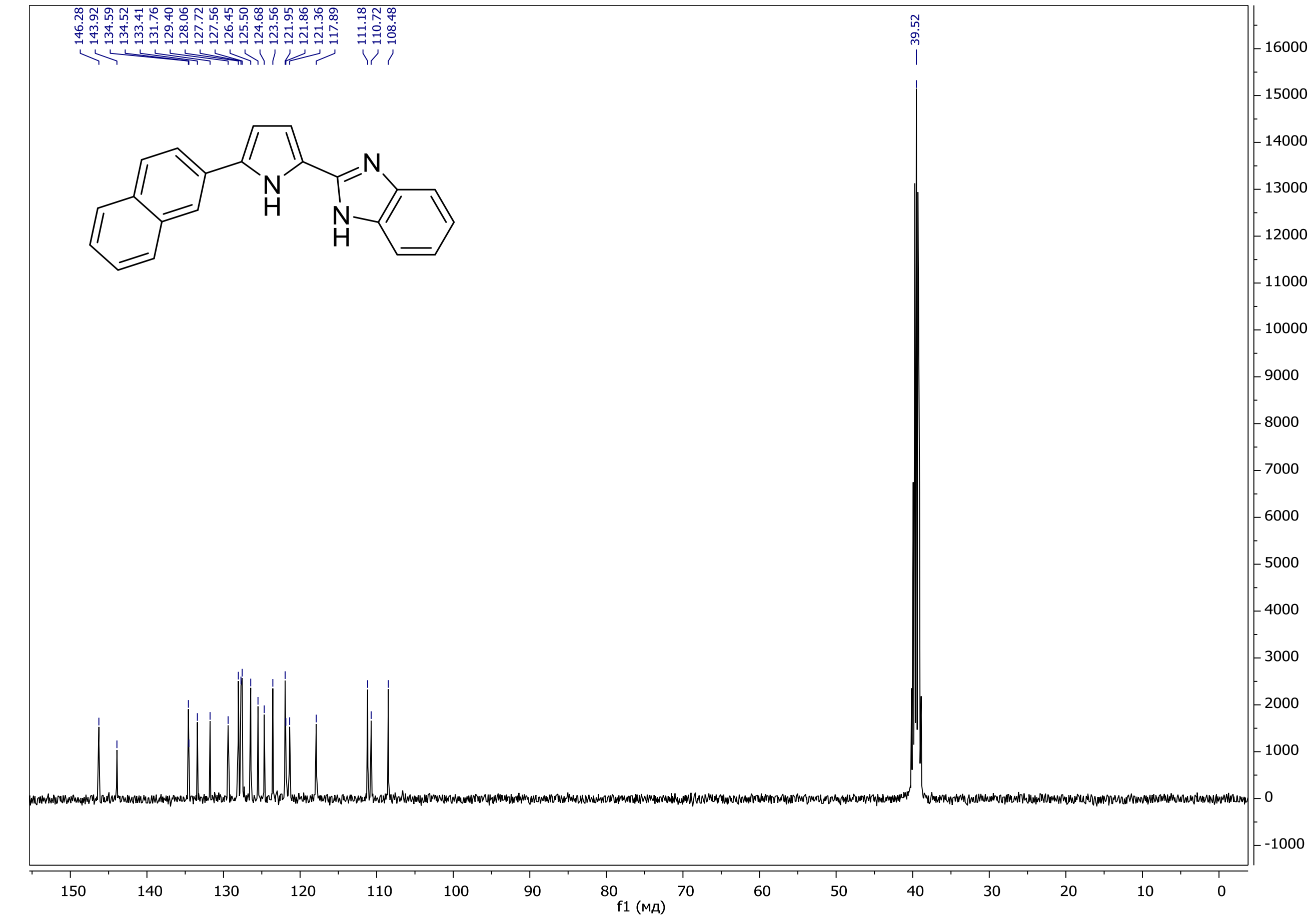

Figure S58. ¹H-NMR (400 MHz, DMSO-d6) spectrum of **5i**

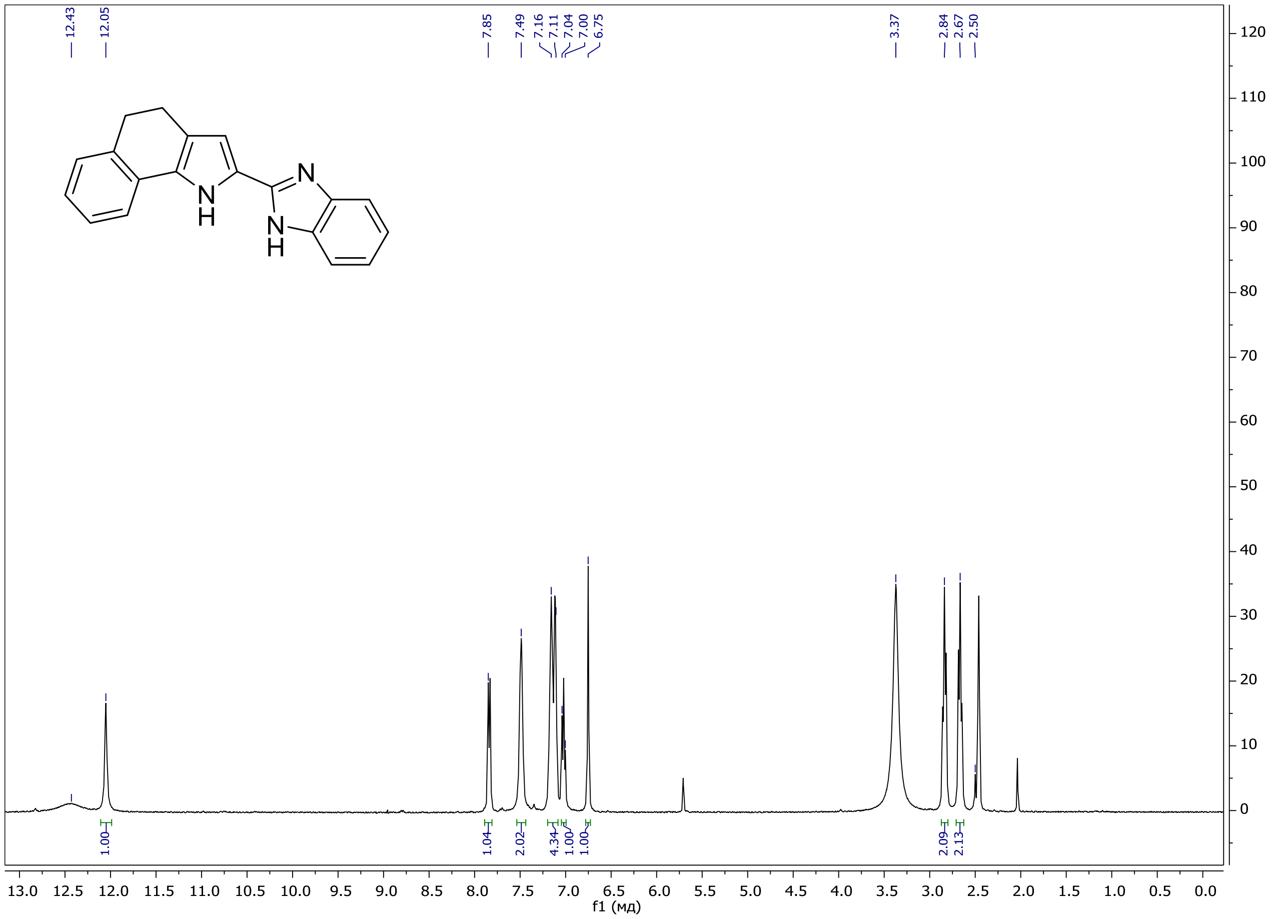

Figure S59. ¹³C-NMR (100 MHz, DMSO-d6) spectrum of **5i**

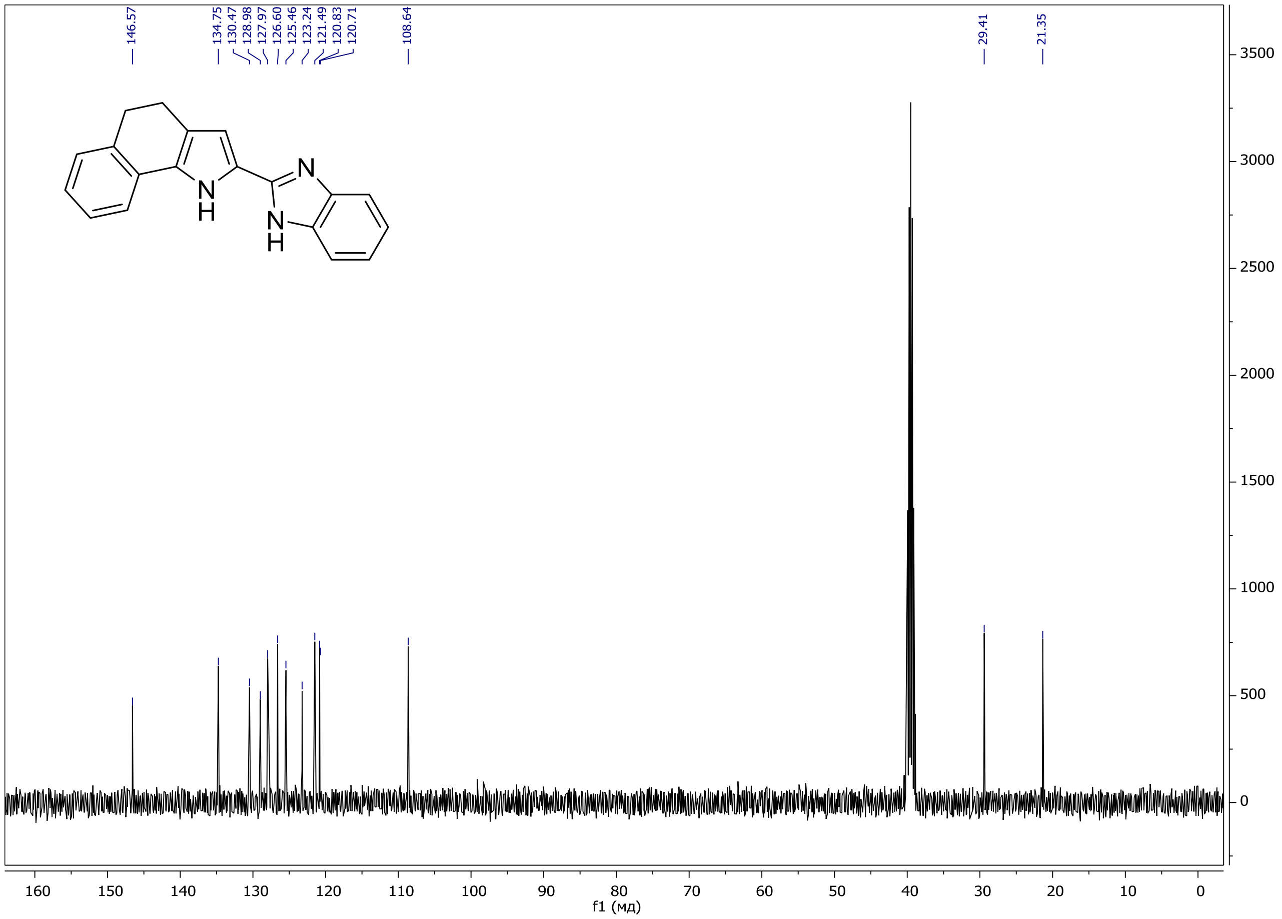

5. NMR spectra of the obtained compounds 6

Figure S60. ^{1}H-NMR (400 MHz, $CDCl_3$) spectrum of **6k**

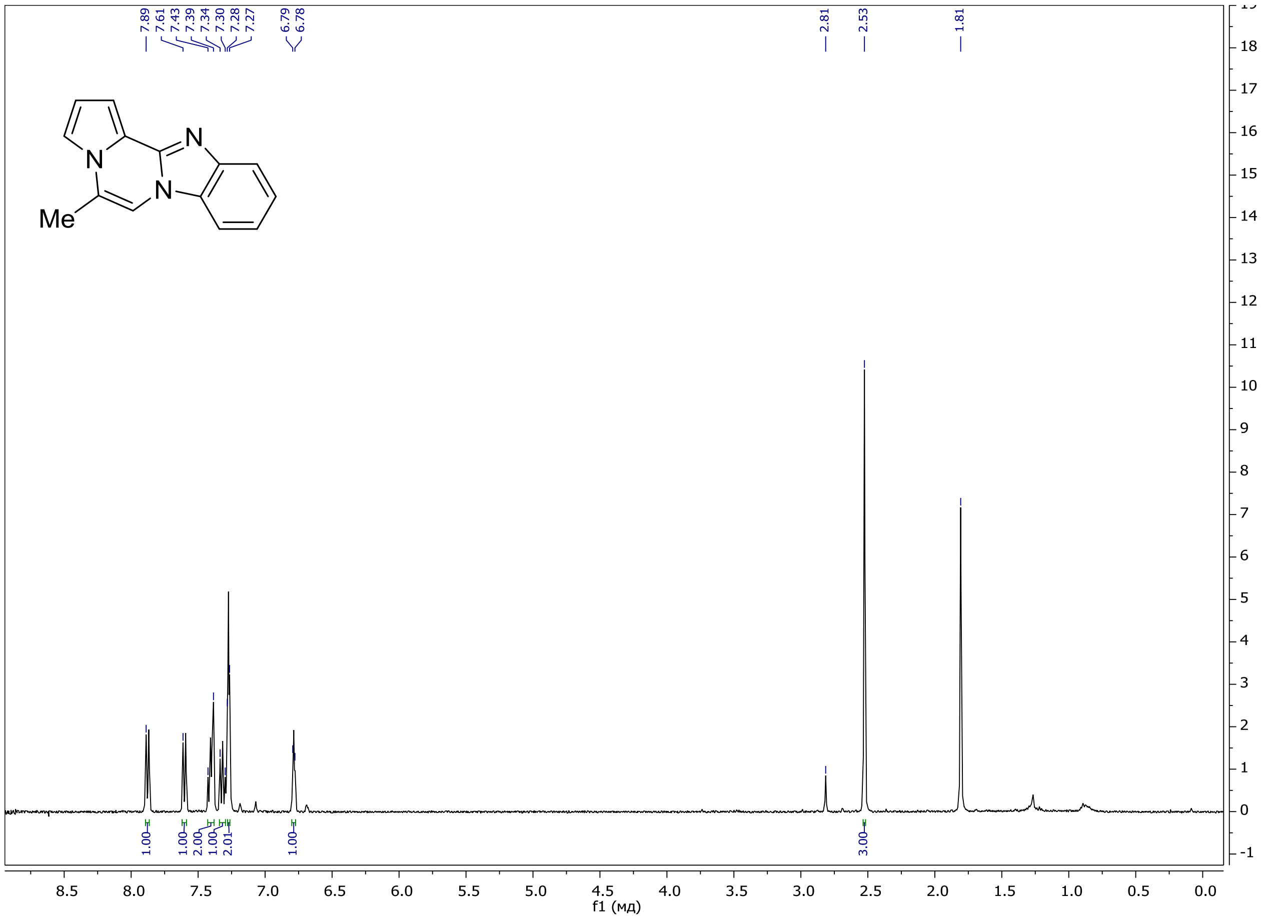

Figure S61. ^{13}C-NMR (100 MHz, $CDCl_3$) spectrum of **6k**

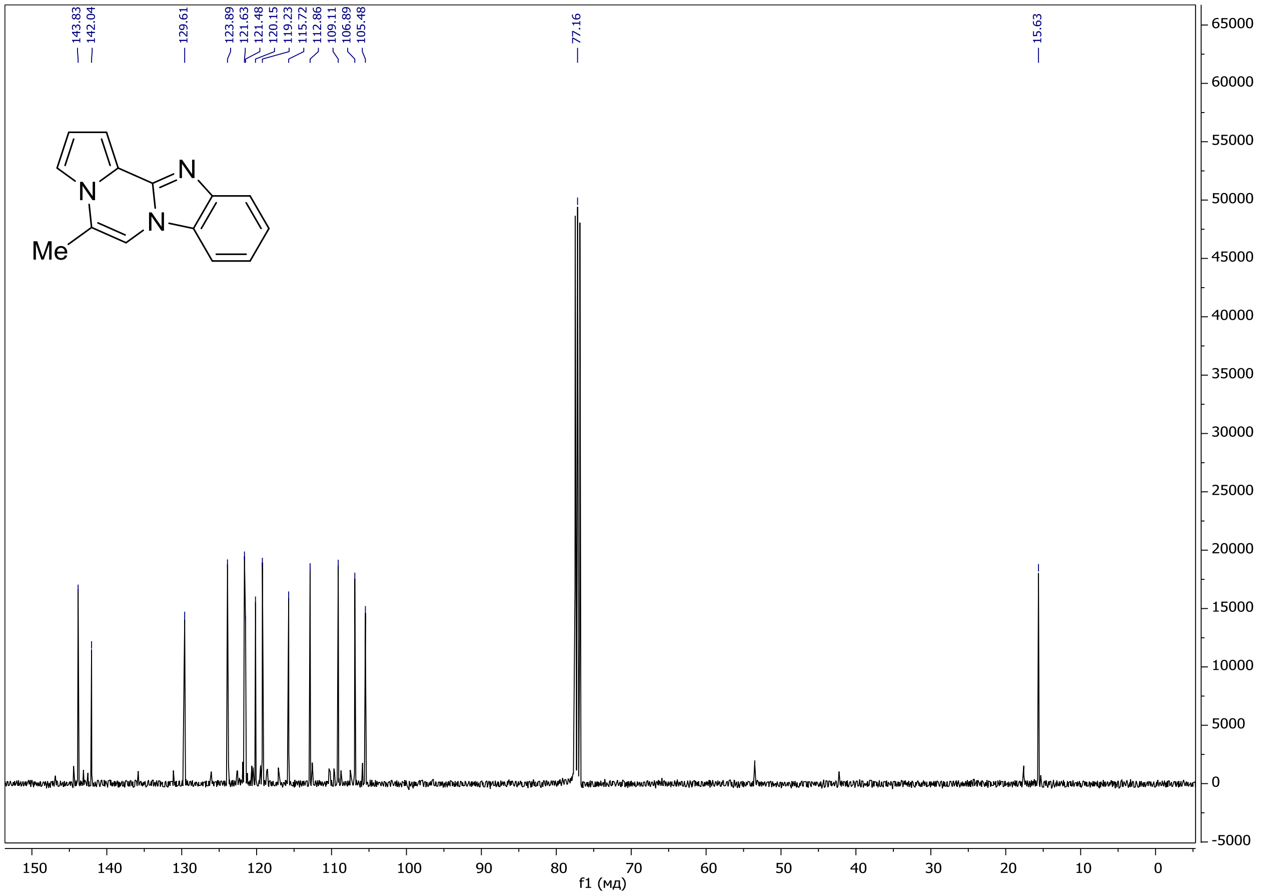

Figure S62. ^{1}H-NMR (400 MHz, $CDCl_3$) spectrum of **6a**

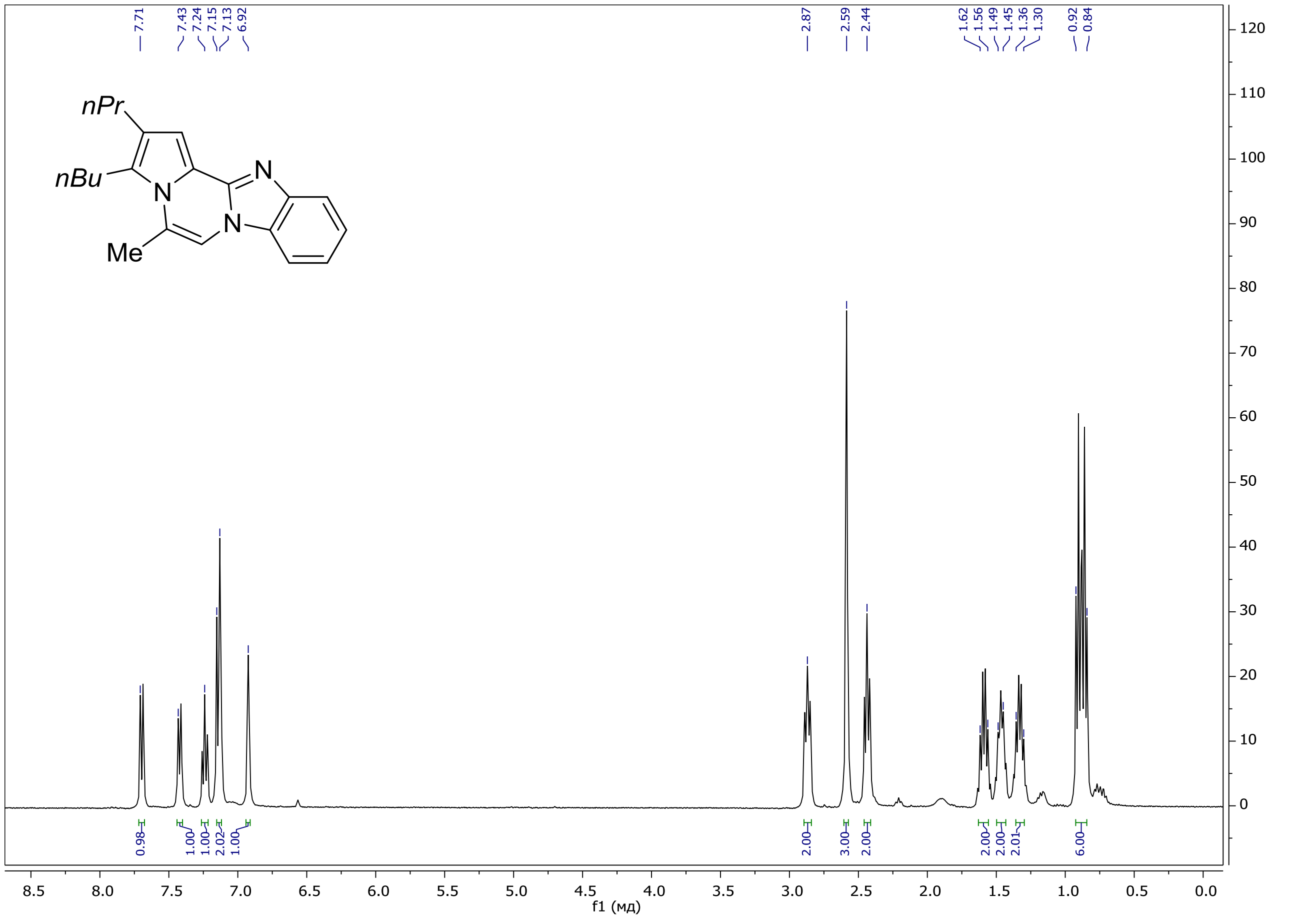

Figure S63. ^{13}C-NMR (100 MHz, $CDCl_3$) spectrum of **6a**

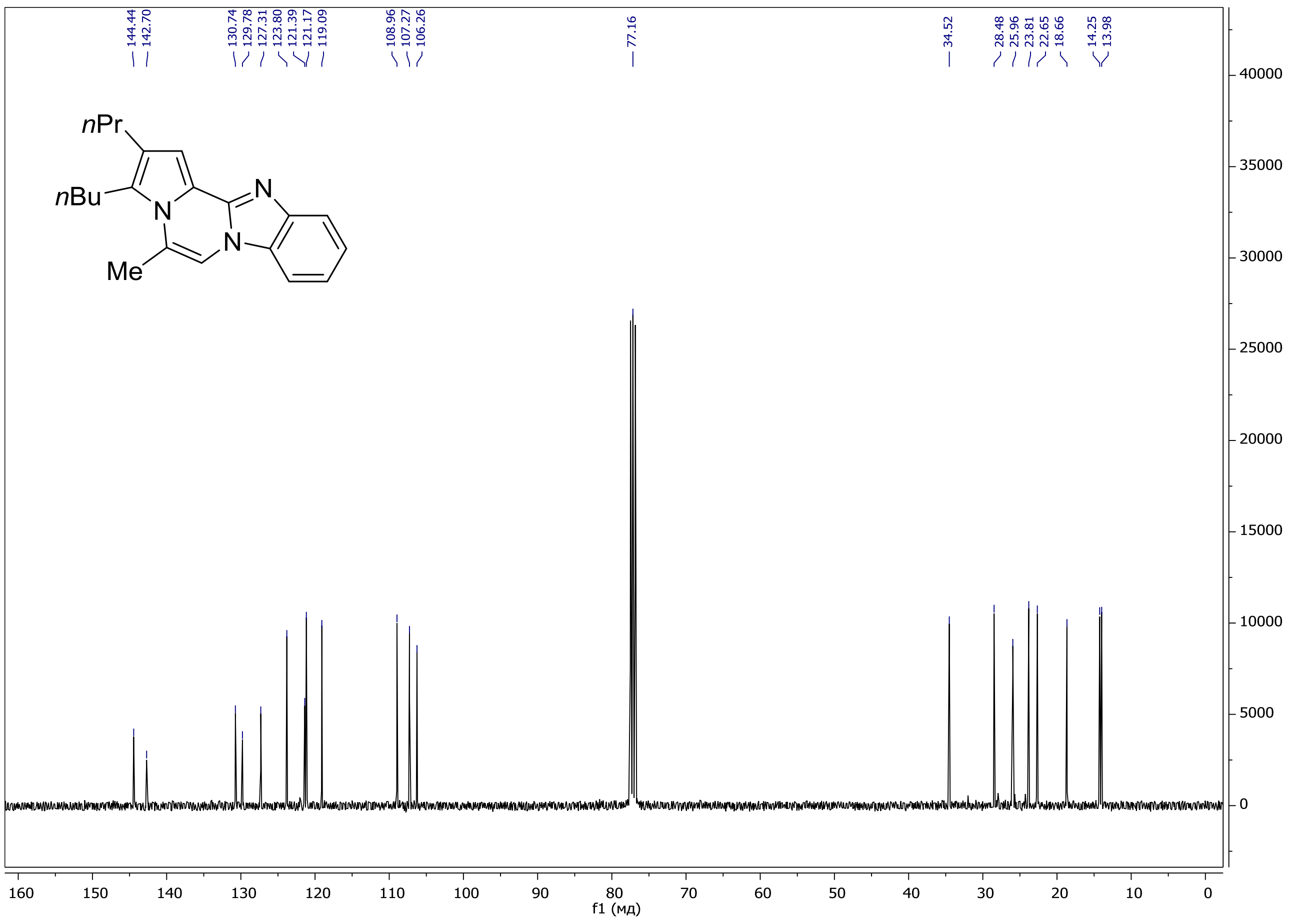

Figure S64. ^{1}H-NMR (400 MHz, $CDCl_3$) spectrum of **6l**

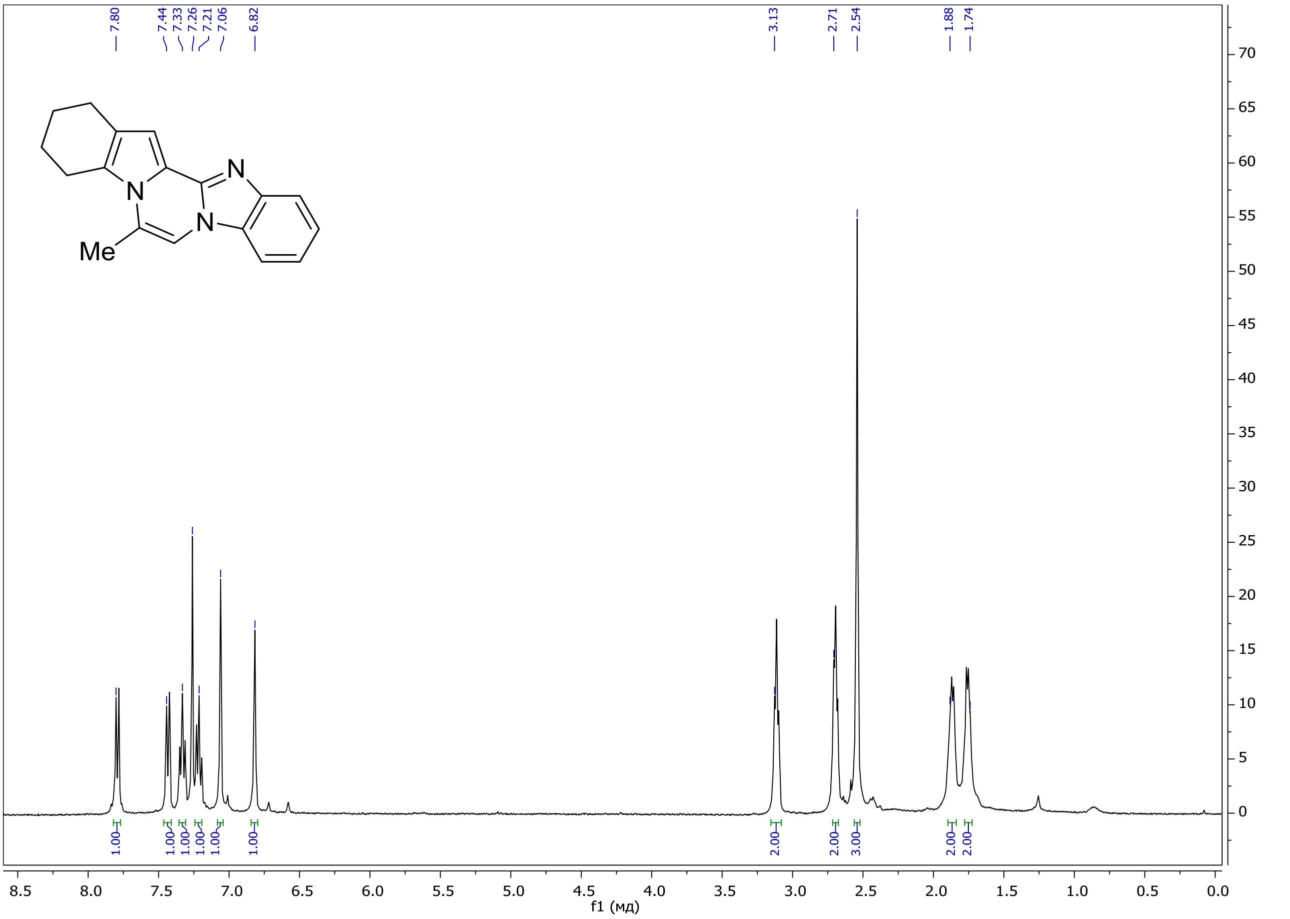

Figure S65. ^{13}C-NMR (100 MHz, $CDCl_3$) spectrum of **6l**

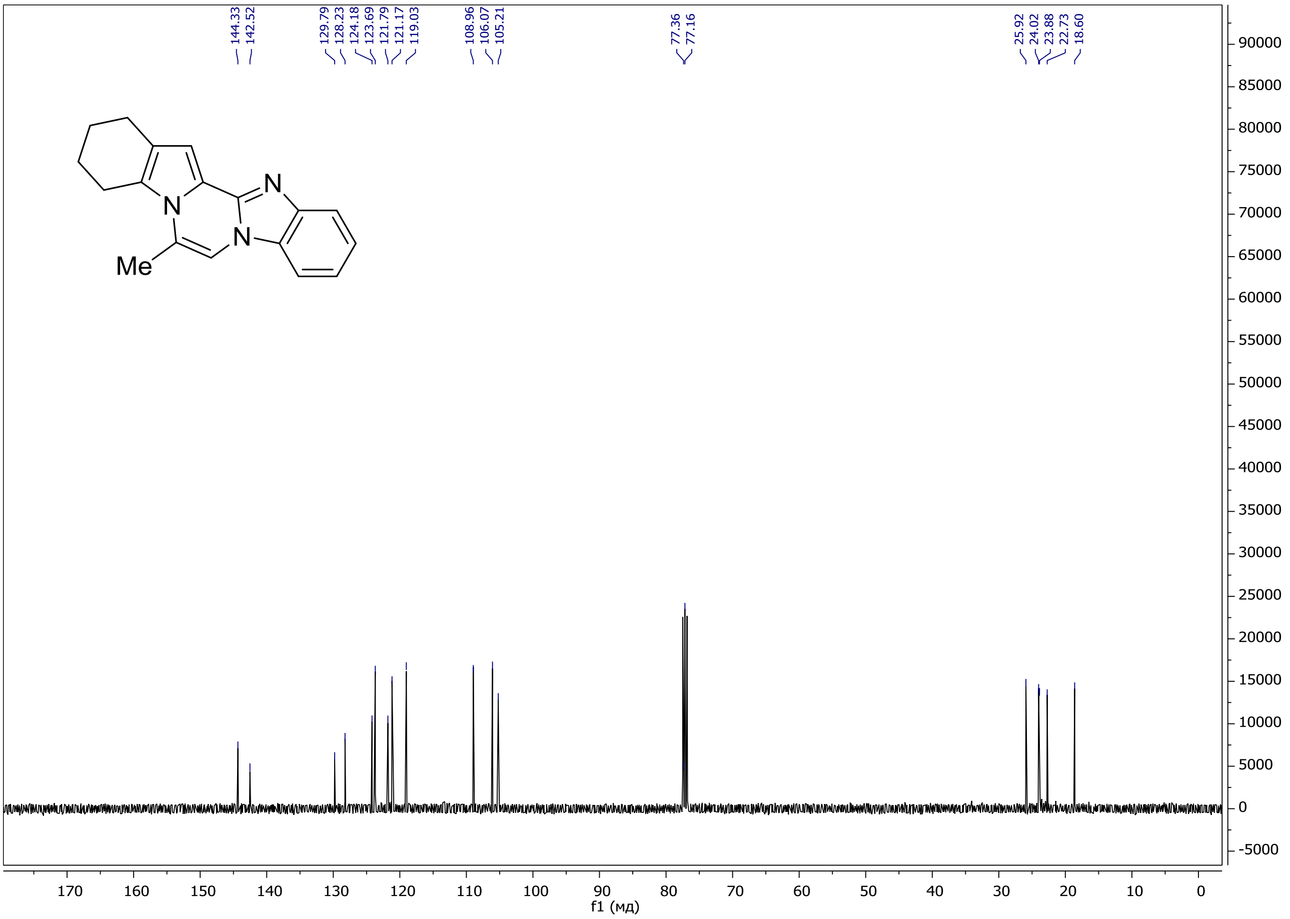

Figure S66. ^{1}H-NMR (400 MHz, $CDCl_3$) spectrum of **6b**

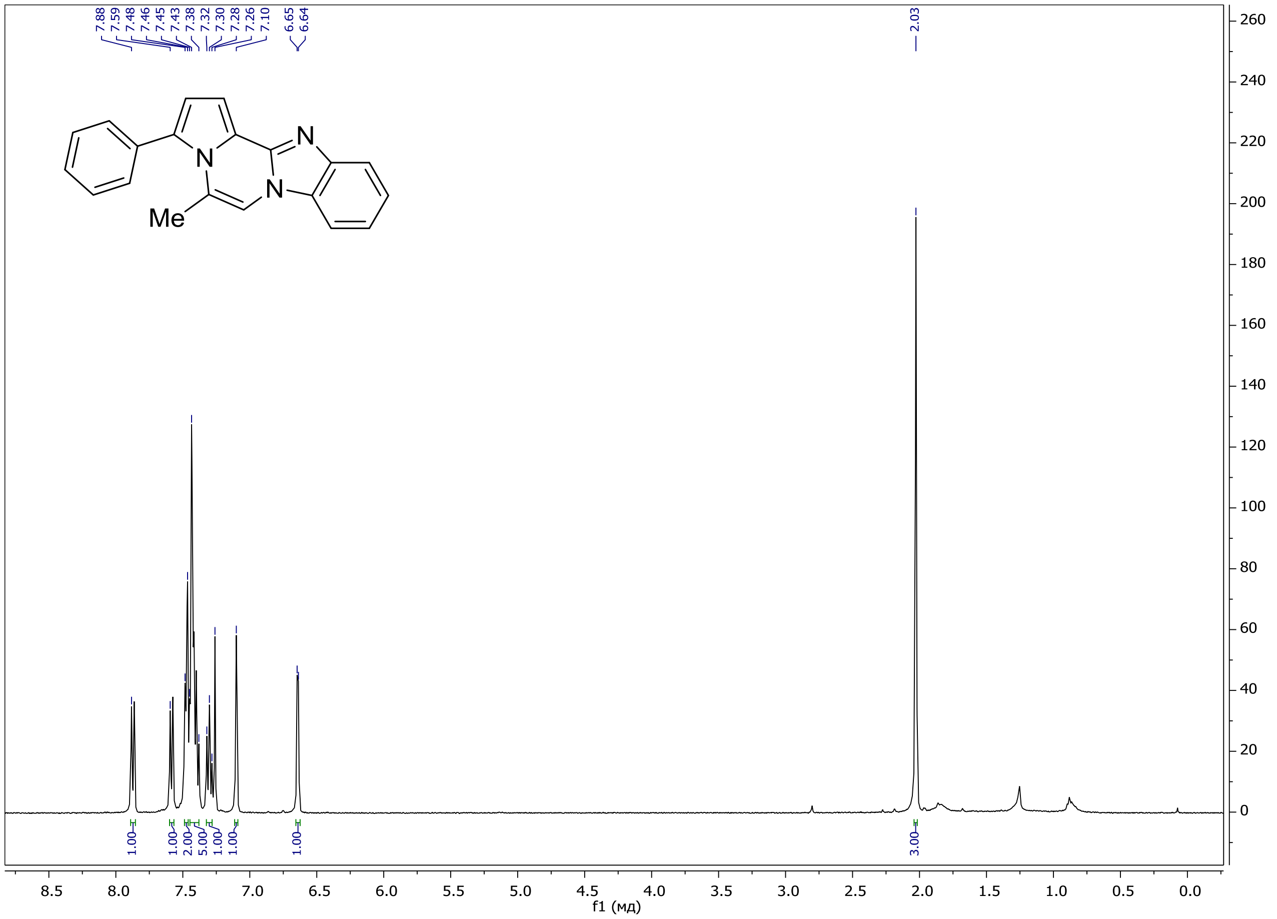

Figure S67. ^{13}C-NMR (100 MHz, $CDCl_3$) spectrum of **6b**

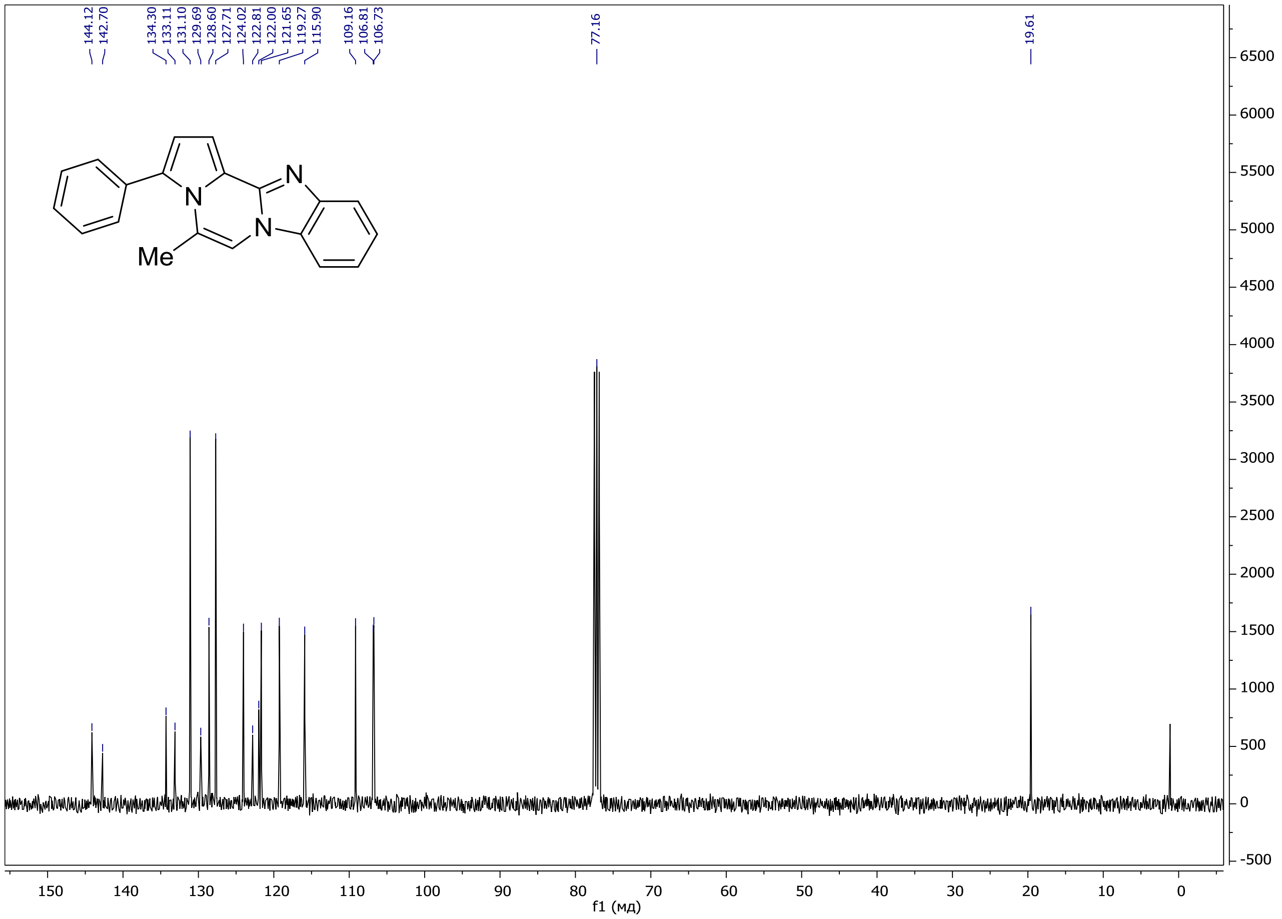

Figure S68. ^{1}H-NMR (400 MHz, $CDCl_3$) spectrum of **6c**

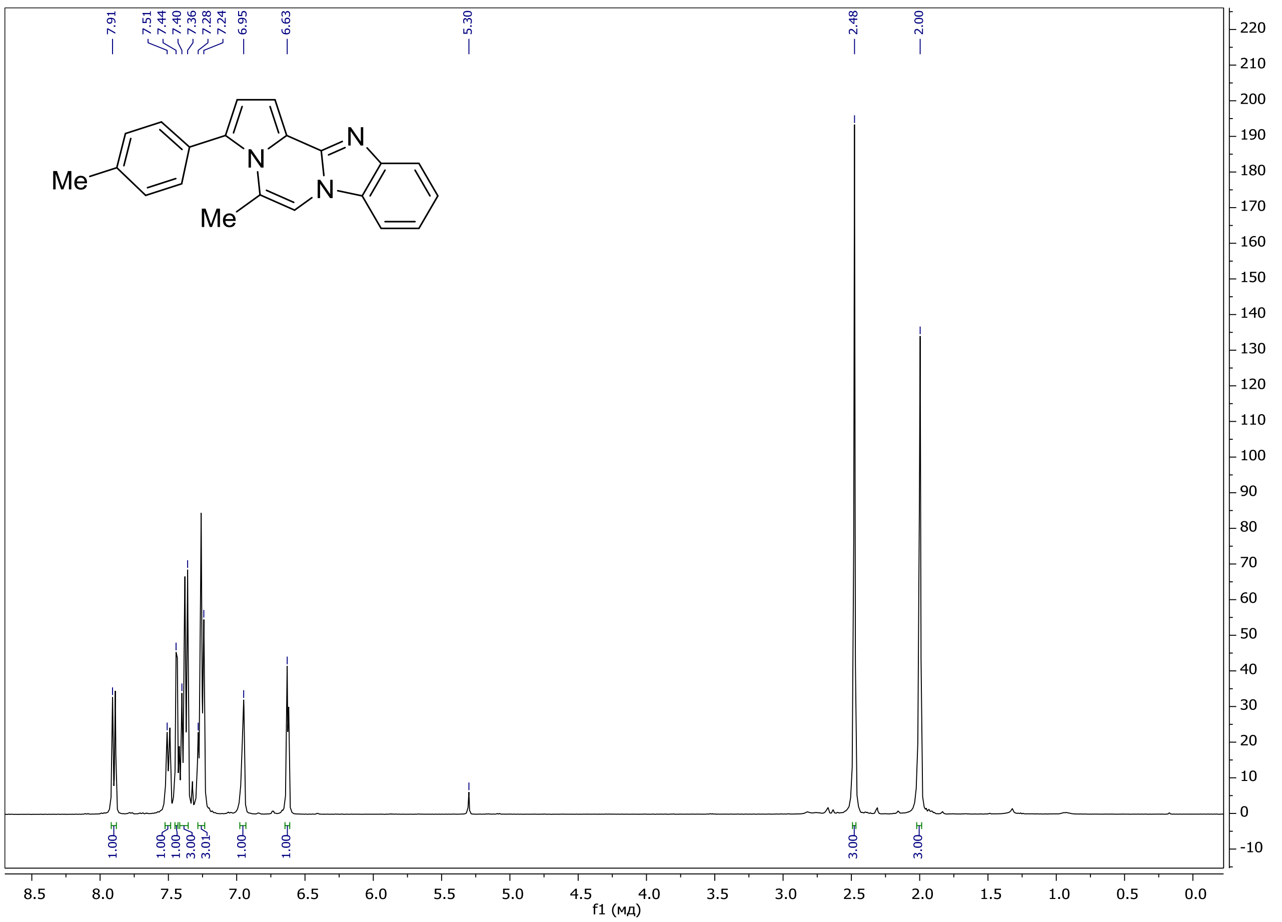

Figure S69. ^{13}C-NMR (100 MHz, $CDCl_3$) spectrum of **6c**

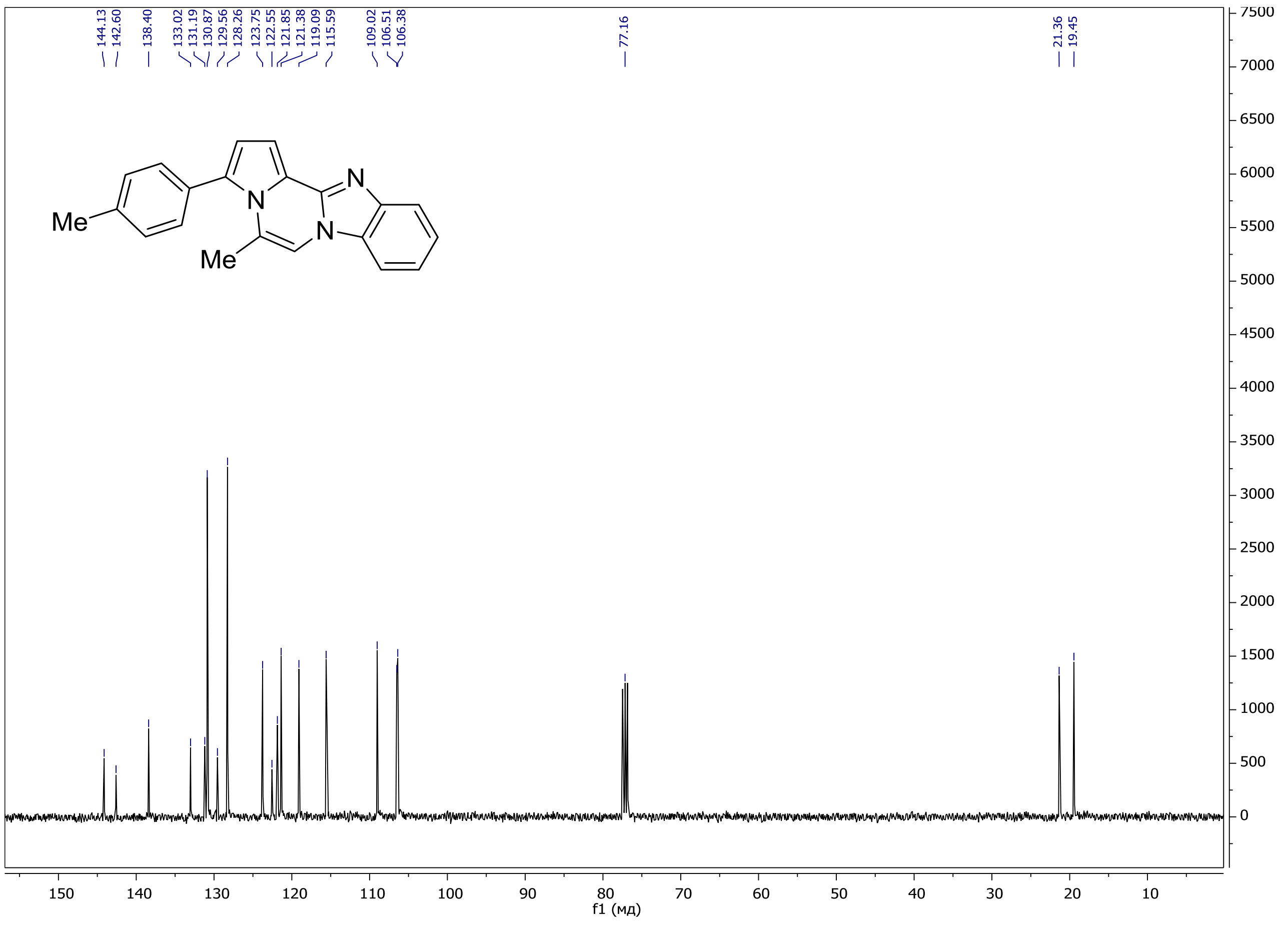

Figure S70. ^{1}H-NMR (400 MHz, $CDCl_3$) spectrum of **6d**

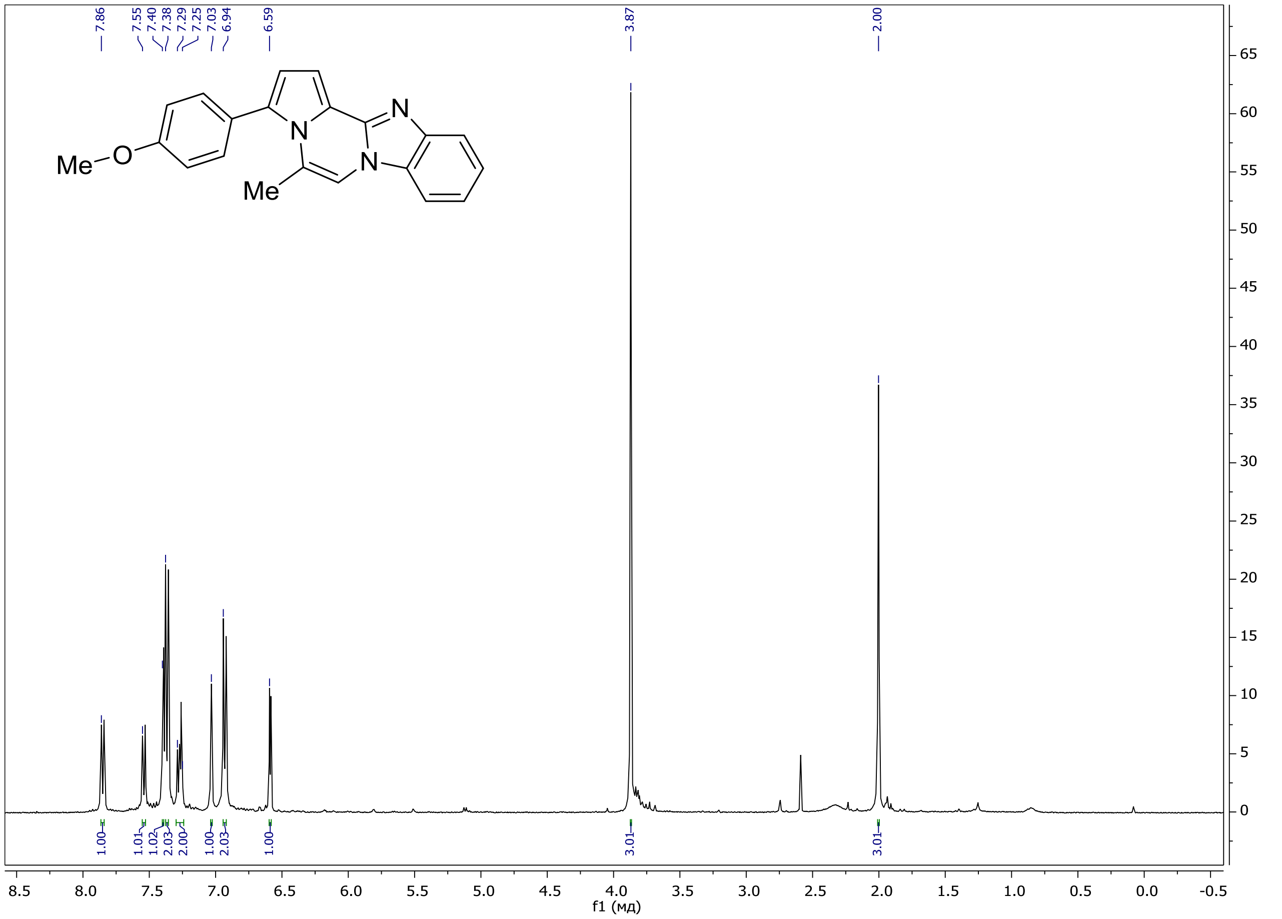

Figure S71. ^{13}C-NMR (100 MHz, $CDCl_3$) spectrum of **6d**

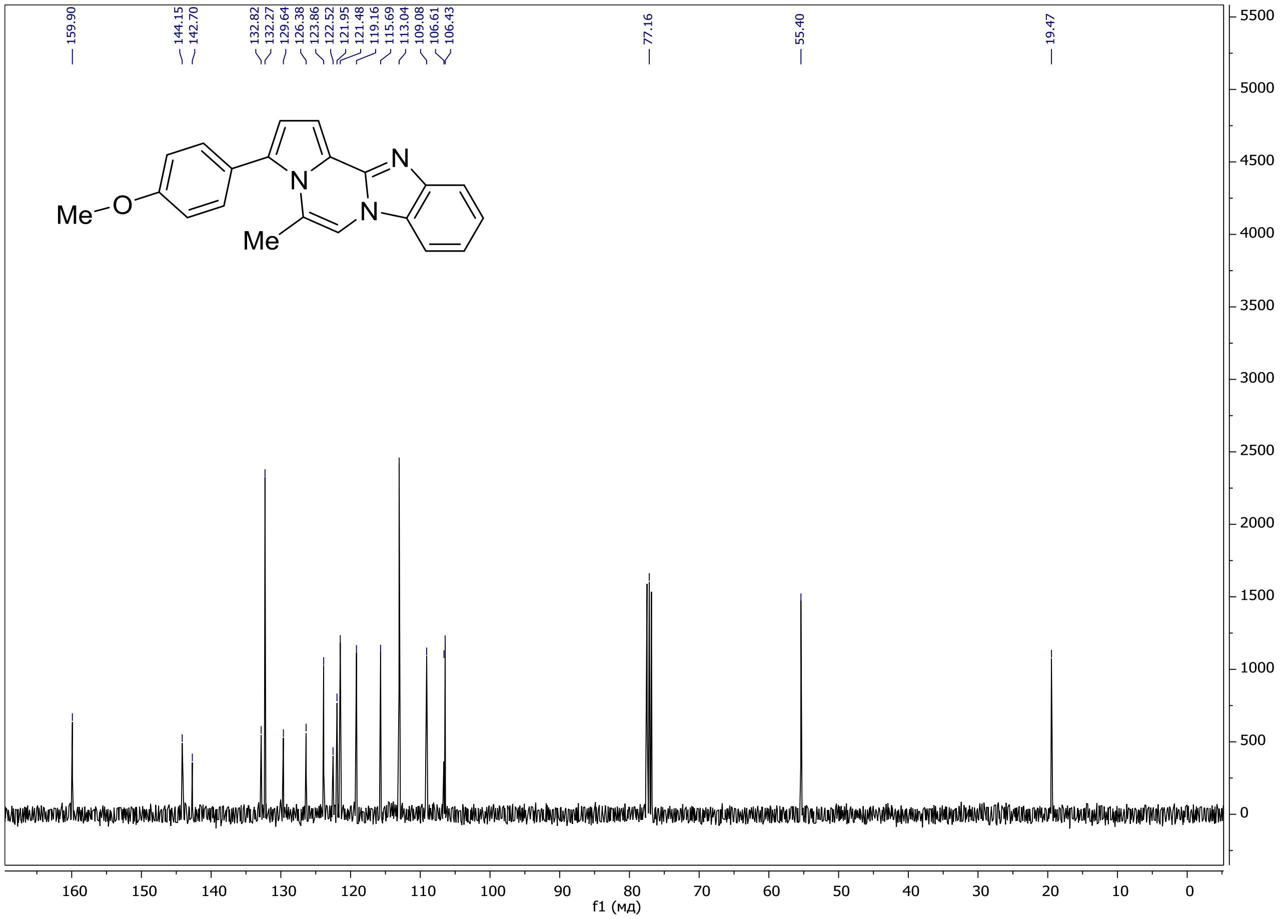

Figure S72. ^{1}H-NMR (400 MHz, $CDCl_3$) spectrum of **6e**

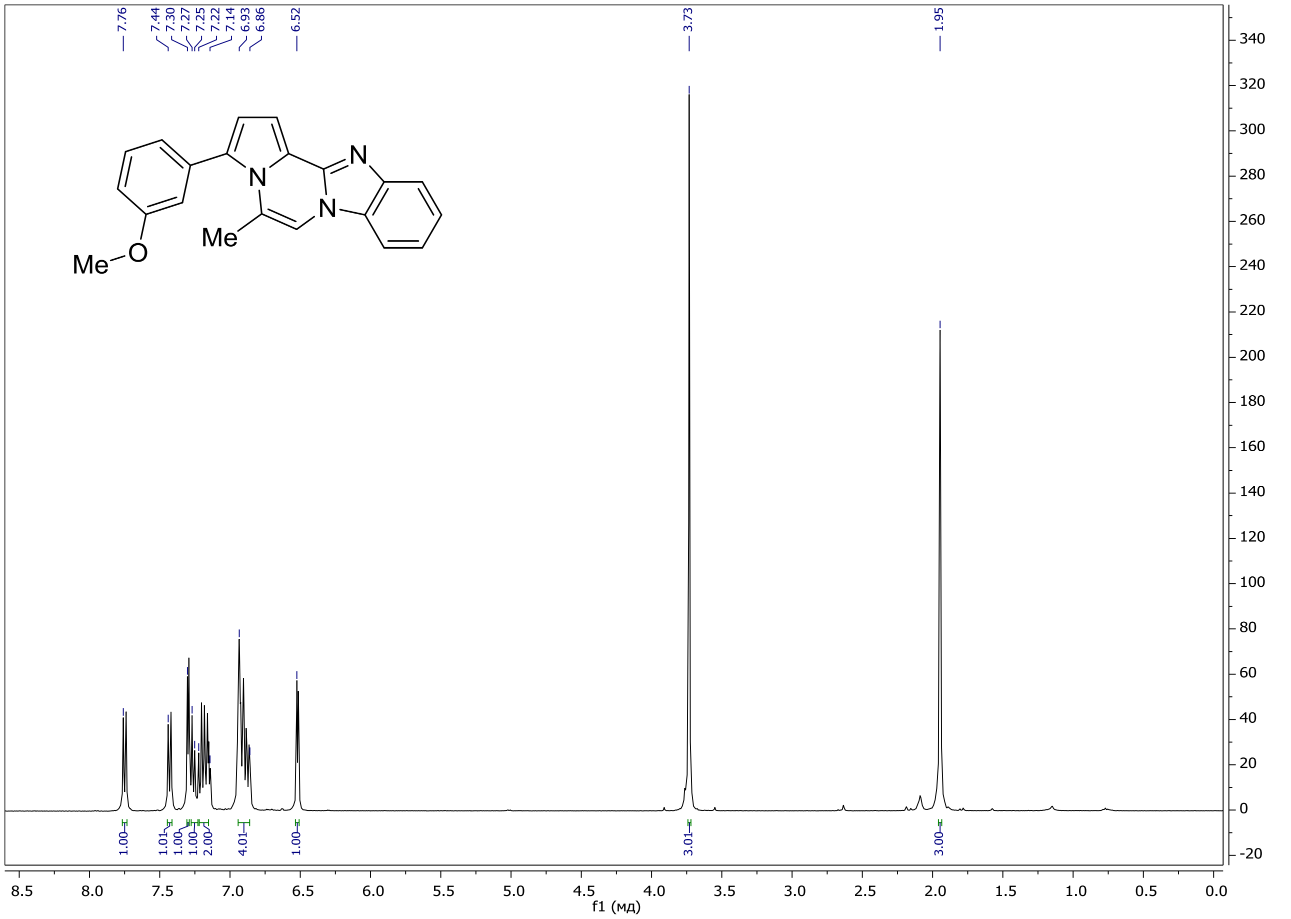

Figure S73. ^{13}C-NMR (100 MHz, $CDCl_3$) spectrum of **6e**

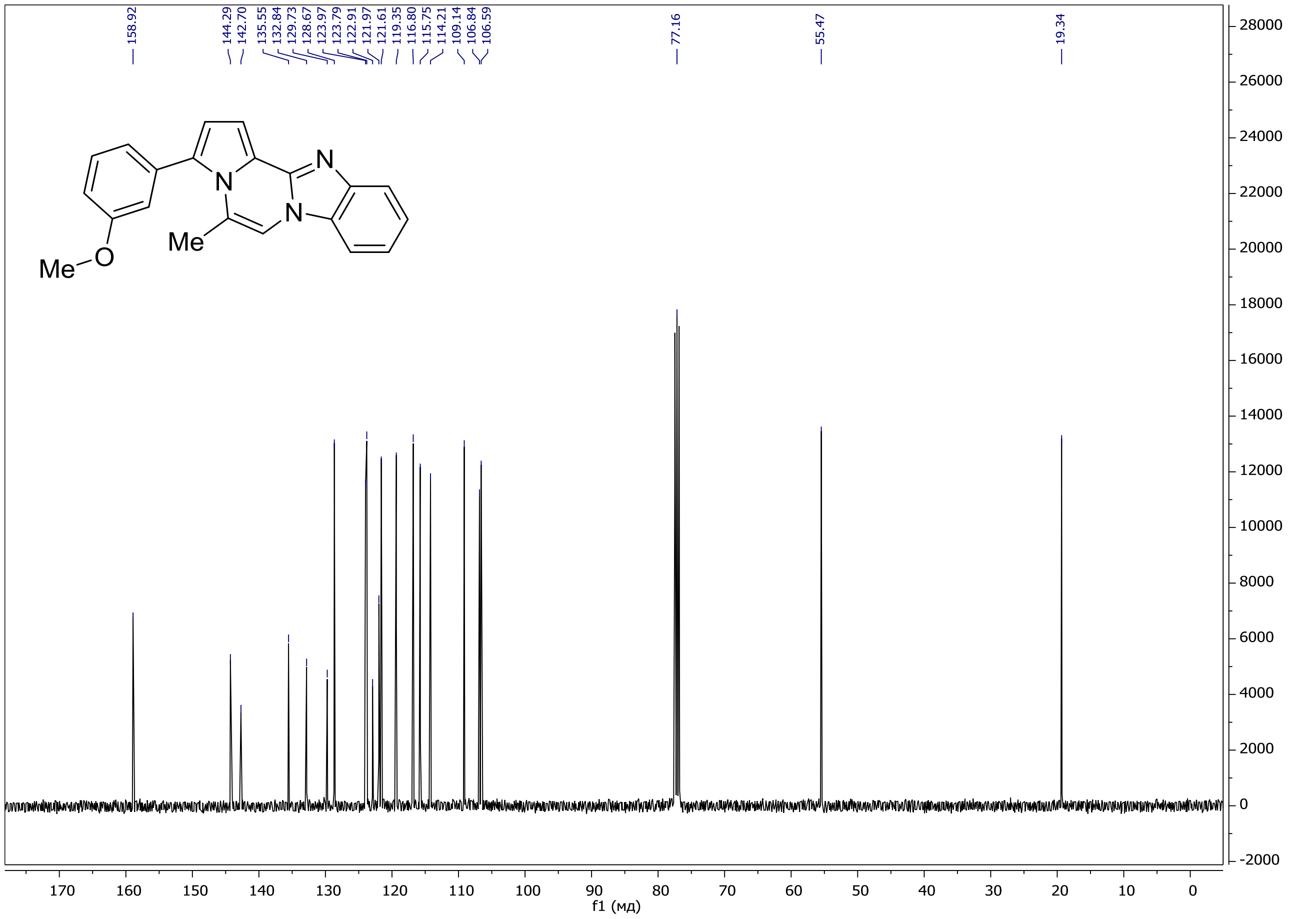

Figure S74. ^{1}H-NMR (400 MHz, $CDCl_3$) spectrum of **6f**

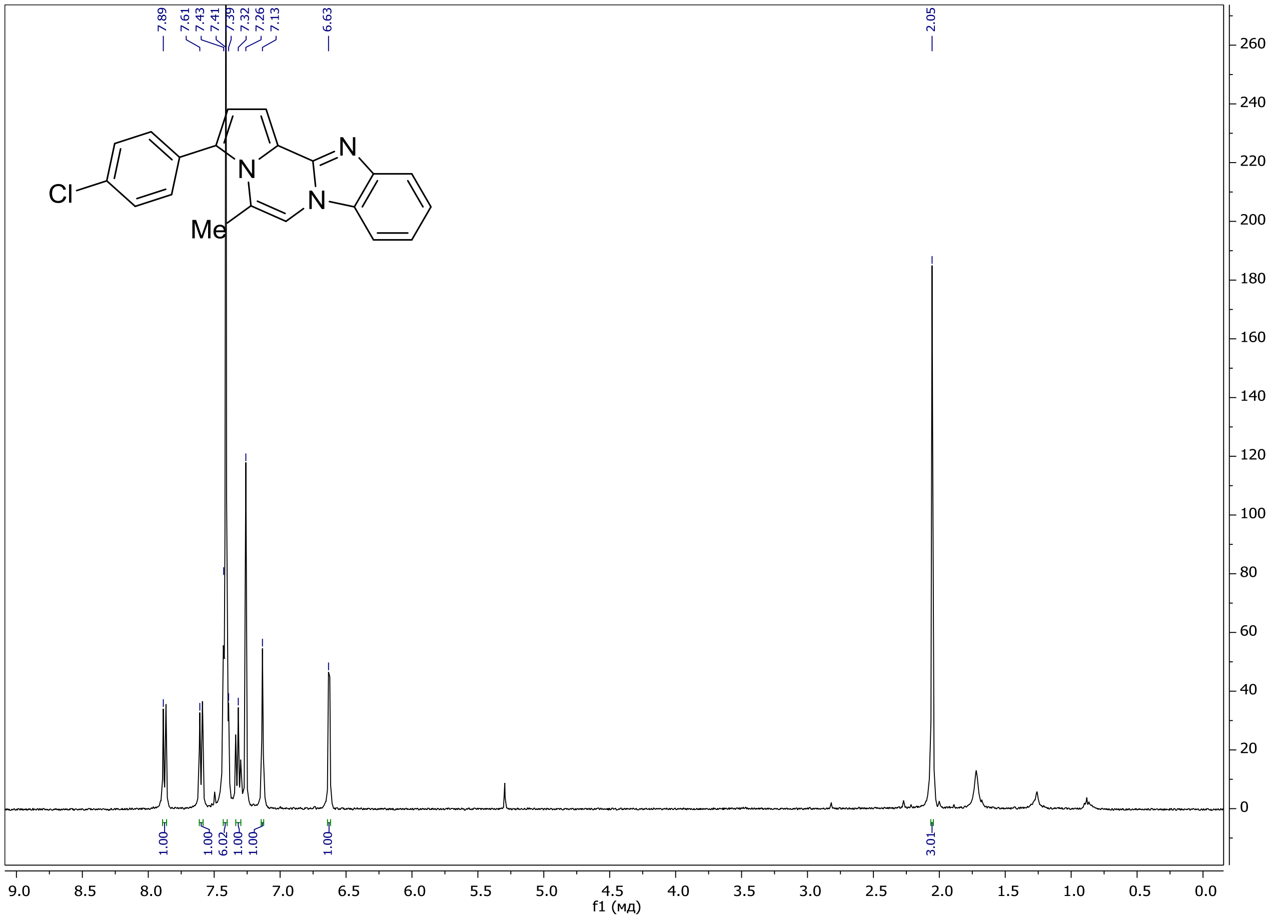

Figure S75. ^{13}C-NMR (100 MHz, $CDCl_3$) spectrum of **6f**

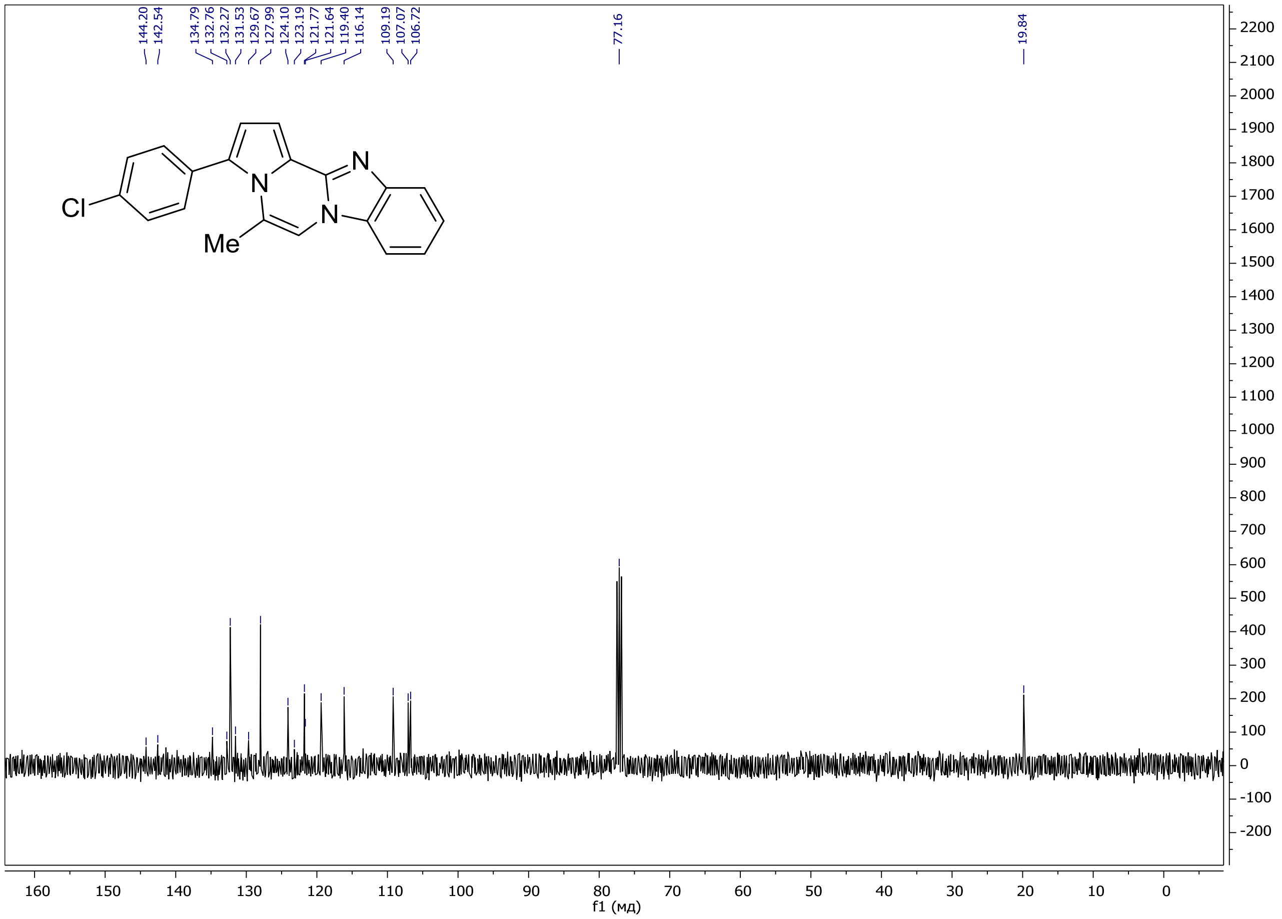

Figure S76. ^{1}H-NMR (400 MHz, $CDCl_3$) spectrum of **6g**

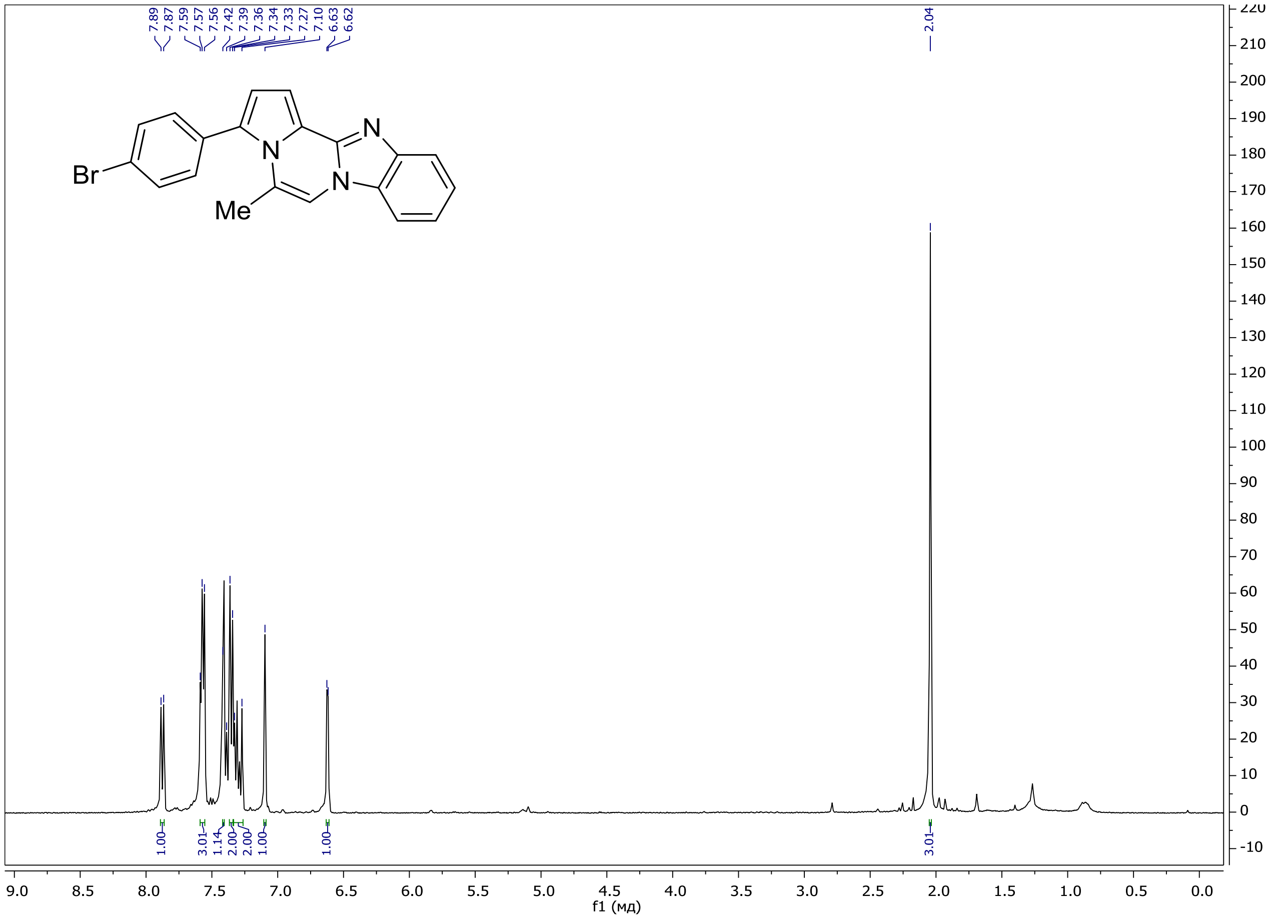

Figure S77. ^{13}C-NMR (100 MHz, $CDCl_3$) spectrum of **6g**

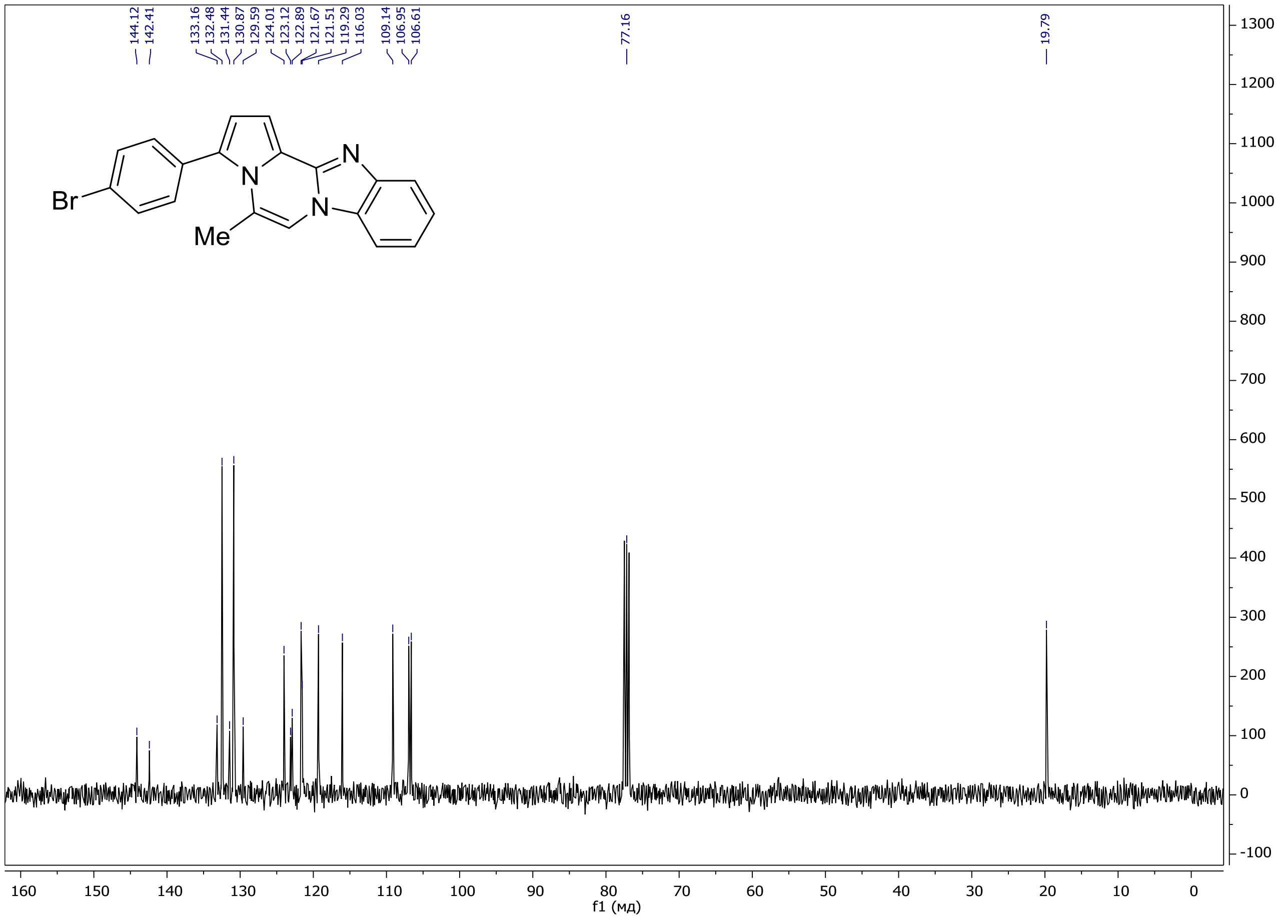

Figure S78. ^{1}H-NMR (400 MHz, $CDCl_3$) spectrum of **6h**

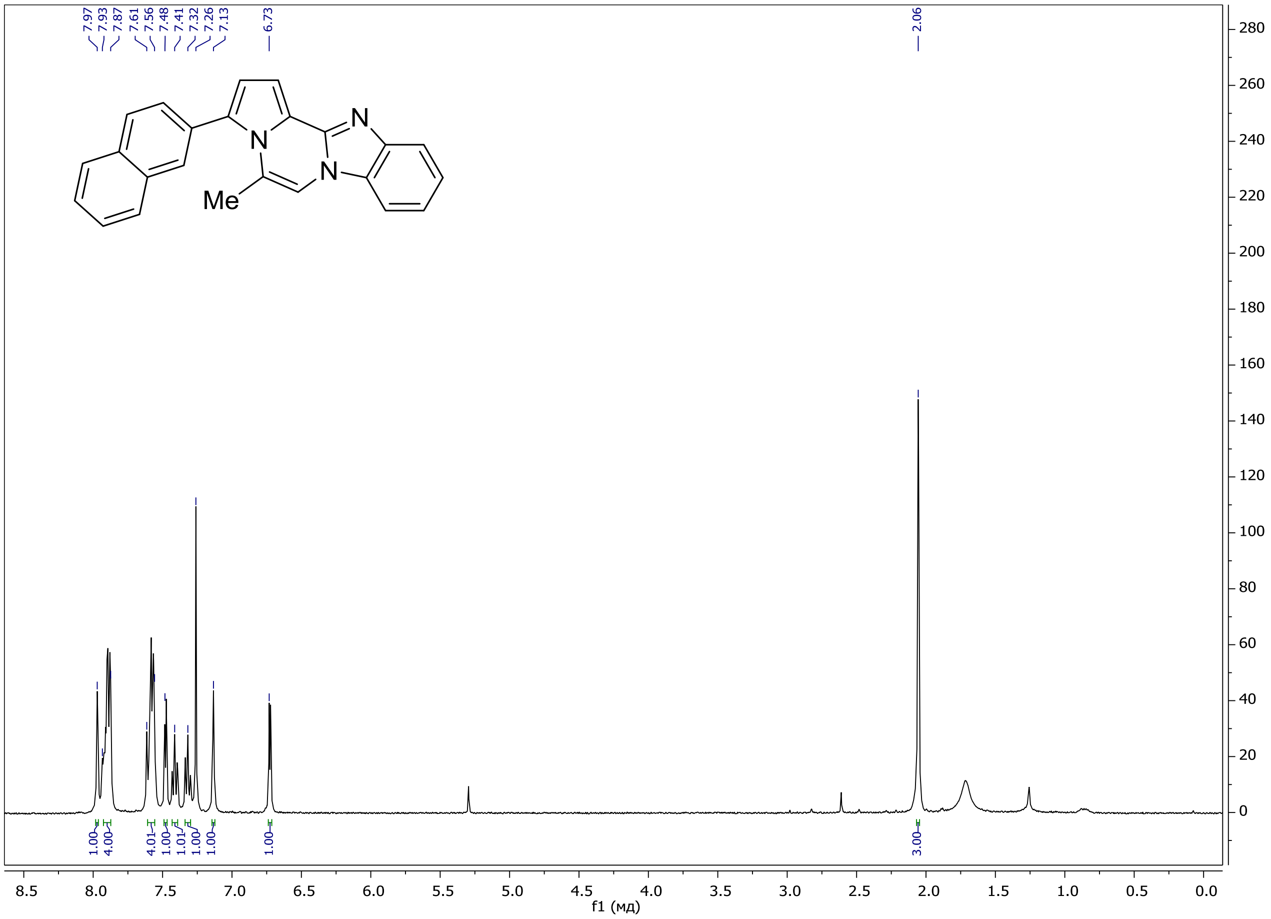

Figure S79. ^{1}H-NMR (400 MHz, $CDCl_3$) spectrum of **6i**

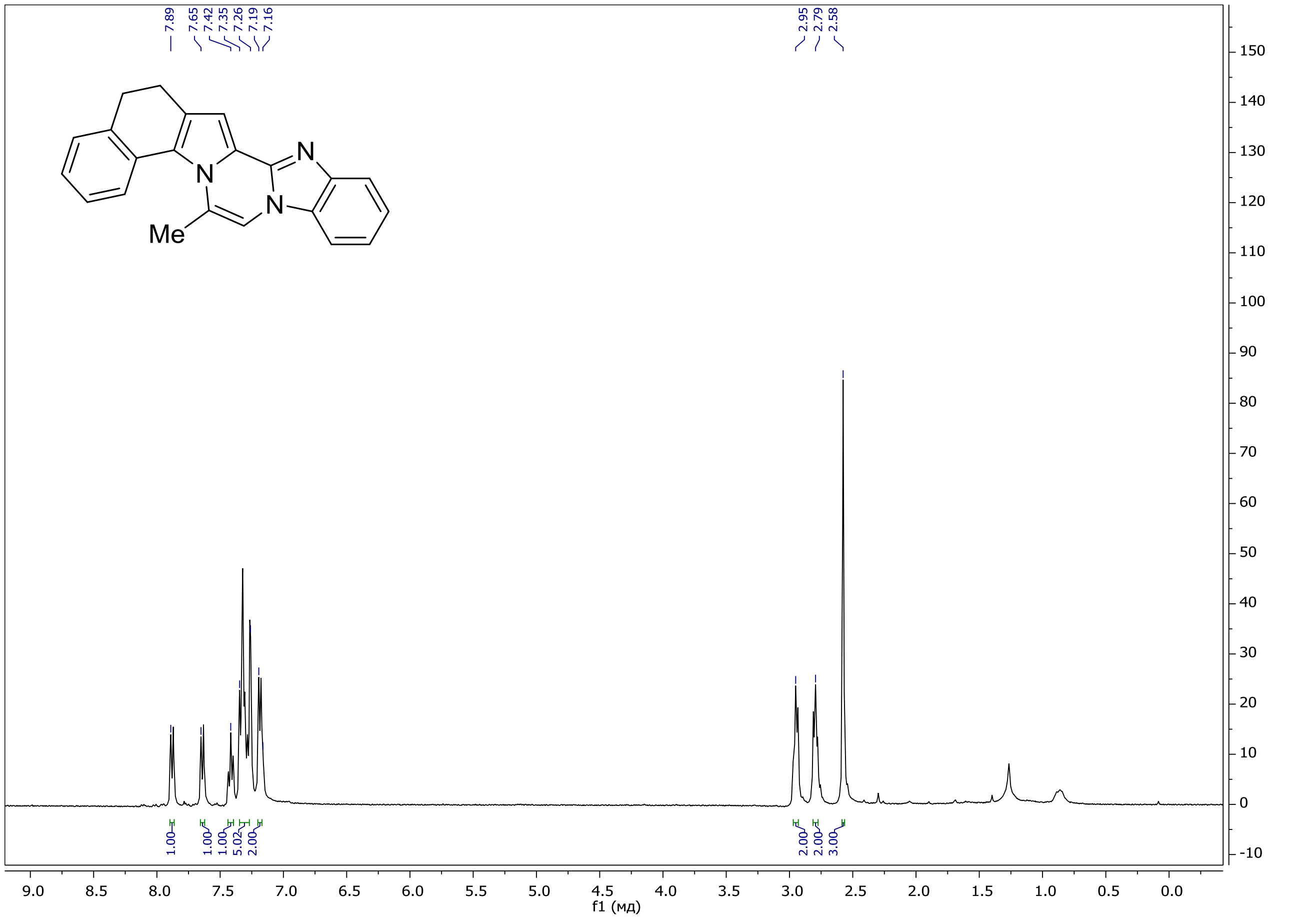

Figure S80. ^{13}C-NMR (100 MHz, $CDCl_3$) spectrum of **6i**

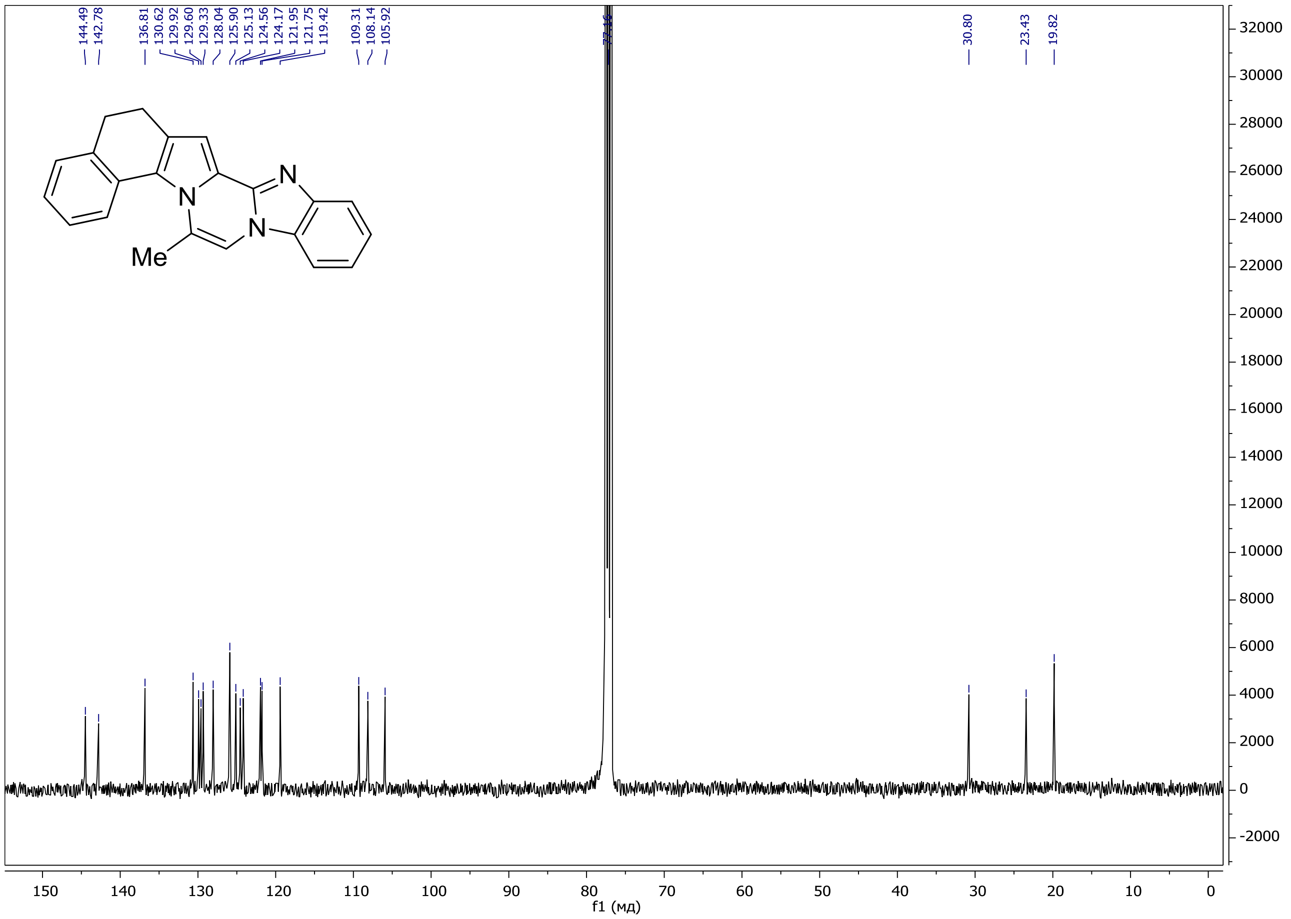

6. Selected 2D NMR spectra

Figure S81. 2D ^{1}H-^{13}C HMBC Spectrum of **4i** ($CDCl_3$)

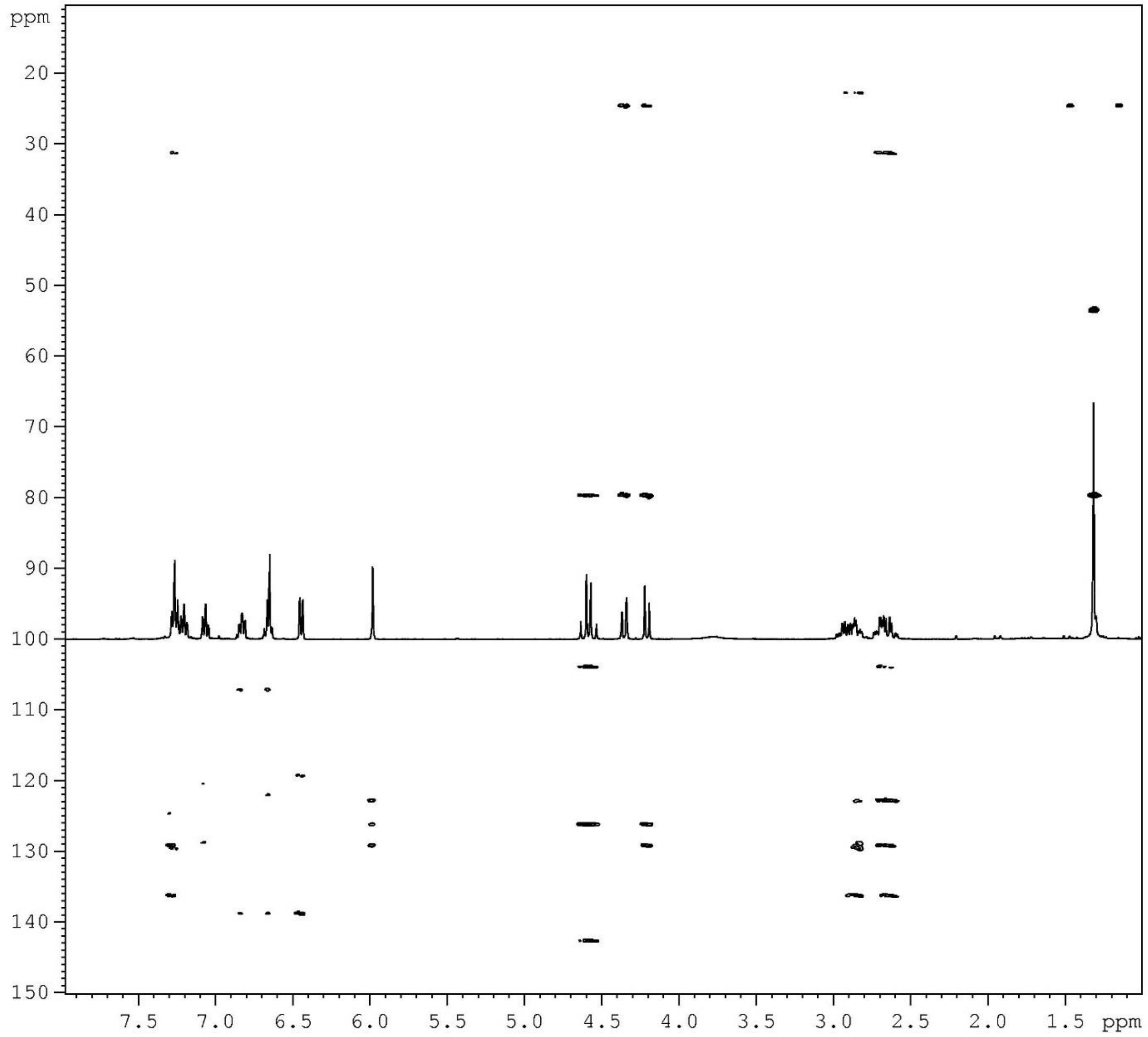

Figure S82. 2D ^{1}H-^{13}C HSQC Spectrum of **4i** ($CDCl_3$)

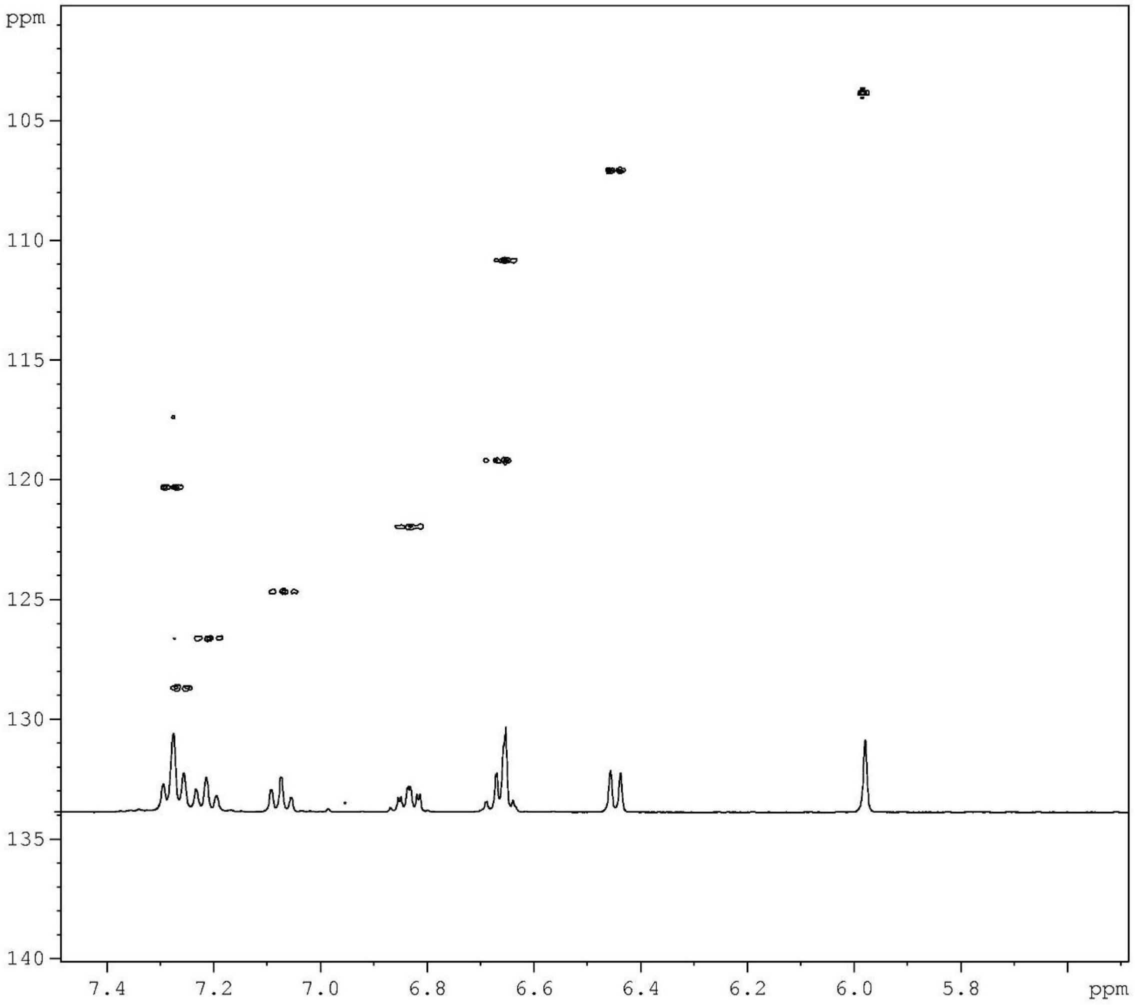

Figure S83. 2D COSY Spectrum of **4i** ($CDCl_3$)

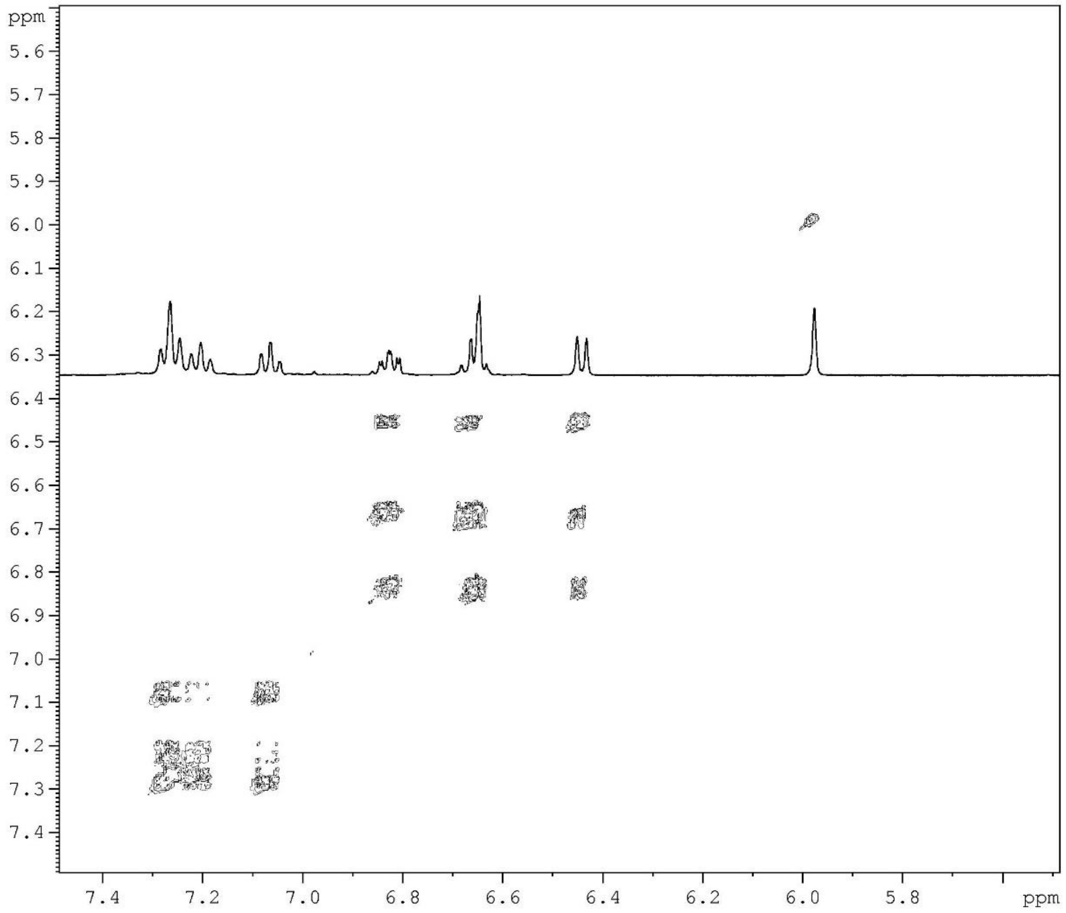

Figure S84. 2D NOESY Spectrum of **4i** ($CDCl_3$)

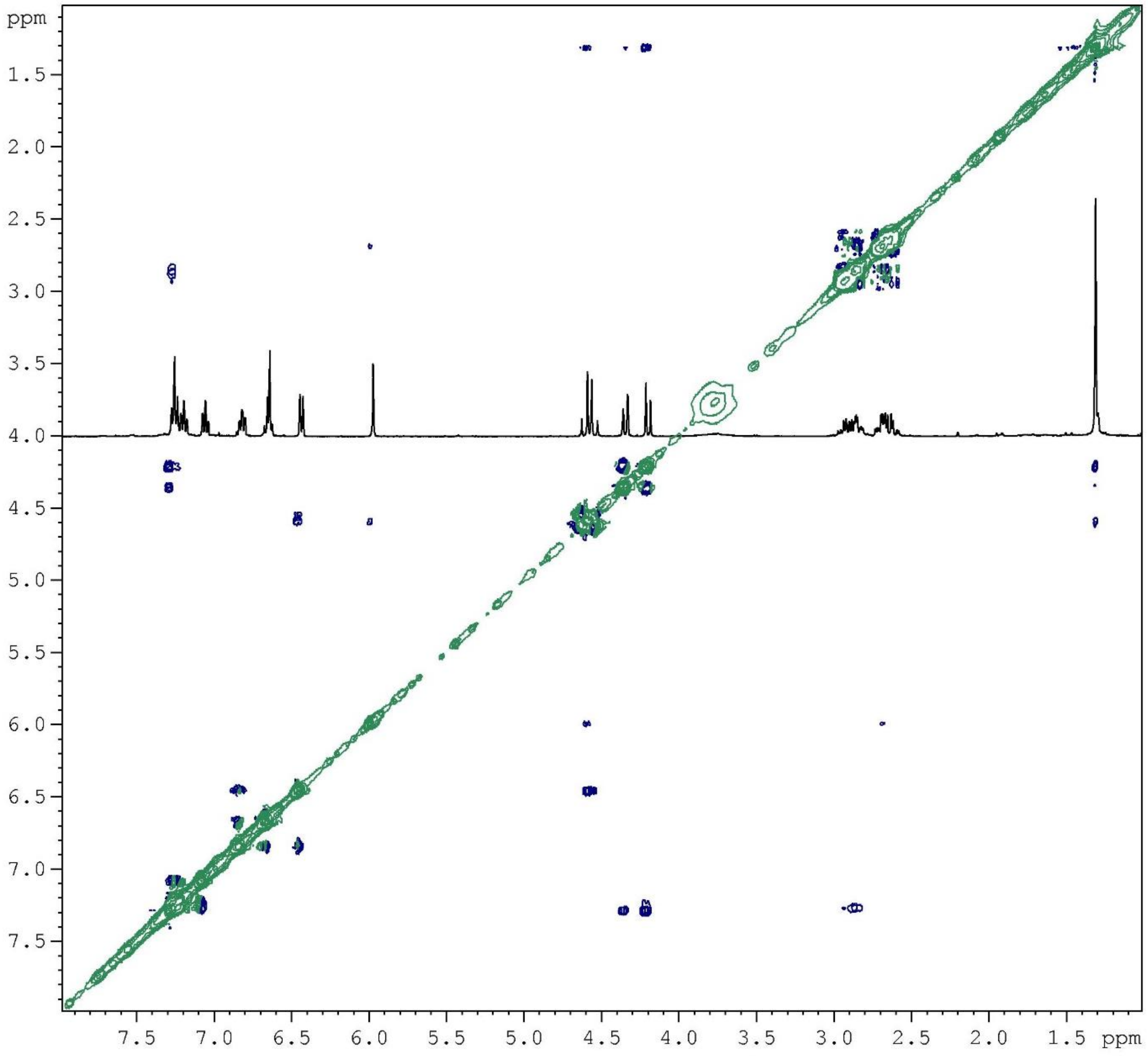

Figure S85. 2D ^{1}H-^{13}C HMBC Spectrum of **6e** ($CDCl_3$)

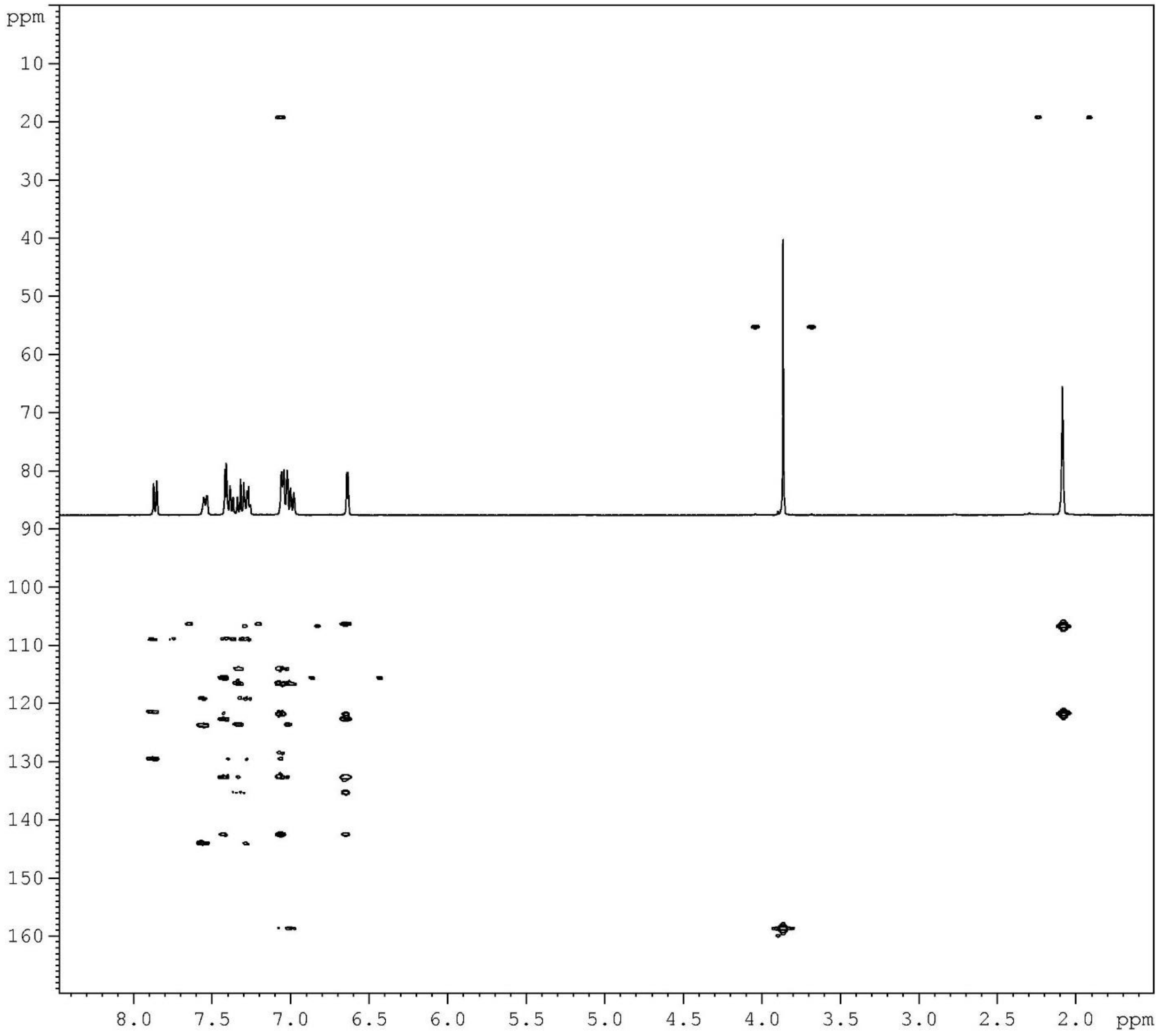

Figure S86. 2D ^{1}H-^{13}C HSQC Spectrum of **6e** ($CDCl_3$)

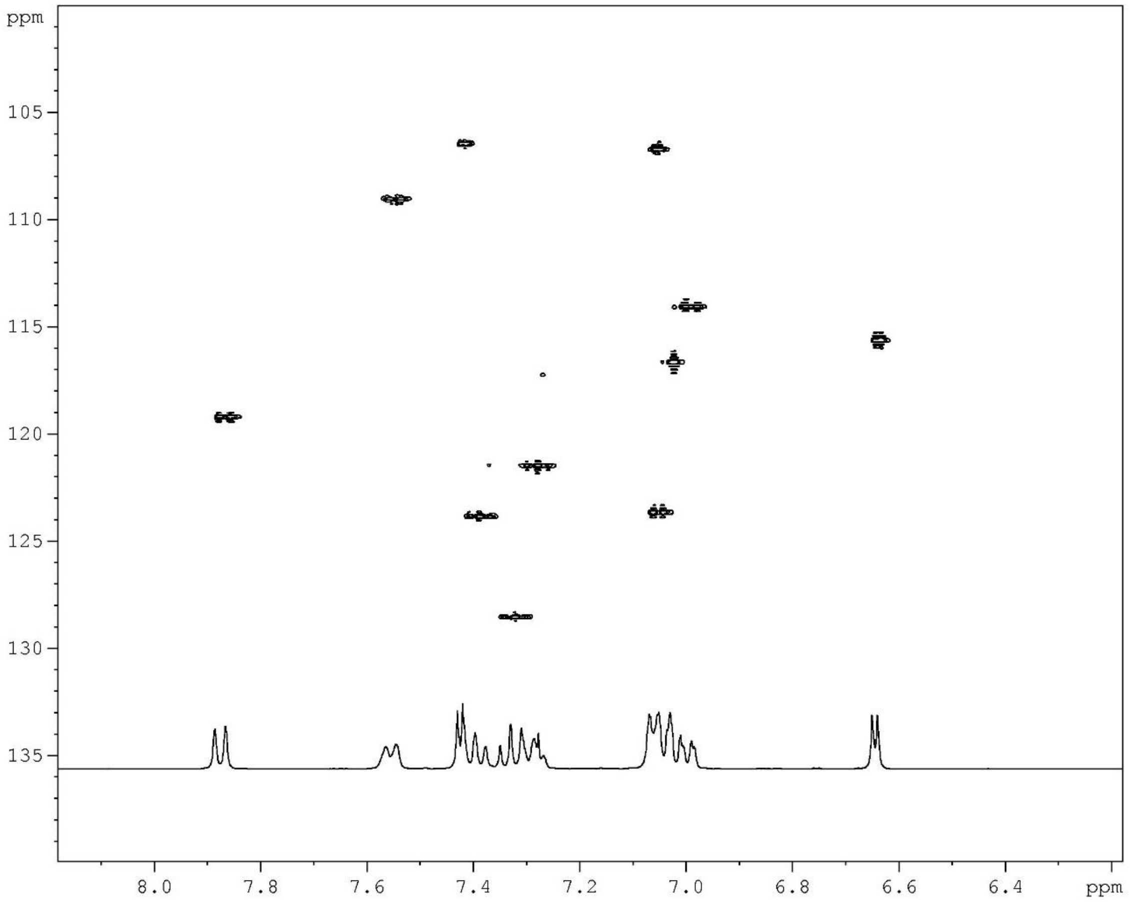

Figure S87. 2D COSY Spectrum of **6e** ($CDCl_3$)

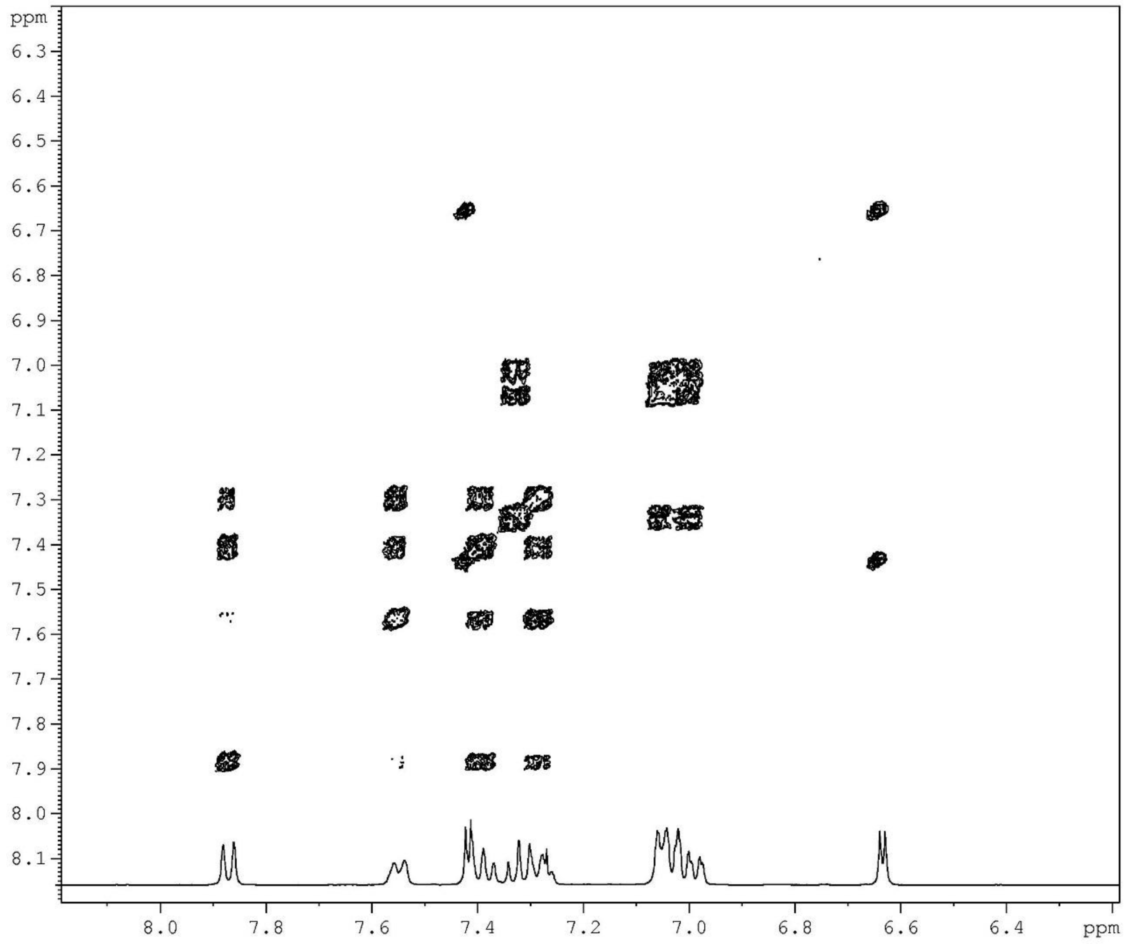

Figure S88. 2D NOESY Spectrum of **6e** ($CDCl_3$)

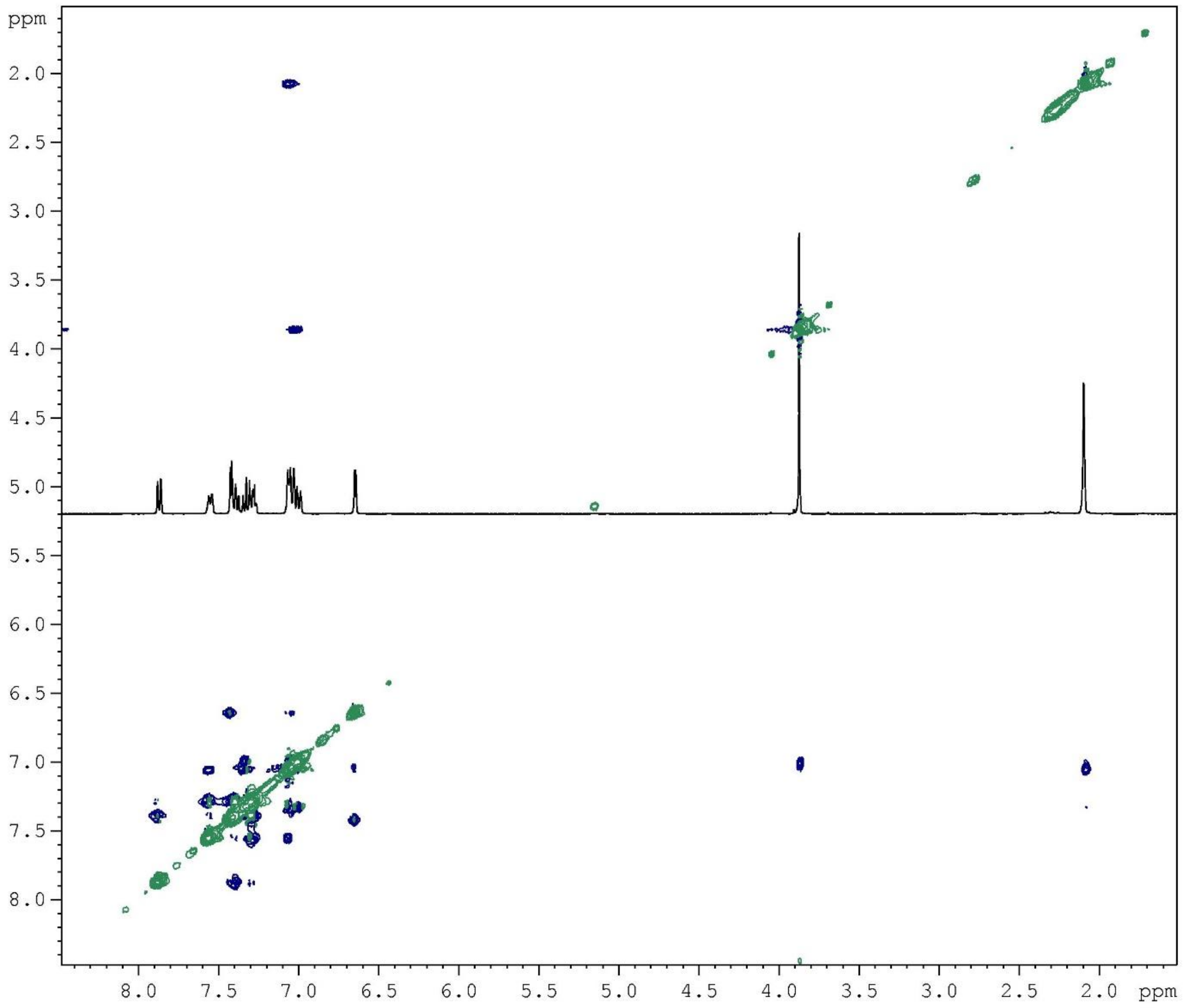

7. X-ray crystallographic data of 3e and 6d

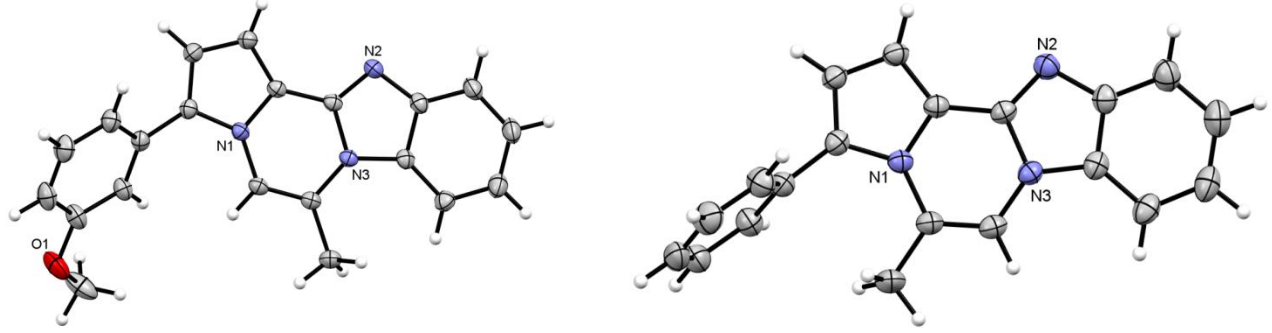

ORTEP plot of compounds *a)* **3e** and *b)* **6b** at 25% thermal ellipsoid probability

Data were collected on a BRUKER D8 VENTURE PHOTON 100 CMOS diffractometer with MoK$_\alpha$ radiation (λ = 0.71073 Å) using the ϕ and ω scans technique. Using Olex2 [1], the structure was solved with the ShelXS [2] structure solution program using Direct Methods and refined with the XL [2] refinement package using Least Squares minimisation. Data were corrected for absorption effects using the multi-scan method (SADABS). All non-hydrogen atoms were refined anisotropically using SHELX [2]. The coordinates of the hydrogen atoms were calculated from geometrical positions.

Crystal data and experimental details are given in Table S1. Selective bond lengths, bond angles and torsion angles are given in Table S2 and S3 (Electronic Supplementary Information).

can be obtained free of charge from The Cambridge Crystallographic Data Centre via http://www.ccdc.cam.ac.uk (accessed on 10, August, 2021).

Table S1. X-ray crystallographic data for compounds **3e** and **6b**

Compound	**3e**	**6b**
CCDC number	2102328	2102327
Empirical formula	$C_{21}H_{17}N_3O$	$C_{20}H_{15}N_3$
Formula weight / g·mol^{-1}	327.38	297.35
Crystal system	Monoclinic	Monoclinic
Space group	*P 2(1)/c*	*P 2(1)/c*
a / Å	10.3012(10)	10.4033(3)
b / Å	22.0073(19)	20.1257(7)
c / Å	7.2627(7)	7.8873(3)
α, β, γ / °	90.00, 90.469(3), 90.00	90.00, 108.427(2), 90.00
Volume / Å^3	1646.4(3)	1566.72(9)
Z	4	4
Density (calculated) / g·cm^{-3}	1.321	1.261
Absorptions coefficient / mm^{-1}	0.083	1.076
Radiation (λ / Å)	MoKα (0.71073)	MoKα (0.71073)
Temperature / K	296(2)	296(2)
2Θ range / °	3.36 – 30.05	2.30 – 30.06

Crystal size / mm	0.57 × 0.45 × 0.39	0.60 × 0.15 × 0.04
Crystal habit	bronze, prism	yellow, needle
F(000)	688	624
Index ranges	-14 ≤ h ≤ 14, -30 ≤ k ≤ 30, -10 ≤ l ≤ 10	-14 ≤ h ≤ 14, -28 ≤ k ≤ 28, -11 ≤ l ≤ 9
Reflections collected	42575	43389
Independent reflections	4804 [Rint = 0.0288, Rsigma = 0.0186]	4583 [Rint = 0.1327, Rsigma = 0.0865]
Data/restraints/parameters	4804/0/228	4583/0/209
R_1 / wR_2 [$I > 2\sigma(I)$]	0.0555 / 0.1388	0.0699 / 0.1630
R_1 / wR_2 (all data)	0.0774 / 0.1522	0.1920 / 0.2098
Goodness-of-fit on F^2	1.067	1.018
Completeness [%]	99.5	99.8
Largest diff. peak and hole / e·Å^{-3}	0.37/ -0.22	0.26/ -0.33
Weight scheme	$w=1/[\sigma^2(F_o^2)+(0.0617P)^2+0.4514P]$ where $P=(F_o^2+2F_c^2)/3$	$w=1/[\sigma^2(F_o^2)+(0.0987P)^2+0.0770P]$ where $P=(F_o^2+2F_c^2)/3$

Table S2. Bond lengths, bond angles and torsion angles for compound **3e**

Bond *l*, Å			Angle ϕ, °				Torsion angle θ, °				
O1	C17	1.381(2)	C21	O1	C17	118.33(15)	O1	C17	C18	C19	178.45(19)
O1	C21	1.353(3)	C11	N3	C12	132.28(12)	N3	C11	C6	N2	0.78(15)
N3	C11	1.3992(17)	C5	N3	C11	105.71(11)	N3	C11	C6	C7	-177.57(14)
N3	C12	1.4098(17)	C5	N3	C12	121.99(11)	N3	C12	C13	N1	1.09(19)
N3	C5	1.3969(17)	C1	N1	C13	130.02(12)	N3	C5	C4	N1	1.32(17)
N1	C13	1.3950(17)	C1	N1	C4	108.62(11)	N3	C5	C4	C3	-179.44(14)
N1	C1	1.3808(18)	C4	N1	C13	121.31(11)	N1	C1	C15	C16	-51.4(2)
N1	C4	1.3893(17)	C5	N2	C6	104.37(12)	N1	C1	C15	C20	130.23(16)
N2	C6	1.384(2)	C10	C9	C8	121.71(17)	N1	C1	C2	C3	-0.55(17)
N2	C5	1.3157(17)	C9	C10	C11	117.25(16)	N1	C4	C3	C2	1.06(16)
C9	C10	1.383(2)	N3	C11	C6	104.61(12)	N2	C5	C4	N1	-179.00(13)
C9	C8	1.388(3)	C10	C11	N3	133.96(14)	N2	C5	C4	C3	0.2(3)
C10	C11	1.391(2)	C10	C11	C6	121.41(14)	C9	C10	C11	N3	177.89(15)
C11	C6	1.408(2)	N3	C12	C14	119.62(12)	C9	C10	C11	C6	-0.3(2)
C12	C13	1.3344(19)	C13	C12	N3	118.26(12)	C9	C8	C7	C6	0.2(3)
C12	C14	1.4935(19)	C13	C12	C14	122.11(13)	C10	C9	C8	C7	0.6(3)
C1	C15	1.468(2)	C12	C13	N1	121.78(12)	C10	C11	C6	N2	179.40(13)
C1	C2	1.376(2)	N1	C1	C15	124.46(13)	C10	C11	C6	C7	1.1(2)
C15	C16	1.394(2)	C2	C1	N1	106.96(13)	C11	N3	C12	C13	178.89(13)

C15	C20	1.395(2)
C16	C17	1.392(2)
C17	C18	1.385(3)
C8	C7	1.377(3)
C7	C6	1.396(2)
C5	C4	1.4160(19)
C4	C3	1.373(2)
C3	C2	1.400(2)
C20	C19	1.381(3)
C19	C18	1.371(3)

C2	C1	C15	128.51(14)
C16	C15	C1	122.04(13)
C20	C15	C1	118.32(14)
C20	C15	C16	119.62(14)
C17	C16	C15	119.24(15)
O1	C17	C16	123.85(16)
O1	C17	C18	115.51(16)
C18	C17	C16	120.64(17)
C7	C8	C9	121.45(16)
C8	C7	C6	118.06(17)
N2	C6	C11	111.59(12)
N2	C6	C7	128.27(15)
C7	C6	C11	120.11(15)
N3	C5	C4	118.53(12)
N2	C5	N3	113.70(12)
N2	C5	C4	127.77(13)
N1	C4	C5	118.09(12)
C3	C4	N1	108.38(12)
C3	C4	C5	133.52(13)
C4	C3	C2	106.78(13)
C1	C2	C3	109.24(14)
C19	C20	C15	120.06(17)
C18	C19	C20	120.64(17)
C19	C18	C17	119.79(17)

C11	N3	C12	C14	-1.9(2)
C11	N3	C5	N2	-0.19(15)
C11	N3	C5	C4	179.53(11)
C12	N3	C11	C10	2.7(2)
C12	N3	C11	C6	-178.95(13)
C12	N3	C5	N2	178.59(11)
C12	N3	C5	C4	-1.69(17)
C13	N1	C1	C15	1.6(2)
C13	N1	C1	C2	178.77(13)
C13	N1	C4	C5	0.17(18)
C13	N1	C4	C3	-179.25(12)
C1	N1	C13	C12	-178.75(13)
C1	N1	C4	C5	178.00(11)
C1	N1	C4	C3	-1.42(15)
C1	C15	C16	C17	-178.78(15)
C1	C15	C20	C19	178.07(16)
C15	C1	C2	C3	176.46(14)
C15	C16	C17	O1	-178.23(17)
C15	C16	C17	C18	1.0(3)
C15	C20	C19	C18	0.5(3)
C16	C15	C20	C19	-0.3(3)
C16	C17	C18	C19	-0.9(3)
C21	O1	C17	C16	-4.8(3)
C21	O1	C17	C18	175.9(2)
C8	C9	C10	C11	-0.5(3)
C8	C7	C6	N2	-179.07(16)
C8	C7	C6	C11	-1.0(2)
C6	N2	C5	N3	0.65(15)
C6	N2	C5	C4	-179.04(13)
C5	N3	C11	C10	-178.72(15)
C5	N3	C11	C6	-0.35(13)
C5	N3	C12	C13	0.48(18)
C5	N3	C12	C14	179.72(12)
C5	N2	C6	C11	-0.88(16)

C5	N2	C6	C7	177.29(16)
C5	C4	C3	C2	-178.24(14)
C4	N1	C13	C12	-1.44(19)
C4	N1	C1	C15	-175.95(13)
C4	N1	C1	C2	1.20(15)
C4	C3	C2	C1	-0.32(17)
C2	C1	C15	C16	132.08(17)
C2	C1	C15	C20	-46.3(2)
C14	C12	C13	N1	-178.13(12)
C20	C15	C16	C17	-0.4(2)
C20	C19	C18	C17	0.1(3)

Table S3. Bond lengths, bond angles and torsion angles for compound **6b**

Bond *l*, Å			Angle ϕ, ˚				Torsion angle θ, ˚				
N1	C1	1.392(3)	C1	N1	C4	107.90(16)	N1	C1	C2	C3	-0.7(2)
N1	C4	1.403(2)	C1	N1	C13	130.84(17)	N1	C4	C5	N2	-177.35(18)
N1	C13	1.416(2)	C4	N1	C13	121.23(17)	N1	C4	C5	N3	1.0(2)
N2	C5	1.323(2)	C5	N2	C6	104.25(18)	N1	C4	C3	C2	0.7(2)
N2	C6	1.387(3)	C5	N3	C12	121.91(18)	N1	C13	C12	N3	0.1(3)
N3	C5	1.381(2)	C5	N3	C11	106.37(17)	N2	C6	C7	C8	177.9(2)
N3	C12	1.385(3)	C12	N3	C11	131.71(18)	N2	C6	C11	N3	0.2(2)
N3	C11	1.391(3)	C19	C18	C17	119.9(2)	N2	C6	C11	C10	-178.78(18)
C18	C19	1.368(4)	C18	C19	C20	119.6(2)	N3	C11	C10	C9	-177.8(2)
C18	C17	1.371(4)	C15	C20	C19	121.5(2)	C18	C19	C20	C15	1.4(4)
C19	C20	1.380(3)	C20	C15	C1	122.53(19)	C18	C17	C16	C15	0.5(4)
C20	C15	1.380(3)	C20	C15	C16	118.1(2)	C19	C18	C17	C16	-0.9(4)
C15	C1	1.482(3)	C16	C15	C1	119.3(2)	C19	C20	C15	C1	-177.5(2)
C15	C16	1.390(3)	N1	C1	C15	127.08(18)	C19	C20	C15	C16	-1.8(3)
C1	C2	1.372(3)	C2	C1	N1	107.11(18)	C20	C15	C1	N1	-80.0(3)
C4	C5	1.416(3)	C2	C1	C15	125.8(2)	C20	C15	C1	C2	102.0(3)
C4	C3	1.372(3)	N1	C4	C5	118.96(17)	C20	C15	C16	C17	0.8(4)
C6	C7	1.389(3)	C3	C4	N1	108.24(17)	C15	C1	C2	C3	177.59(19)
C6	C11	1.403(3)	C3	C4	C5	132.72(19)	C1	N1	C4	C5	175.99(16)

C7	C8	1.384(4)
C8	C9	1.390(4)
C17	C16	1.380(3)
C13	C12	1.331(3)
C13	C14	1.492(3)
C11	C10	1.386(3)
C10	C9	1.383(3)
C3	C2	1.396(3)

N2	C5	N3	113.38(19)
N2	C5	C4	129.00(19)
N3	C5	C4	117.61(18)
N2	C6	C7	129.6(2)
N2	C6	C11	111.16(19)
C7	C6	C11	119.2(2)
C8	C7	C6	118.0(3)
C7	C8	C9	121.6(3)
C18	C17	C16	120.6(2)
C17	C16	C15	120.2(2)
N1	C13	C14	120.3(2)
C12	C13	N1	118.14(19)
C12	C13	C14	121.5(2)
C13	C12	N3	122.10(19)
N3	C11	C6	104.84(18)
C10	C11	N3	131.8(2)
C10	C11	C6	123.3(2)
C9	C10	C11	116.0(2)
C10	C9	C8	121.8(3)
C4	C3	C2	107.16(18)
C1	C2	C3	109.57(19)

C1	N1	C4	C3	-1.2(2)
C1	N1	C13	C12	-176.11(18)
C1	N1	C13	C14	3.3(3)
C1	C15	C16	C17	176.7(2)
C4	N1	C1	C15	-177.12(19)
C4	N1	C1	C2	1.1(2)
C4	N1	C13	C12	2.0(3)
C4	N1	C13	C14	-178.60(19)
C4	C3	C2	C1	0.0(2)
C5	N2	C6	C7	-178.2(2)
C5	N2	C6	C11	0.1(2)
C5	N3	C12	C13	-1.6(3)
C5	N3	C11	C6	-0.39(19)
C5	N3	C11	C10	178.4(2)
C5	C4	C3	C2	-175.9(2)
C6	N2	C5	N3	-0.4(2)
C6	N2	C5	C4	178.01(19)
C6	C7	C8	C9	0.3(4)
C6	C11	C10	C9	0.8(3)
C7	C6	C11	N3	178.65(19)
C7	C6	C11	C10	-0.3(3)
C7	C8	C9	C10	0.3(4)
C17	C18	C19	C20	0.0(4)
C16	C15	C1	N1	104.3(3)
C16	C15	C1	C2	-73.7(3)
C13	N1	C1	C15	1.2(3)
C13	N1	C1	C2	179.42(18)
C13	N1	C4	C5	-2.5(3)
C13	N1	C4	C3	-179.65(16)
C12	N3	C5	N2	179.60(16)
C12	N3	C5	C4	1.0(3)
C12	N3	C11	C6	-179.36(19)
C12	N3	C11	C10	-0.5(3)
C11	N3	C5	N2	0.5(2)

C11	N3	C5	C4	-178.08(16)
C11	N3	C12	C13	177.26(18)
C11	C6	C7	C8	-0.2(3)
C11	C10	C9	C8	-0.8(3)
C3	C4	C5	N2	-1.0(4)
C3	C4	C5	N3	177.29(19)
C14	C13	C12	N3	-179.4(2)

8. References

1. Dolomanov, O.V.; Bourhis, L.J.; Gildea, R.J.; Howard, J.A.K.; Puschmann, H. *OLEX2*: a complete structure solution, refinement and analysis program. *J. Appl. Cryst.* **2009**, *42*, 339–341. https://doi.org/10.1107/S0021889808042726.
2. Sheldrick, G.M. A short history of *SHELX*. *Acta Cryst*. **2008**, *64*, 112–122. https://doi.org/10.1107/S0108767307043930.
